# Electrochemical Sulfonylation in Deep Eutectic Solvents Enables the Sustainable Synthesis of 2‐Quinoline Sulfones

**DOI:** 10.1002/cssc.202501779

**Published:** 2025-10-20

**Authors:** Darío Adsuar, Xavier Marset, Diego J. Ramón, Néstor Guijarro

**Affiliations:** ^1^ Institute of Electrochemistry University of Alicante Apdo. 99 03080 Alicante Spain; ^2^ Institute of Organic Synthesis and Department of Organic Chemistry University of Alicante Apdo. 99 03080 Alicante Spain

**Keywords:** deep eutectic solvents, electrosynthesis, solvent effects, sulfone, sustainability

## Abstract

Organic electrosynthesis is gaining momentum, driven by the inherent advantages of using electricity in place of stoichiometric chemical oxidants, such as the improved atom efficacy, the minimization of waste, and the lower cost. However, electrosynthesis methods rely on volatile organic solvents such as acetonitrile to solubilize the reagents, combined with expensive and nonrecyclable electrolytes, which compromise the environmental and economic viability of the approach. Taking the electrosynthesis of 2‐arylsulfonylquinolines as representative case, the aforementioned issues by incorporating a deep eutectic solvent that functions simultaneously as the reaction medium and supporting electrolyte are addressed. The method delivers excellent yields, and products are isolated at gram scale via simple water washing and filtration. Interestingly, the eutectic solvent is recovered and reused for up to five cycles without significant loss in reaction yields. In a more general vein, this strategy not only eliminates volatile organic solvents throughout both the reaction and purification stages, but also integrates a recyclable solvent–electrolyte system, there for enabling a fully sustainable electrosynthetic process.

## Graphical abstract

The use of a deep eutectic solvent (DES) in sulfonylation electrosynthesis enables the high‐yield and scalable production of 2‐arylsulfonylquinolines without the need of volatile organic compounds. Acting as reaction medium and removing the need of a supporting electrolyte, the DES affords excellent yields across a broad scope of compounds, allows simple water‐based isolation of products, and can be efficiently reused.

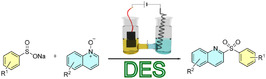

## 1 Introduction

Organic electrosynthesis is a flourishing field, yet with important challenges that need to be addressed. The interest in this topic has surged, driven by the growing demand for sustainable synthetic methods, with electrosynthesis offering a green alternative to traditional redox chemistry by using electricity as the primary reagent,^[^ ^1^ ^]^ thus eliminating the need for stoichiometric oxidants or reductants and aligning with key green chemistry principles.^[^ ^2^ ^]^ In addition, the economic cost of a mol of electrons is by far cheaper than the use of the corresponding amount of a chemical oxidant or reductant.^[^ ^3^ ^]^ Beyond its environmental advantages, electrosynthesis enables precise control over redox processes. By tuning the applied potential, specific substrates can be selectively activated while avoiding overoxidation or side reactions,^[^ ^4^ ^]^ thus expanding the chemoselectivity and functional group tolerance of synthetic methodologies.^[^ ^5^ ^]^ However, a major limitation of organic electrosynthesis lies in the choice of reaction media. While conventional electrochemical processes are commonly conducted in aqueous solutions, many organic molecules are either poorly soluble,^[^ ^6^ ^]^ unstable in water, or exhibit redox potentials incompatible with water's electrochemical window.^[^ ^7^ ^]^ As a result, organic electrosynthesis typically requires aprotic solvents combined with supporting electrolytes, often based on quaternary ammonium salts with weakly coordinating anions with small ionic radii.^[^ ^8^ ^]^ However, these electrolytes are relatively expensive, toxic, and difficult to recover. Moreover, the environmental and safety concerns associated with conventional organic solvents (derived largely from petrochemical sources and characterized by high volatility, flammability, and toxicity) have prompted the search for greener alternatives.^[^ ^9^, ^10^ ^]^

In this context, deep eutectic solvents (DESs) have emerged as promising candidates for sustainable organic electrosynthesis. DESs are mixtures of two or more components that form a mixture that is fluid at a specified mixing ratio at the desired temperature.^[^ ^11^, ^12^ ^]^ Specifically, Type III DESs, composed of an organic salt (e.g., choline chloride) and a hydrogen bond donor (e.g., acids and alcohols), are particularly attractive due to their biodegradability, low vapor pressure, and potential for biorenewability. These solvents often exhibit good solubility for organic and inorganic compounds and inherent ionic conductivity, allowing electrochemical reactions to proceed without supporting electrolytes. However, instead of being composed purely of ionic compounds, DESs are formed by mixing salts with an important portion of neutral molecules, which can control the charge distribution and oriented dipoles at the electrode/solution interface.^[^ ^13^ ^]^ In fact, DESs were initially applied in the electrodeposition/electropolishing of metals,^[^ ^14^ ^]^ but their potential for organic electrosynthesis has only recently begun to be explored. Despite challenges (such as high viscosity, limited electrochemical stability, and susceptibility to degradation under certain conditions), emerging studies reveal their capabilities. To the best of our knowledge, electro‐organic synthetic procedures described in DESs are limited to very recent reports describing the electrochemical allylation of organic substrates,^[^ ^15^ ^]^ the electrooxidation of 5‐hydroxymethylfurfural,^[^ ^16^ ^]^ and an enantioselective carboxylation of (1‐bromoethyl)benzenes.^[^ ^17^ ^]^ These early reports suggest that DESs could unlock new, sustainable pathways for organic electrosynthesis, although a better understanding on the role of DES as electrosynthetic medium is required and more complex transformations are needed to prove to the community the potential of this field.

**Figure 1:**
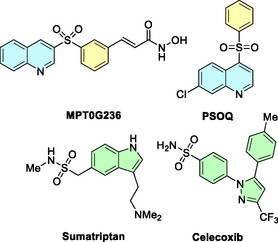
Structures of relevant drugs containing a sulfonyl group.

Based on our group's previous experiences on the synthesis of sulfones in the context of sustainable chemistry,^[^ ^18^, ^19^, ^20^, ^21^ ^]^ we decided to explore an electrocatalyzed process to access these compounds without the traditional environmental cost of synthetic procedures relying on toxic halogenated compounds and stoichiometric oxidant materials. Moreover, recent studies have also shown the applicability of arylsulfinate salts in DESs to obtain γ‐keto sulfones.^[^ ^22^ ^]^ Sulfur‐containing molecules are prevalent in pharmaceuticals and biologically active compounds,^[^ ^23^, ^24^ ^]^ including sulfones such as **MPT0G236**, a potential anticancer drug,^[^ ^25^ ^]^ **PSOQ**, an antinociceptive drug,^[^ ^26^ ^]^ or important sulfonamides such as sumatriptan or celecoxib (**Figure** **1**).

The synthesis of diaryl sulfones using electrosynthesis is a novel topic, yet not completely unexplored. Some reports make use of sulfonylhydrazides, which suffer a denitrogenation process to afford a sulfonyl radical.^[^ ^27^ ^]^ However, with the goal of improving atom economy and use readily available compounds we were more interested in the use of sulfinate salts. Some reports have proven the possibility of coupling sulfinate with aromatic anilines,^[^ ^28^ ^]^ pyridines,^[^ ^29^ ^]^ or quinoline *N*‐oxides.^[^ ^30^ ^]^ All of the aforementioned reports involving the use of sulfinate salts as starting materials invariably use a mixture of acetonitrile with aqueous solutions as reaction media and TBABF_4_ as organic electrolyte, proving the very narrow spectrum of options that organic electrosynthesis is restricted to at this point of its short life. In all the aforementioned cases, products were isolated by means of column chromatography, and no comment on the reuse of solvents or electrolytes is mentioned by authors. Therefore, despite its clear advantages, electrosynthesis still faces several operational challenges that limit its full potential as a sustainable approach. Thus, our aim was to study the use of a new sustainable reaction media/electrolyte system capable of being recycled in the shape of DESs to pave the way for new possibilities in organic electrosynthesis.

## 2 Results and Discussion

With this objective in mind, and guided by literature precedents, the model reaction between *p*‐methylbenzenesulfinate (**1a**) and quinoline *N*‐oxide (**2a**) was selected for optimization. An H‐type electrochemical cell was chosen, as it serves a dual purpose: preventing undesired side reactions in the anodic compartment (where the organic transformation occurs in a DES medium) and allowing the use of an aqueous NaCl (0.1 M) solution in the cathodic compartment for hydrogen gas generation while closing the electric circuit. To our delight, after testing some choline chloride‐based DES with moderate results (Table S1, Supporting Information), it was found that the mixture tetrabutylammonium chloride:ethylene glycol (TBAC:EG, 1:3)^[^ ^31^ ^]^ afforded the desired product in 56% yield after 2 h at 10 mA using carbon felt as anode and a platinum wire as cathode (**Table** **1**, entry 1).

**Table 1:** Selected examples of the optimization.

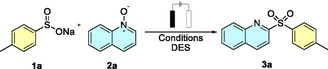					
Entrya)	DES (molar ratio)	I [mA]	Equiv. **1a**	t [h]	Yield **3a** [%]b)
1	TBAC:EG (1:3)	10	2.5	2	56
2	TBAB:EG (1:3)	10	2.5	2	58
3	TBAB:EG (1:3)	10	2.5	16	66
4	TBAB:EG (1:3)	15	2.5	2	85
5	TBAB:EG (1:3)	20	2.5	2	85
6	TBAB:EG (1:3)	2.5	2.5	72	45
7	TBAB:EG (1:3)	0	2.5	2	n.d.
8	TBAB:EG (1:3)	15	2.5	2	n.dc)
9	TBAB:EG (1:3)	15	2.5	2	85d)
10	TBAB:EG (1:3)	15	4.0	2	89
11	TBAB:EG (1:3)	15	1.0	2	12

a) Reaction performed in a divided cell using DES in the anodic compartment and water with sodium chloride (0.1 M) in the cathodic compartment;

b) Yields determined by ^1^H NMR using 1,2,4,5‐tetramethylbenzene as internal standard;

c) Reaction performed in an undivided cell;

d) Reaction carried out at 60 °C.

Interestingly, exchanging the salt counterion from chloride to bromide did not affect the reaction outcome, and therefore the more affordable TBAB was chosen over its chlorinated counterpart (entry 2). Extending the reaction time did not result in a substantial improvement in the reaction yield, but instead setting the current at 15 mA did in fact improve results to 85% yield of **3a**, although higher currents could not improve the yield further (entries 3–5), probably due to competing reactions like desulfonylation,^[^ ^32^ ^]^ or DES degradation (see Figure S1 and S2, Supporting Information). Thus, a lower current with an extended reaction time was also tested, but poor yield was obtained (entry 6). It was also proven that the reaction did not proceed at all without current or in an undivided cell (entries 7–8). Finally, increasing the temperature (entry 9) or the sulfinate equivalents to 4.0 only led to a minimal improvement in the reaction outcome, which was considered not worthy, while using only 1.0 equivalent afforded a marginal yield (entries 10–11), which is in accordance with the expected reaction mechanism requiring two equivalents of sulfinate to achieve the final rearomatization.

**Scheme 1:**
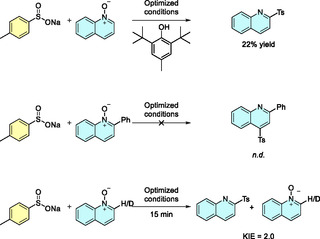
Control experiments performed to explore the reaction mechanism.

**Figure 2:**
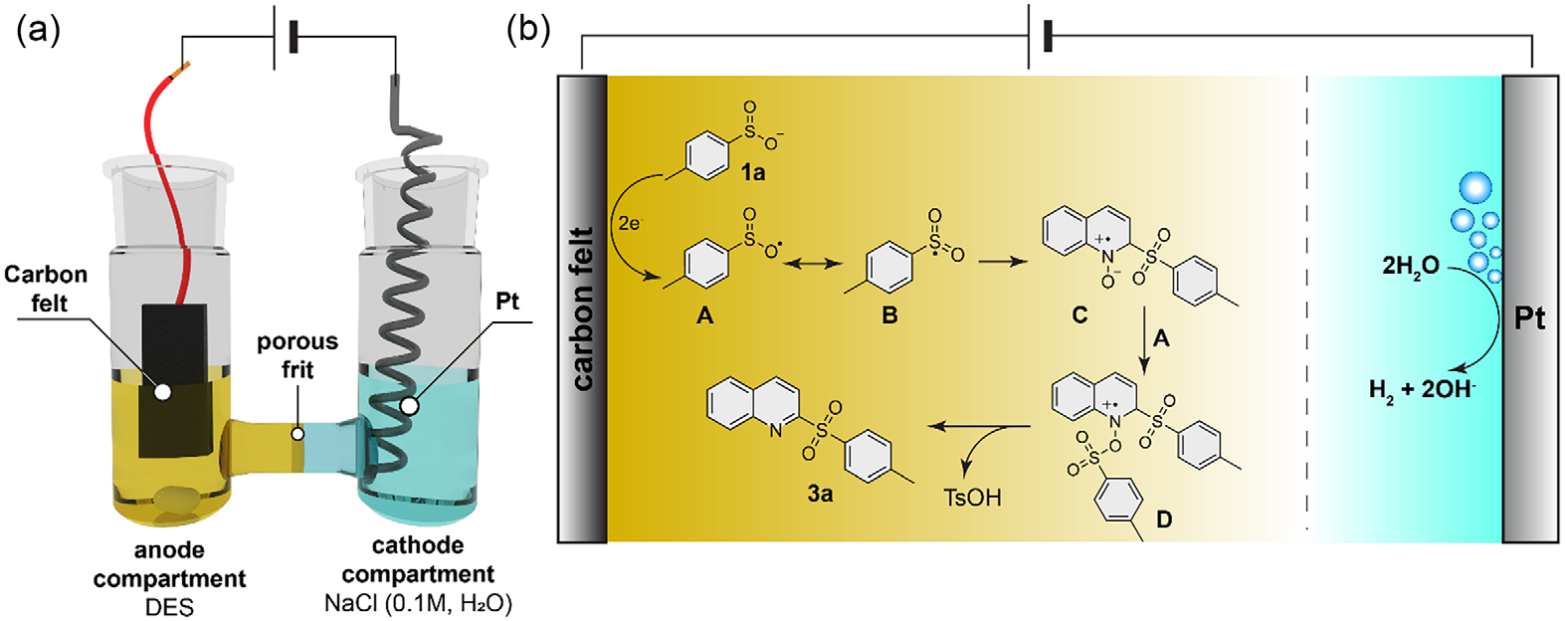
a) Scheme of the H‐type cell and the components employed to carry out the electrochemical tests. b) Proposed reaction mechanism.

To gain deeper insight into the reaction mechanism and the role of the DES, a series of mechanistic experiments was conducted (**Scheme** **1**). First, cyclic voltammetries of the DES with the sulfinate **1a** were performed (see Supporting Information), confirming that the oxidation of the starting material occurred within the stability window of the DES. Then, given the radical nature of the transformation, the reaction was carried out in the presence of 3,5‐di‐*tert*‐4‐butylhydroxytoluene as a radical scavenger under otherwise optimized conditions. A significant decrease in reaction yield was observed (**Figure** **2**a), supporting the involvement of radical intermediates. When a 2‐phenyl‐substituted quinoline *N*‐oxide was used as the substrate, no product formation was detected, confirming the complete regioselectivity of the reaction and ruling out reactivity at the 4‐position of the quinoline scaffold. Finally, a 1:1 mixture of quinoline *N*‐oxide **2a** and its deuterated analog **2a–D** was subjected to the reaction, which was quenched after only 15 min. Analysis of the crude mixture revealed that the reaction rate of the nondeuterated compound was approximately twice that of the deuterated one, corresponding to a kinetic isotope effect of 2.0. This result suggests that the rearomatization step may be rate limiting in the overall transformation.

According to literature precedents,^[^ ^30^ ^]^ this step proceeds *via* a second equivalent of sulfinate, which is oxidized to the corresponding radical and subsequently traps the *N*‐centered radical intermediate formed after the initial sulfinate radical addition (**C**). This is followed by the elimination of the corresponding sulfonic acid, restoring aromaticity and yielding the final product.^[^ ^30^ ^]^ This proposed mechanism is consistent with our observations: when the reaction was carried out with only one equivalent of sulfinate, only marginal yields were obtained (Table 1, entry 9). With these observations, a reaction mechanism was proposed (Figure 2 and S3, Supporting Information).

With the optimized conditions in hand and preliminary mechanistic insights established, we next investigated the reaction scope. Quinoline *N*‐oxide (**2a**) was first reacted with a series of aryl sulfinates bearing electron‐donating (**Table** **2** and **3a**), neutral (**3b**–**3d**), and electron‐withdrawing substituents (**3e**–**3i**). The reaction proceeded smoothly across this range, delivering the corresponding sulfonylated products in good to excellent yields, including compound **3g**, an intermediate^[^ ^33^ ^]^ in the synthesis of anticancer drug **MPT0G236**. Notably, both heteroaromatic (**3i**) and aliphatic sulfinates (**3j**–**3k**) and different functionalities were well tolerated, further demonstrating the versatility of the method. Only two limitations were found over the explored sulfinates, namely, sodium trifluoromethylsulfinate and sodium benzo[*d*]thiazole‐2‐sulfinate, compounds known for their ability to release SO_2_.^[^ ^34^, ^35^ ^]^

**Table 2:** Scope of the reaction. (Reactions were performed on an H‐type cell; anodic compartment composed of 10 mL of DES, sodium sulfinate (0.625 mmol), quinoline *N*‐oxide (0.25 mmol), (molar ratio **1a/2a** = 2.5); cathodic compartment was filled with 10 mL of 0.1 M NaCl aqueous solution, using carbon felt 1.5 cm2 as the working electrode and platinum wire as the counterelectrode their respective compartments at 15 mA for 2 h. All yields expressed are isolated yields.)

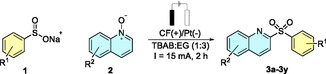
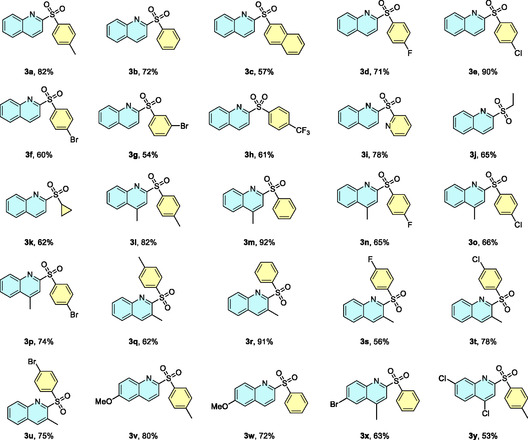

The effects of the substituents on the quinoline ring were also explored. Reactions with 4‐methylquinoline *N*‐oxide yielded products (**3l**–**3p**) in comparable yields to the parent substrate, while 3‐methylquinoline *N*‐oxide generally afforded slightly lower yields, likely due to increased steric hindrance. However, product yields varied depending on the specific substrate used. Finally, we examined the influence of different substituents on the quinoline *N*‐oxide. Electron‐rich derivatives (**3v**–**3w**) seem to afford higher yields than their halogenated counterparts (**3x**–**3y**), suggesting a modest beneficial influence of electron‐donating groups. Overall, however, the differences were not pronounced, and the isolated yields likely reflect, at least in part, variations in purification efficiency arising from differences in substrate solubility and polarity.

**Figure 3:**
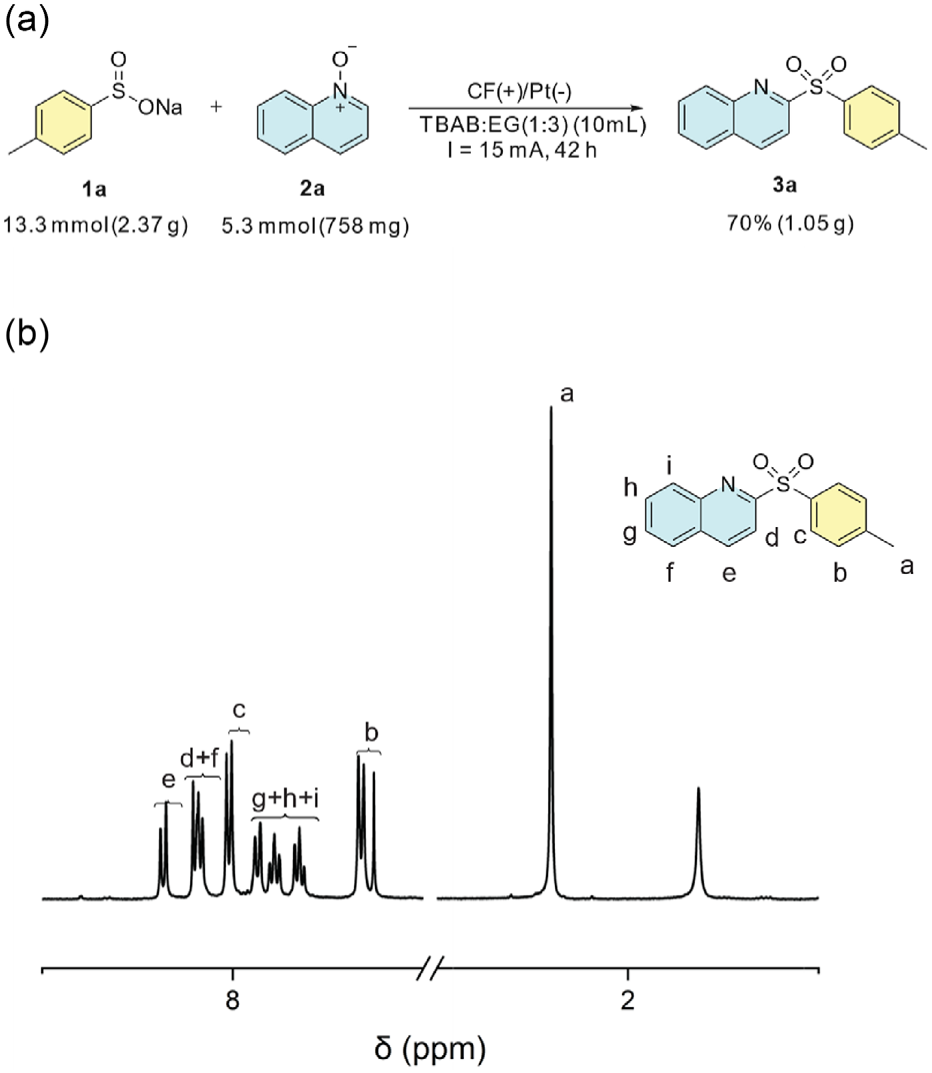
a) Gram‐scale reaction. b) ^1^H NMR spectrum of product **3a** after precipitation with water and filtration without any further purification step.

Although most reactions were conducted on a 1 mmol scale, a gram‐scale experiment was performed under identical conditions, maintaining a current of 15 mA and extending the reaction time from 2 to 42 h without using any type of volatile organic compound (VOC) solvent in the whole process (**Figure** **3**a). This afforded 1.05 g of product **3a** in 70% isolated yield by means of simple precipitation and filtration after water addition to the crude mixture, demonstrating the scalability of the process without significant drawbacks in the overall yield.

This method offers notable advantages in terms of sustainability and ease of purification. Due to the water solubility of most reaction components, including the DES, excess sulfinate, and unreacted quinoline *N*‐oxide, the reaction mixture contained at the anodic compartment can be treated with water after the reaction. In this way, the sulfone product precipitates and can be readily isolated with high purity by simple filtration and water rinsing (Figure 3b). Thus, both the synthesis and purification are performed entirely without organic volatile solvents, representing a sustainable and practical alternative to conventional methods relying on VOCs.

**Figure 4:**
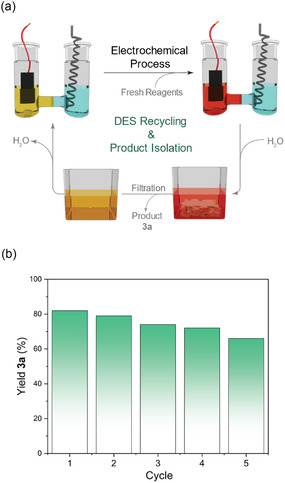
a) Schematic representation of the step‐by‐step recycling process, depicting a full cycle of the process. b) Recyclability study performed by monitoring the yield of 3a after successive recovery cycles of DES.

A common drawback of many recent electrosynthetic protocols is the requirement for large amounts of nonrecyclable supporting electrolytes. To address this limitation, we evaluated the recyclability of the DES‐based solvent/electrolyte system. Following the reaction, water was added to the mixture, and the sulfone product **3a** was isolated by filtration as a solid. The aqueous filtrate was then concentrated under reduced pressure to recover the DES, which was reused in subsequent reactions. This recycling process was repeated over five consecutive cycles (**Figure** **4**), with only a slight decrease in reaction yield observed. The gradual decline in efficiency can be tentatively attributed to three main factors: 1) the accumulation of salts and byproducts in the reaction mixture, and 2) partial oxidation of ethylene glycol during electrolysis (see Supporting Information for further details), and 3) the loss of DES during the recycling process.

**Figure 5:**
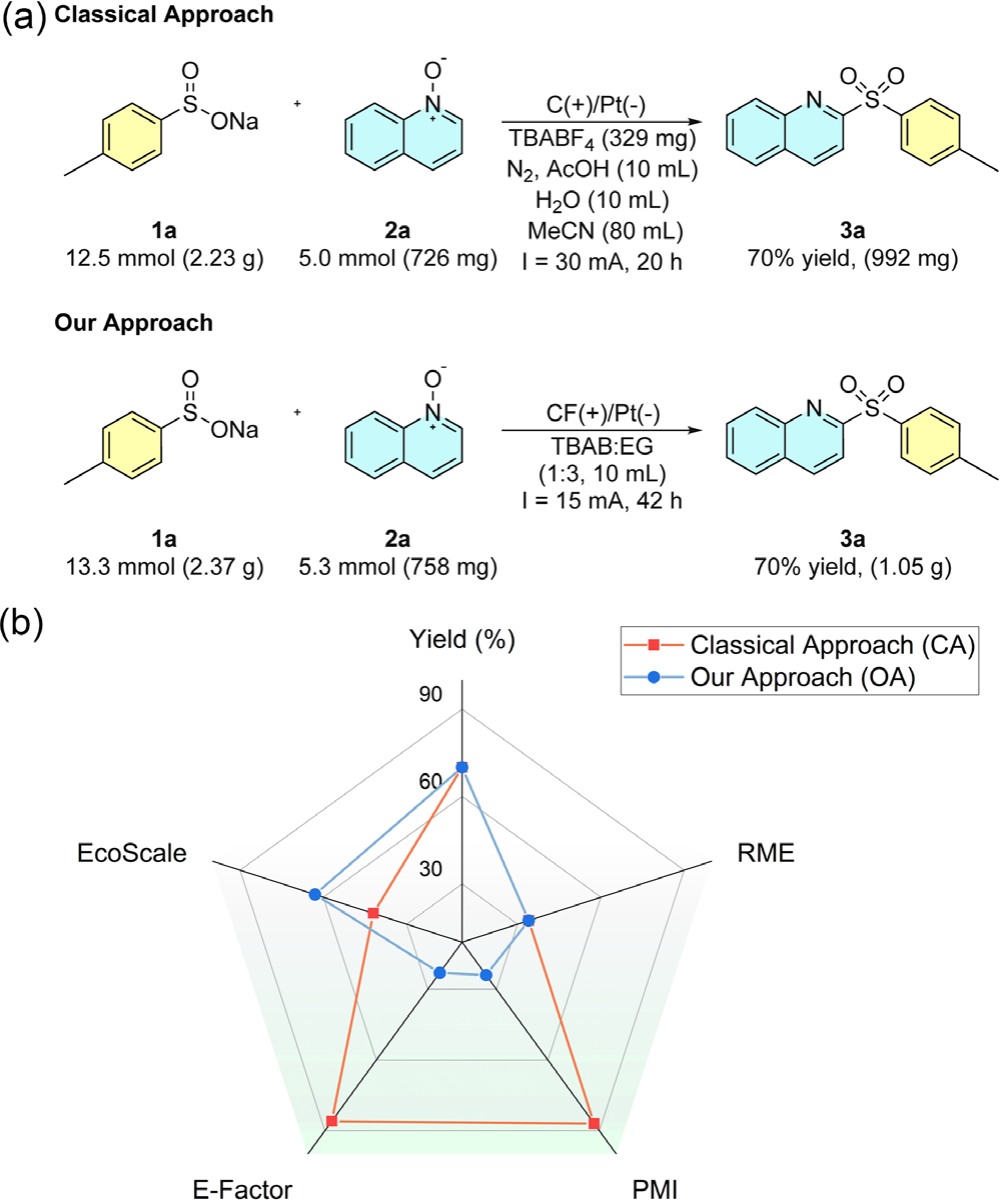
a) Gram‐scale reaction conditions for CA^[^ ^30^ ^]^ and our procedure. b) Comparison of green metrics of the present work and CA^[^ ^30^ ^]^ for the gram‐scale synthesis of compound **3a**.

To quantitatively assess the “greenness” of our methodology, we evaluated several green chemistry metrics, including yield, reaction mass efficiency (RME), process mass intensity (PMI) or E‐factor, as well as qualitative indicators such as EcoScale.^[^ ^36^ ^]^ To enable a meaningful comparison with literature reports, we determined these metrics for both our optimized approach and a representative classical approach (CA),^[^ ^30^ ^]^ as summarized in **Figure** **5**a, without taking into account the recyclability of our system (see more details in Supporting Information). In both cases, a gram‐scale reaction between sodium toluenesulfinate (**1a**) and quinoline *N*‐oxide (**2a**) was performed and used to compute the relevant metrics. The conventional approach (CA) requires a nitrogen atmosphere and employs acetonitrile and acetic acid as solvents in a diluted system. Additionally, silica gel and mixtures of ethyl acetate and hexane are used during the purification step, and the electrolyte cannot be reused. In contrast, our method is conducted under air in a DES mixture, with water as the only solvent required for purification. A summary of the comparative results is presented in Figure 5b (see the Supporting Information for further details). Both approaches deliver the same reaction yield (70%) and use the same equivalents of sulfinate, resulting in identical RME values.

However, these metrics do not account for the amount of solvent used. In contrast, E‐factor or PMI metrics do take into account the solvents employed and consequently, our method achieves significantly lower values in these two aspects. It is important to note that the purification process in the CA was not considered due to insufficient information about the chromatographic step. In our case, simple addition of water followed by filtration suffices to recover the product. In contrast, the CA requires silica gel, hexane, and ethyl acetate, which would substantially increase the environmental impact. Moreover, these calculations only consider the mass of reagents and solvents, without evaluating their environmental friendliness. The EcoScale metric, however, takes into account the availability and toxicity of each component, as well as the purification method. According to EcoScale, the CA scores 42 points, classified as “inadequate,” while our approach scores 63 points, considered “acceptable.”^[^ ^36^ ^]^

## 3 Conclusion

A sustainable and efficient electrosynthetic method for the preparation of 2‐arylsulfinylquinolines has been developed using a deep eutectic solvent (DES) as both reaction medium and supporting electrolyte, being one of the first reports of an electrosynthetic procedure in this sustainable reaction media. The protocol proceeds under mild conditions in a divided electrochemical cell, affording high yields across a broad substrate scope. The method is scalable and allows for product isolation by simple filtration and water washing, eliminating the need for volatile organic solvents or chromatographic purification. Even though DESs’ high viscosity and possible electrode interaction can affect the outcome of an electrosynthetic protocol, in this case the DES employed can be recovered and reused over multiple cycles with only a minor decrease in yield, attributed to the accumulation of by‐products and partial degradation of the solvent components. Cyclic voltammetry studies indicate that the DES facilitates the oxidation of the sulfinate salt, supporting its dual role as solvent and electrolyte. Green chemistry metrics, including process mass intensity (PMI), reaction mass efficiency (RME), and the EcoScale, demonstrate that the present approach outperforms conventional methods, which typically rely on non‐recyclable electrolytes and organic solvents. The complete avoidance of volatile organic compounds in both the reaction and purification steps, combined with solvent recyclability, positions this protocol as a promising and sustainable alternative for the development of electrochemical transformations in organic synthesis.

## 4 Experimental Section

4.1

4.1.1

##### Instrumentation

^1^ H NMR (400 MHz) spectra were recorded on *Bruker AC‐400* NMR spectrometers respectively in proton‐coupled mode. ^13^C NMR (101 MHz) spectra were recorded *on Bruker AC‐400* NMR spectrometers respectively in proton decoupled mode at 20 °C; chemical shifts are given in *δ* (parts per million) and coupling constants (*J*) in Hertz. Low‐resolution mass spectra (EI) were obtained at 70 eV on an Agilent Technologies GC‐8890 N equipped with 5977B GC/MSD detector giving fragment ions in *m/z* with relative intensities (%) in parentheses. Gas chromatography–mass spectrometry (GC‐MS) chromatograph was equipped with an Agilent Technologies HP‐5MS Ultra Inert column (30 m × 0.250 mm × 0.25 μm). High‐resolution mass spectra (EI) were recorded at 70 eV on an Agilent 7200 flight (Q‐TOF) spectrometer with a Direct Insertion Probe (73DIP‐1). Ions derived from breaks are given as *m/z* with relative percent intensities in brackets.Infrared spectra were measured on a *Jasco FT/IR‐4100* Fourier Transform Infrared spectrometer. Thin layer chromatography (TLC) was carried out on Schleicher & Schuell F1400/LS 254 plates coated with a 0.2 mm layer of silica gel; detection by UV_254_ light. Column chromatography was performed using silica gel 60 of 40–63 mesh. Electrosynthetic reactions were carried out on a *IKA Electrasyn 2.0*. Cyclic Voltammetries were recorded using a *Bio‐Logic SP‐200* using a three‐electrode set up with a Ag/AgCl reference electrode.

##### Procedure for the Electrosynthesis of Sulfones

The electrocatalyzed sulfonylation reactions performed in this work were carried out in a divided H‐type electrochemical cell. For this purpose, 10 mL of the eutectic mixture were added into the anodic compartment, along with the required amounts of sodium sulfinate (0.625 mmol) and quinoline *N*‐oxide (0.25 mmol), (molar ratio **1a**/**2a** = 2.5). The cathodic compartment was filled with 10 mL of a 0.1 M NaCl aqueous solution. The mixture was stirred, and the corresponding electrodes (carbon felt 1.5 cm^2^ as working and platinum wire as counter) were placed in their respective compartments. These electrodes were connected to an *IKA ElectraSyn* 2.0 device, which supplied a constant current of 15 mA and stirring at 1200 rpm for 2 h.

After the reaction time (2 h), the anodic compartment was diluted with water, obtaining the corresponding sulfone product **3** as a precipitate, which was filtered off and washed with water. If needed, crude mixtures could be further purified by column chromatography using mixtures of hexanes and ethyl acetate as eluent.

##### Procedure for Recycling Experiments

For the recycling experiments, the reaction between sulfinate **1a** (0.625 mmol) and quinoline *N*‐oxide **2a** (0.25 mmol) was carried out following procedure for the electrosynthesis of sulfones. Upon completion of the reaction, water (25 mL) was added to the anodic compartment, causing precipitation of the product **3a**, which was then filtered and rinsed with water (225 mL). The resulting filtrate was concentrated under reduced pressure to remove water and reused in a new cycle with fresh reagents [**1a** (0.625 mmol) and **2a** (0.25 mmol)], without the addition of any extra DES.

##### Procedure for Gram‐Scale Reaction

Gram scale reaction was performed as described in the procedure for the electrosynthesis of sulfones in higher concentration under extended reaction time. Briefly, 10 mL of the eutectic mixture were added into the anodic compartment, along with the required amounts of sodium sulfinate (13.3 mmol) and quinoline *N*‐oxide (5.3 mmol). The cathodic compartment was filled with 10 mL of a 0.1 M NaCl aqueous solution. The mixture was stirred, and the corresponding electrodes (carbon felt 1.5 cm^2^ as working and platinum wire as counter) were placed in their respective compartments. These electrodes were connected to an *IKA ElectraSyn* 2.0 device, which supplied a constant current of 15 mA and stirring at 1200 rpm for 42 h.

## Supporting Information

The authors have cited additional references within the Supporting Information.^[37–49]^

## Conflict of Interest

The authors declare no conflict of interest.

## Supporting information

Supplementary Material

## Data Availability

The data that support the findings of this study are available in the supplementary material of this article.
