# Supplementary material for: Electrochemical Sulfonylation in Deep Eutectic Solvents Enables the Sustainable Synthesis of 2‐Quinoline Sulfones
Source: ChemSusChem. 2025 Oct 20;18(24):e202501779. doi: 10.1002/cssc.202501779 (PMC12703430; doi:10.1002/cssc.202501779)

Supporting Information

Electrochemical Sulfonylation in Deep Eutectic Solvents Enables the Sustainable Synthesis of 2-Quinoline Sulfones

Darío Adsuar,[a, b]+ Xavier Marset,*[a]+ Diego J. Ramón,*[b] and Néstor Guijarro *[a]

[a] Instituto de Electroquímica, Universidad de Alicante, Apdo. 99, Alicante, Spain
[b] Instituto de Síntesis Orgánica and Departamento de Química Orgánica, Universidad de Alicante, Apdo. 99, Alicante, Spain.

[+] These authors contributed equally to this work.

e-mail: xavier.marset@ua.es, djramon@ua.es, nestor.guijarro@ua.es

Contents

Materials and Methods **3**

Chemicals and Materials **3**

Instrumentation. **3**

Optimization of the reaction conditions. **4**

Electrochemical characterization of DES. **5**

General Procedures **8**

General procedure A for the synthesis of sulfinates (1). **8**

General procedure B for the synthesis of quinoline *N*-oxides (2) **8**

General procedure C for the electrosynthesis of sulfones (3) **8**

Procedure for DESs preparation **9**

Procedure for recycling experiments **9**

Procedure for gram-scale reaction **9**

Characterization Data **10**

Green Metrics calculations. **19**

Yield 19

RME 19

PMI 19

E-Factor 19

Ecoscale 20

References **23**

Materials and Methods

Chemicals and Materials

All commercial chemicals were analytical reagents and were used as received without further purification. Carbon Felt electrodes (3.18 mm thick, 99.0%) were purchased from *Thermo Scientific*.

Instrumentation

^{1}H NMR (400 MHz) spectra were recorded on *Bruker AC-400* NMR spectrometers respectively in proton-coupled mode. ^{13}C NMR (101 MHz) spectra were recorded *on Bruker AC-400* NMR spectrometers respectively in proton decoupled mode at 20 °C; chemical shifts are given in *δ* (parts per million) and coupling constants (*J*) in Hertz.

Low-resolution mass spectra (EI) were obtained at 70 eV on an Agilent Technologies GC-8890N equipped with 5977B GC/MSD detector giving fragment ions in m/z with relative intensities (%) in parentheses. GC-MS chromatograph was equipped with an Agilent Technologies HP-5MS Ultra Inert column (30 m x 0.250 mm x 0.25 µm).

High-resolution mass spectra (EI) were recorded at 70 eV on an Agilent 7200 flight (Q-TOF) spectrometer with a Direct Insertion Probe (73DIP-1). Ions derived from breaks are given as *m/z* with relative percent intensities in brackets.

Infrared spectra were measured on *a Jasco FT/IR-4100* Fourier Transform Infrared pectometer.

Thin layer chromatography (TLC) was carried out on Schleicher & Schuell F1400/LS 254 plates coated with a 0.2 mm layer of silica gel; detection by UV_{254} light. Column chromatography was performed using silica gel 60 of 40-63 mesh.

Electrosynthetic reactions were carried out on a *IKA Electrasyn 2.0*. Cyclic Voltammetries were recorded on a *Bio-Logic SAS SP-200* using a three-electrode set up.

Optimization of the reaction conditions.

Table S1. Optimization of reaction conditions.

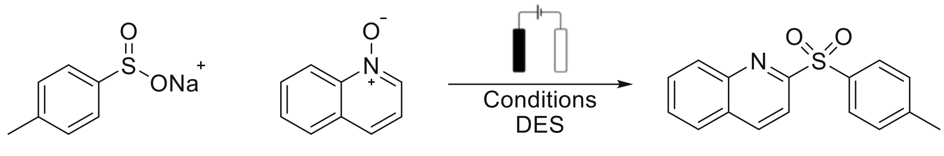

Ent.	DES (molar ratio)	I (mA)	Electrodes	t (h)	Equiv 1a	Yield 3a (%)[b]
1	LiTFSI:EG (1:4)[b]	10	CF(+)/Pt(-)	2	2.5	n.d.
2	$MgCl_2 \cdot 6H_2O$:$MeCONH_2$:urea (1:1:7)	10	CF(+)/Pt(-)	2	2.5	n.d.
3	ChCl:$MgCl_2 \cdot 6H_2O$:LEA (1:1:2)[c]	10	CF(+)/Pt(-)	2	2.5	26
4	ChCl:HFIP (1:2)	10	CF(+)/Pt(-)	2	2.5	46
5	ChCl:LA (1:2)[d]	10	CF(+)/Pt(-)	2	2.5	19
6	TBACl:EG (1:3)	10	CF(+)/Pt(-)	2	2.5	56
7	$TBAPF_6$:HFIP (1:2)	10	CF(+)/Pt(-)	2	2.5	11
8	$TBABF_4$:HFIP (1:2)	10	CF(+)/Pt(-)	2	2.5	28
9	ChCl:EG (1:2)	10	CF(+)/Pt(-)	2	2.5	7
10	AcChCl:EG (1:2)	10	CF(+)/Pt(-)	2	2.5	23
11	$TBAClO_4$:HFIP (1:2)	10	CF(+)/Pt(-)	2	2.5	20
12	TBAB:EG (1:3)	10	CF(+)/Pt(-)	2	2.5	58
13	TBAB:EG (1:3)	10	CF(+)/Pt(-)	4	2.5	74
14	TBAB:EG (1:3)	10	CF(+)/Pt(-)	8	2.5	66
15	TBAB:EG (1:3)	10	CF(+)/Pt(-)	16	2.5	66
16	TBAB:EG (1:3)	15	CF(+)/Pt(-)	2	2.5	85
17	TBAB:EG (1:3)	15	CF(+)/Pt(-)	4	2.5	84
18	TBAB:EG (1:3)	20	CF(+)/Pt(-)	2	2.5	85
19	TBAB:EG (1:3)	20	CF(+)/Pt(-)	4	2.5	83
20	TBAB:EG (1:3)	2.5	CF(+)/Pt(-)	72	2.5	45
21	TBAB:EG (1:3)	0	CF(+)/Pt(-)	2	2.5	n.d.
22	TBAB:EG (1:3)	15	CF(+)/Ni(-)	2	2.5	48
23	TBAB:EG (1:3)	15	CF(+)/Pt(-)	2	4	89
24	TBAB:EG (1:3)	15	CF(+)/Pt(-)	2	1	12
25	TBAB:EG (1:3)	15	CF(+)/Pt(-)	2	2.5	85[e]
26	TBAB:EG (1:3)	15	CF(+)/Pt(-)	2	2.5	n.d[f]

[a]Yield determined by GC using 1,2,4,5-tetramethylbenzene as internal. [b] LiTFSI stands for Lithium bis(trifluoromethanesulfonyl)imide. [c] LEA stands for levulinic acid. [d] LA stands for L-lactic acid. [e] Reaction performed at 60 ºC. [f] Reaction performed in an undivided cell

Electrochemical characterization of DES.

The eutectic mixture TBAB:EG (1:3) remains relatively stable within a working potential range of -2 to 0.6 V. When sodium 4-methylbenzenesulfinate was added to the reaction medium, a significant increase in the current applied to the system was observed (Figure S1). This increase begins at a potential around 0.4 V, indicating that the compound starts to oxidize at this potential. As can be seen, the oxidation of sodium 4-methylbenzenesulfinate occurs at a lower potential than the degradation of the TBAB:EG (1:3) eutectic mixture. This explains the high efficiency of this DES as a reaction medium in the process and the excellent yields obtained in this study, as the desired oxidation reaction takes place before the onset of eutectic degradation.

^{1}H NMR analyses of the DES were carried out on the freshly prepared mixture (Figure S2-a) and after 48 hours of electrolysis in the absence of any reagent (Figure S2-b). After electrolysis, the ethylene glycol signals appear shifted and broadened, while the signals corresponding to tetrabutylammonium remain unchanged. These results confirm that ethylene glycol undergoes oxidation under the reaction conditions once the sulfinate starting material is depleted, which negatively impacts the recyclability of the DES during prolonged electrolysis.

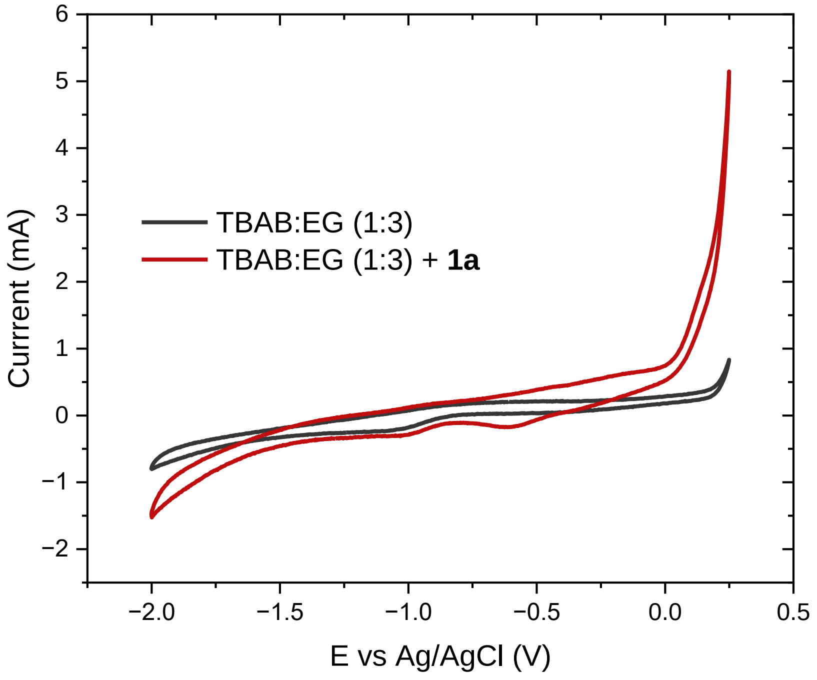

Figure S1. Cyclic voltammograms performed using a three-electrode set up: carbon felt (working electrode), Ag/AgCl (reference electrode) and platinum (counter electrode).

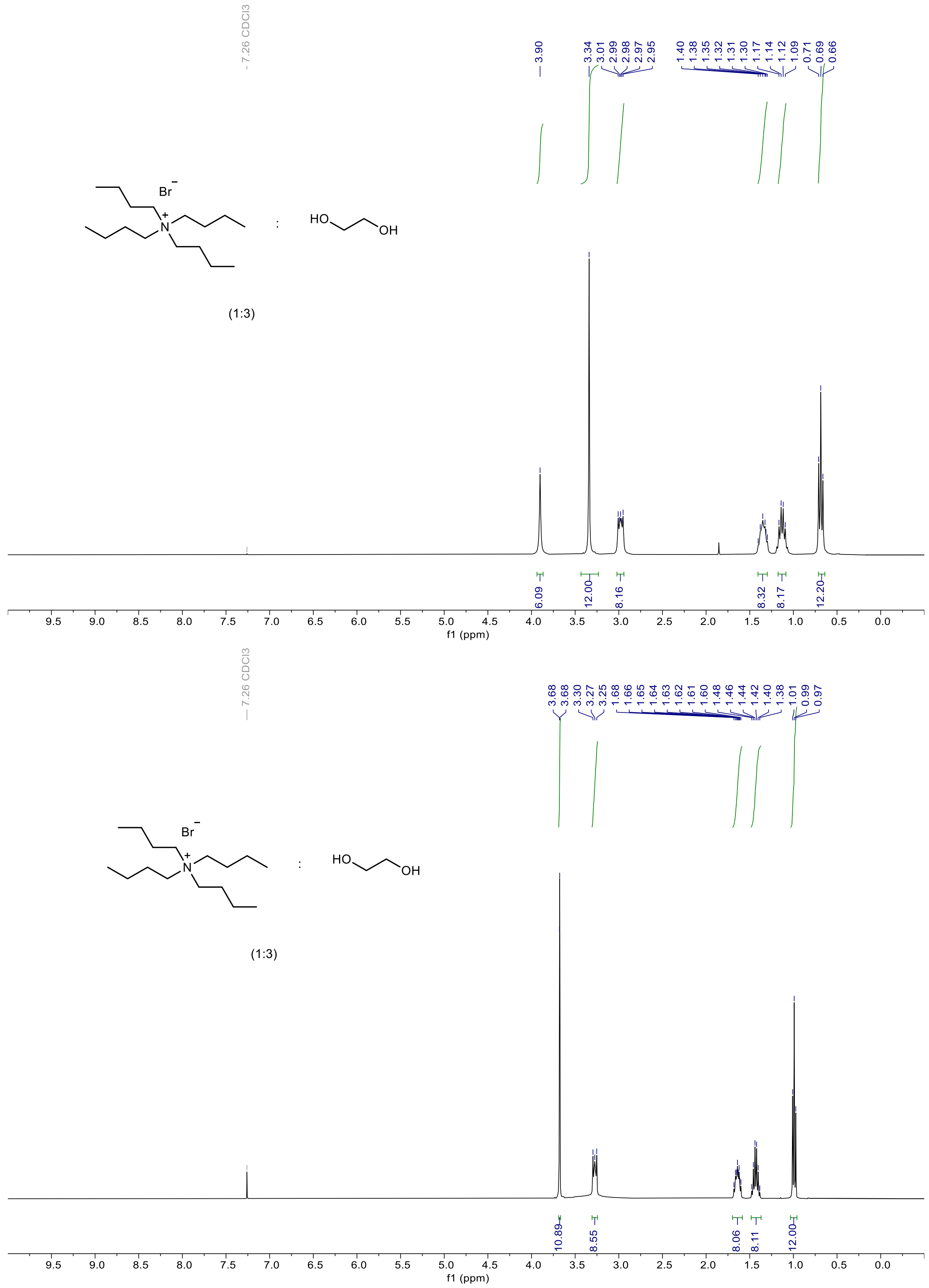

Figure S2. ^{1}H NMR spectra of DES composed by TBABr:EG (1:3) a) freshly prepared, b) after 48 h of electrolysis.

Proposed reaction mechanism

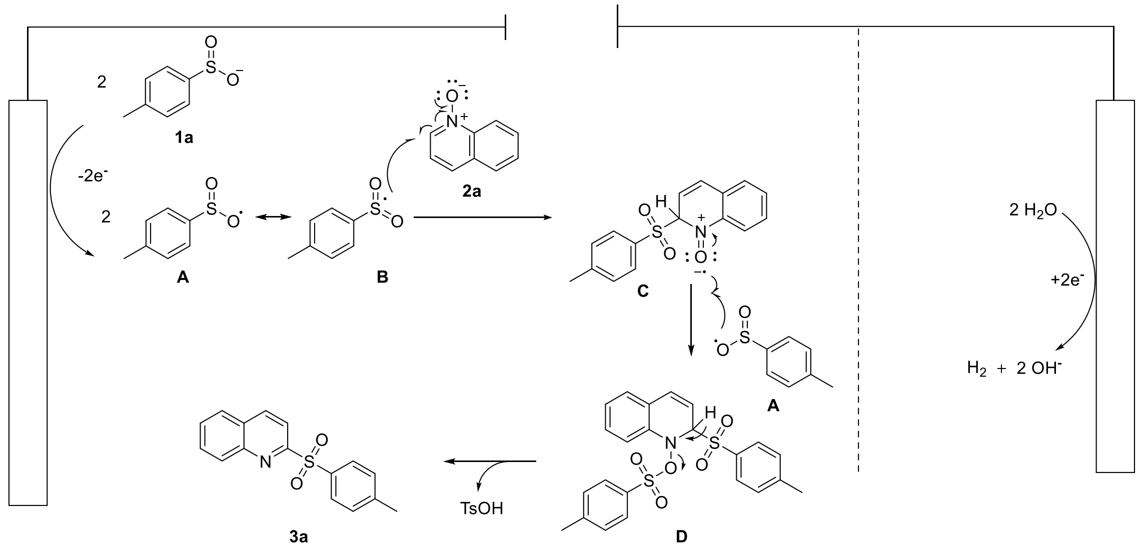

Figure S3. Proposed reaction mechanism

General Procedures

General procedure A for the synthesis of sulfinates (1).

The procedure was adapted from a literature precedent.[1] The corresponding sulfonyl chloride (5.00 mmol) was added to an aqueous solution of sodium sulfite (1.26 g, 10.0 mmol) and sodium bicarbonate (840 mg, 10.0 mmol) in 5 mL of water (1 M). The reaction mixture was heated at 80 °C for 3 hours. After cooling to room temperature, the volatiles were removed under reduced pressure. The resulting solid was thoroughly washed with ethanol, and the combined ethanol washes were evaporated under vacuum to afford the desired sulfinate as a solid.

General procedure B for the synthesis of quinoline *N*-oxides (2)

Quinoline *N*-oxides were synthesized adapting a literature precedent.[2] Briefly, quinoline (7.0 mmol, 1.0 equiv.) was dissolved in 40 mL of dichloromethane (DCM). Then, meta-chloroperoxybenzoic acid (*m*CPBA, 10.5 mmol, 1.5 equivalents) was added slowly at 0 °C using an ice-water bath. The reaction mixture was then stirred at room temperature for 16 hours. After this period, a liquid-liquid extraction was performed using saturated aqueous solutions of NaCl and $NaHCO_3$. Finally, the crude product was purified by column chromatography using a mixture of ethyl acetate and methanol (4:1, v/v) as the eluent.

General procedure C for the electrosynthesis of sulfones (3)

The electrocatalyzed sulfonylation reactions performed in this work were carried out in a divided H-type electrochemical cell. For this purpose, 10 mL of the eutectic mixture were added into the anodic compartment, along with the required amounts of sodium sulfinate (0.625 mmol) and quinoline *N*-oxide (0.25 mmol), (molar ratio **1a**/**2a** = 2.5). The cathodic compartment was filled with 10 mL of a 0.1 M NaCl aqueous solution. The mixture was stirred, and the corresponding electrodes (carbon felt 1.5 cm^2 as working and platinum wire as counter) were placed in their respective compartments. These electrodes were connected to an *IKA ElectraSyn* 2.0 device, which supplied a constant current of 15 mA and stirring at 1200 rpm for 2 hours.

After the reaction time (2 h), the anodic compartment was diluted with water (25 mL), obtaining the corresponding sulfone product **3** as a precipitate, which was filtered off and washed with water (225 mL). If needed, crude mixtures could be further purified by column chromatography using mixtures of hexanes and ethyl acetate as eluent.

Procedure for DESs preparation

The eutectic mixtures used in this study were prepared in round-bottom flasks, where the DES components were combined in the specified molar ratios. Magnetic stirring was then applied, and the mixture was heated to approximately 80 °C for the time required to obtain a homogeneous liquid phase (about 20 min). In cases where a hygroscopic component was used, an inert atmosphere was created inside the flask by introducing argon gas. This precaution prevents atmospheric moisture from affecting the composition of the DES during its preparation.

Procedure for recycling experiments

For the recycling experiments, the reaction between sulfinate **1a** and quinoline *N*-oxide **2a** was carried out following General Procedure C. Upon completion of the reaction, water (25 mL) was added to the anodic compartment, causing precipitation of the product **3a**, which was then filtered and rinsed with water (225 mL). The resulting filtrate was concentrated under reduced pressure to remove water and reused in a new cycle with fresh reagents **1a** (0.625 mmol) and **2a** (0.25 mmol), without the addition of any extra DES.

Procedure for gram-scale reaction

Gram scale reaction was performed as described in general procedure C in higher concentration under extended reaction time. Briefly, 10 mL of the eutectic mixture were added into the anodic compartment, along with the required amounts of sodium sulfinate (13.3 mmol) and quinoline *N*-oxide (5.3 mmol). The cathodic compartment was filled with 10 mL of a 0.1 M NaCl aqueous solution. The mixture was stirred, and the corresponding electrodes (carbon felt 1.5 cm^2 as working and platinum wire as counter) were placed in their respective compartments. These electrodes were connected to an *IKA ElectraSyn* 2.0 device, which supplied a constant current of 15 mA and stirring at 1200 rpm for 42 hours. Reaction was quenched by addition of water (25 mL) obtaining a precipitate, which was filtered under vacuum and rinsed with water (225 mL) to obtain product **3a**.

Characterization Data

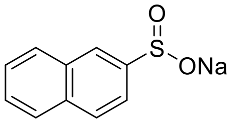

Sodium naphthalene-2-sulfinate (1c). Obtained following procedure A. White solid, 542 mg, 82% isolated yield. Spectroscopic data matched with previously reported on literature.[3] R_f = 0.34 (EtOAc:MeOH/4:1). ^{1}H NMR (400 MHz, D_2O): δ = 8.19 (d, *J* = 1.7 Hz, 1H), 8.14 – 8.10 (m, 2H), 8.05 (dd, *J* = 6.1, 3.4 Hz, 1H), 7.83 (dd, *J* = 8.6, 1.7 Hz, 1H), 7.69 (dd, *J* = 6.3, 3.3 Hz, 2H). ^{13}C NMR (101 MHz, MeOD): δ = 154.5, 135.5, 134.5, 129.6, 129.5, 128.8, 127.7, 127.4, 124.6, 122.5. IR (cm^{-1}): $\tilde{\nu}$ = 1219, 1026, 944, 820, 740, 632.

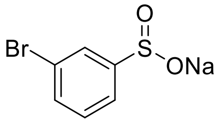

Sodium 3-bromobenzenesulfinate (1g). Obtained following procedure A. White solid, 568 mg, 78% isolated yield. Spectroscopic data matched with previously reported on literature.[4] R_f = 0.31 (EtOAc:MeOH/4:1). ^{1}H NMR (400 MHz, D_2O): δ = 7.83 (t, *J* = 1.7 Hz, 1H), 7.71 (ddd, *J* = 7.9, 2.0, 1.1 Hz, 1H), 7.64 (dt, *J* = 7.7, 1.3 Hz, 1H), 7.48 (t, *J* = 7.8 Hz, 1H). ^{13}C NMR (101 MHz, MeOD): δ = 160.0, 133.3, 131.4, 128.2, 124.3, 123.6. IR (cm^{-1}): $\tilde{\nu}$ = 1103, 1016, 953, 787, 679.

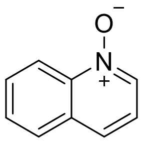

Quinoline 1-oxide (2a). Obtained following procedure B. White solid, 410 mg, 40% isolated yield. Spectroscopic data matched with previously reported on literature.[5] R_f = 0.30 (EtOAc:MeOH/4:1). ^{1}H NMR (400 MHz, $CDCl_3$): δ = 8.74 (d, *J* = 8.8 Hz, 1H), 8.56 (d, *J* = 6.0 Hz, 1H), 7.87 (d, *J* = 8.1 Hz, 1H), 7.80 – 7.72 (m, 2H), 7.64 (ddd, *J* = 8.2, 6.9, 1.2 Hz, 1H), 7.30 (dd, *J* = 8.5, 6.0 Hz, 1H). ^{13}C NMR (101 MHz, $CDCl_3$): δ = 141.5, 136,0, 130.7, 130.6, 129,0, 128.3, 126.7, 121.1, 120.0. IR (cm^{-1}): $\tilde{\nu}$ = 3465, 3058, 1674, 1577, 1514, 1450, 1396, 1313, 1265, 1230, 1209, 1178, 1142, 1095, 877, 796, 775. MS (70 eV, EI): *m/z* (%): 145 (M^+ + 1, 33%), 130 (10), 128 (22), 102 (24), 90 (14), 89 (11).

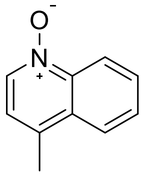

4-methylquinoline 1-oxide (2l). Obtained following procedure B. White solid, 612 mg, 55% isolated yield. Spectroscopic data matched with previously reported on literature.[5] R_f = 0.27 (EtOAc:MeOH/4:1). ^{1}H NMR (400 MHz, $CDCl_3$): δ = 8.74 (d, *J* = 8.3 Hz, 1H), 8.36 (d, *J* = 6.1 Hz, 1H), 7.90 (ddd, *J* = 8.3, 1.4, 0.6 Hz, 1H), 7.70 (ddd, *J* = 8.6, 6,9, 1.4 Hz, 1H), 7.61 (ddd, *J* = 8.3, 6.9, 1.3 Hz, 1H), 7.05 (d, *J* = 6.1 Hz, 1H), 2.59 (s, 3H). ^{13}C NMR (101 MHz, $CDCl_3$): δ = 141.2, 135.1, 134.6, 130.2, 130.0, 128.6, 124.9, 121.6, 120.5, 18.5. IR (cm^{-1}): $\tilde{\nu}$ = 3090, 1563, 1510, 1452, 1423, 1392, 1308, 1273, 1240, 1205, 1178, 1147, 1053, 1018, 823, 802, 701, 687, 642, 619. MS (70 eV, EI): *m/z* (%): 159 (M^+, 77%), 144 (14), 143 (100), 142 (27), 130 (49), 116 (14), 115 (33), 103 (11).

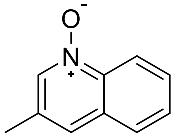

3-methylquinoline 1-oxide (2q). Obtained following procedure B. White solid, 900 mg, 81% isolated yield. Spectroscopic data matched with previously reported on literature.[5] R_f = 0.31 (EtOAc:MeOH/4:1). ^{1}H NMR (300 MHz,

$CDCl_3$): δ = 8.65 (d, *J* = 9.2 Hz, 1H), 8.41 (d, *J* = 1.5 Hz, 1H), 7.75 (dd, *J* = 8.0, 1.5 Hz, 1H), 7.66 (ddd, *J* = 8.6, 6.9, 1.5 Hz, 1H), 7.57 (ddd, *J* = 8.2, 6.9, 1.3 Hz, 1H), 7.51 (d, *J* = 1.1 Hz, 1H), 2.42 (s, 3H). ^{13}C NMR (75 MHz, $CDCl_3$): δ = 139.8, 137.2, 131.3, 130.3, 129.5, 128.9, 127.5, 125.8, 119.7, 18.8. IR (cm^{-1}): $\tilde{\nu}$ = 3053, 1577, 1504, 1371, 1329, 1269, 1217, 1142, 1090, 982, 870, 847, 764, 748. MS (70 eV, EI): *m/z* (%): 159 (M^+ + 1, 100%), 143 (51), 131 (10), 130 (35), 117 (35), 116 (13), 115 (27), 90 (14), 89 (14).

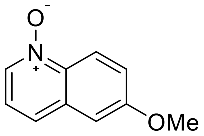

6-methoxyquinoline 1-oxide (2v). Obtained following procedure B. White solid, 847 mg, 69% isolated yield. Spectroscopic data matched with previously reported on literature.[6] R_f = 0.34 (EtOAc:MeOH/4:1). ^{1}H NMR (400 MHz, $CDCl_3$): δ = 8.65 (d, *J* = 9.5 Hz, 1H), 8.39 (d, *J* = 6.0 Hz, 1H), 7.62 (d, *J* = 8.5 Hz, 1H), 7.39-7.35 (m, 1H), 7.26 – 7.21 (m, 1H), 7.10 (d, *J* = 2.7 Hz, 1H), 3.93 (s, 3H). ^{13}C NMR (101 MHz, $CDCl_3$): δ = 159.6, 137.4, 134.0, 132.1, 125.1, 122.9, 121.7, 121.6, 105.9, 55.8. IR (cm^{-1}): $\tilde{\nu}$ = 1620, 1579, 1514, 1473, 1437, 1392, 1369, 1180, 1016, 800, 739.MS (70 eV, EI): *m/z* (%): 175 (M^+ + 1, 100%), 159 (79), 144 (11), 132 (44), 129 (14), 116 (48), 104 (33), 89 (19).

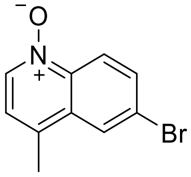

6-bromo-4-methylquinoline 1-oxide (2x). Obtained following procedure B. White solid, 962 mg, 58% isolated yield. R_f = 0.34 (EtOAc:MeOH/4:1). ^{1}H NMR (400 MHz, $CDCl_3$): δ = 8.68 (d, *J* = 9.2 Hz, 1H), 8.42 (d, *J* = 6.1 Hz, 1H), 8.12 (d, *J* = 2.0 Hz, 1H), 7.83 (dd, *J* = 9.3, 2.0 Hz, 1H), 7.14 (d, *J* = 5.1 Hz, 1H), 2.63 (s, 3H). ^{13}C NMR (101 MHz, $CDCl_3$). δ = 140.1, 135.3, 133.6, 133.5, 131.3, 127.4, 123.5, 122.8, 122.6, 18.4. IR (cm^{-1}): $\tilde{\nu}$ = 1560, 1425, 1377, 1350, 1304, 1277, 1232, 1192, 1174, 1144, 839, 818, 768. MS (70 eV, EI): *m/z* (%): 237 (M^+ + 1, 100%), 223 (49), 221 (50), 210 (40), 208 (41), 142 (23), 141 (12), 140 (11), 130 (24), 129 (25), 128 (10), 115 (28), 114 (12), 103 (15), 102 (11), 77 (11). HRMS (EI) calcd. for $C_{10}H_8N$ (M^+ - BrO): 142.0657, found: 142.0657.

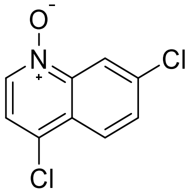

4,7-dichloroquinoline 1-oxide (2y). Obtained following procedure B. White solid, 920 mg, 62% isolated yield. Spectroscopic data matched with previously reported on literature.[7] R_f = 0.36 (EtOAc:MeOH/4:1). ^{1}H NMR (400 MHz, $CDCl_3$): δ = 8.79 (d, *J* = 2.1 Hz, 1H), 8.43 (d, *J* = 6.6 Hz, 1H), 8.15 (d, *J* = 8.9 Hz, 1H), 7.70 (dd, *J* = 9.0, 2.1 Hz, 1H), 7.37 (d, *J* = 6.6 Hz, 1H). ^{13}C NMR (101 MHz, $CDCl_3$): δ = 142.6, 138.4, 136.1, 131.0, 130.0, 126.9, 126.7, 121.4, 120.1. IR (cm^{-1}): $\tilde{\nu}$ = 1606, 1554, 1498, 1350, 1290, 1217, 1153, 1086, 887, 825, 742, 677, 638. MS (70 eV, EI): *m/z* (%): 201 (10), 199 (62), 197 (100), 164 (19), 162 (60), 135 (11), 127 (12), 126 (10), 99 (20), 85 (11).

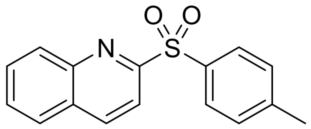

2-tosylquinoline (3a). Obtained following procedure C. White solid, 58.2 mg, 82% isolated yield. Spectroscopic data matched with previously reported on literature.[8] R_f = 0.64 (Hexane:EtOAc/1:1). ^{1}H NMR (400 MHz, $CDCl_3$): δ = 8.36 (d, *J* = 8.5 Hz, 1H), 8.21-8.13 (m, 2H), 8.01 (d, *J* = 8.3 Hz, 2H),

7.86 (d, J = 8.2 Hz, 1H), 7.80-7.74 (m, 1H), 7.67-7.61 (m, 1H), 7.32 (d, J = 8.1 Hz, 2H), 2.39 (s, 3H). ^{13}C NMR (101 MHz, $CDCl_3$): δ 158.4, 147.5, 144.9, 138.8, 136.2, 131.1, 130.5, 129.9, 129.2, 129.2, 128.9, 127.8, 117.8, 21.8. IR (cm^{-1}): $\tilde{\nu}$ = 2922, 1594, 1577, 1496, 1323, 1166, 1149, 1132, 1103, 1074, 1051, 835, 814, 756, 706, 686, 648, 631, 609. MS (70 eV, EI): *m/z* (%): 283 (M^+, 2), 220 (15), 219 (89), 218 (100), 217 (10), 204 (36), 139 (13), 128 (32), 101 (21), 77 (11).

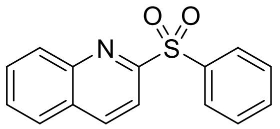

2-(phenylsulfonyl)quinoline (3b). Obtained following general procedure C. White solid, 48.1 mg, 72% isolated yield. Spectroscopic data matched with previously reported on literature.[8] R_f = 0.57 (Hexane:EtOAc/1:1). ^{1}H NMR (400 MHz, $CDCl_3$): δ = 8.38 (d, J = 8.5, 1H), 8.22 (d, J = 8.5 Hz, 1H), 8.19 – 8.13 (m, 3H), 7.91-7.86 (m, 1H), 7.82-7.75 (m, 1H), 7.69-7.64 (m, 1H), 7.63 – 7.59 (m, 1H), 7.56 – 7.52 (m, 2H). ^{13}C NMR (101 MHz, $CDCl_3$): δ = 158.2, 147.6, 139.3, 138.9, 133.9, 131.1, 130.6, 129.4, 129.22, 129.19, 129.0, 127.8, 117.8. IR (cm^{-1}): $\tilde{\nu}$ = 3060, 1618, 1576, 1496, 1446, 1319, 1305, 1163, 1132, 1074, 823, 756, 721, 683. MS (70 eV, EI): *m/z* (%): 205 (60), 204 (100), 128 (28), 101 (13), 77 (10).[9]

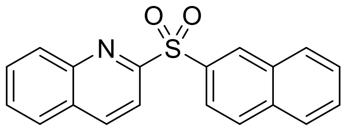

2-(naphthalen-2-ylsulfonyl)quinoline (3c). Obtained following procedure C. White solid, 45.6 mg, 57% isolated yield. Spectroscopic data matched with previously reported on literature.[10] R_f = 0.54 (Hexane:EtOAc/1:1). ^{1}H NMR (400 MHz, $CDCl_3$): δ = 8.75 (d, J = 1.5 Hz, 1H), 8.39 (dd, J = 8.6, 0.9 Hz, 1H), 8.27 (d, J = 8.5 Hz, 1H), 8.17 (dd, J = 8.6, 1.0 Hz, 1H), 8.09 (dd, J = 8.7, 1.9 Hz, 1H), 8.04 – 8.00 (m, 1H), 7.96 (d, J = 8.6 Hz, 1H), 7.90 – 7.86 (m, 2H), 7.80-7.75 (m, 1H), 7.67 – 7.60 (m, 3H). ^{13}C NMR (101 MHz, $CDCl_3$): δ = 158.4, 147.4, 138.9, 136.3, 135.5, 132.4, 131.1, 131.0, 130.6, 129.7, 129.44 (2C), 129.35, 129.0, 128.1, 127.8, 127.7, 123.9, 118.0. IR (cm^{-1}): $\tilde{\nu}$ = 2973, 2021, 1290, 1155, 1086, 1047, 881, 741, 677, 636. MS (70 eV, EI): *m/z* (%): 281 (22), 256 (14), 255 (76), 254 (75), 253 (15), 209 (13), 208 (21), 207 (100), 191 (12), 128 (24), 127 (19), 101 (15), 96 (13), 77 (10), 73 (16).

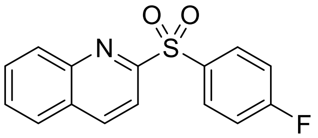

2-[(4-fluorophenyl)sulfonyl]quinoline (3d). Obtained following general procedure C. White solid, 51.1 mg, 71% isolated yield. Spectroscopic data matched with previously reported on literature.[8] R_f = 0.60 (Hexane:EtOAc/1:1). ^{1}H NMR (400 MHz, $CDCl_3$): δ = 8.39 (d, J = 8.0 Hz, 1H), 8.25 – 8.11 (m, 4H), 7.89 (d, J = 8.6 Hz, 1H), 7.80 (ddd, J = 8.5, 6.9, 1.5 Hz, 1H), 7.67 (ddd, J = 8.2, 6.9, 1.2 Hz, 1H), 7.25 – 7.17 (m, 2H). ^{13}C NMR (101 MHz, $CDCl_3$): δ = 166.1 (d, $^1J_{C\text{-}F}$ = 256.4 Hz), 158.2, 147.6, 139.0, 135.2 (d, $^4J_{C\text{-}F}$ = 3.4 Hz), 132.2 (d, $^3J_{C\text{-}F}$ = 9.7 Hz), 131.2, 130.5, 129.4, 129.0, 127.9, 117.6, 116.6 (d, $^2J_{C\text{-}F}$ = 22.8 Hz). ^{19}F NMR (282 MHz, $CDCl_3$): δ = -103.5. IR (cm^{-1}): $\tilde{\nu}$ = 3062, 1585, 1493, 1327, 1294, 1244, 1165, 1128, 1103, 1074, 843, 820, 756, 712, 685. MS (70 eV, EI): *m/z* (%): 281 (12), 224 (12), 223 (74), 222 (100), 208 (10), 207 (46), 143 (23), 129 (18), 128 (66), 102 (15), 101 (40), 95 (10), 77 (18), 75 (22), 73 (13), 44 (11).

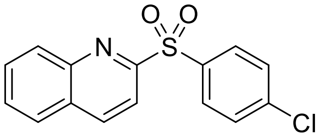

2-((4-chlorophenyl)sulfonyl)quinoline (3e). Obtained following general procedure C. White solid, 67.2 mg, 90% isolated yield. Spectroscopic data matched with previously reported on literature.[8] R_f = 0.62 (Hexane:EtOAc/1:1). ^{1}H NMR (400 MHz, $CDCl_3$): δ = 8.39 (dd, *J* = 8.6, 0.9 Hz, 1H), 8.20 (d, *J* = 8.5 Hz, 1H), 8.15 (d, *J* = 8.6, 1H), 8.11 – 8.05 (m, 2H), 7.89 (dd, *J* = 8.1, 1.4 Hz, 1H), 7.83-7.77 (m, 1H), 7.70-7.65 (m, 1H), 7.54 – 7.48 (m, 2H). ^{13}C NMR (101 MHz, $CDCl_3$): δ = 158.0, 147.6, 140.7, 139.0, 137.7, 131.3, 130.7, 130.5, 129.6, 129.5, 129.0, 127.9, 117.6. IR (cm^{-1}): $\tilde{\nu}$ = 2970, 2925, 1577, 1498, 1321, 1163, 1136, 1088, 1076, 1049, 827, 756. MS (70 eV, EI): *m/z* (%): 242 (29), 241 (33), 239 (100), 238 (99), 207 (18), 204 (52), 159 (13), 142 (36), 128 (56), 102 (11), 101 (32), 100 (15), 77 (11), 75 (13).

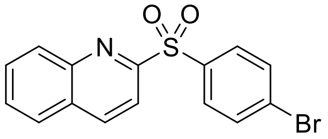

2-((4-bromophenyl)sulfonyl)quinoline (3f). Obtained following general procedure C. White solid, 52.4 mg, 60% isolated yield. Spectroscopic data matched with previously reported on literature.[8] R_f = 0.71 (Hexane:EtOAc/1:1). ^{1}H NMR (400 MHz, $CDCl_3$): δ = 8.40 (d, *J* = 8.6 Hz, 1H), 8.21 (d, *J* = 8.5 Hz, 1H), 8.15 (d, *J* = 8.6 Hz, 1H), 8.04 – 7.97 (m, 2H), 7.89 (d, *J* = 8.7 Hz, 1H), 7.80 (ddd, *J* = 8.5, 6.9, 1.5 Hz, 1H), 7.71 – 7.65 (m, 3H). ^{13}C NMR (101 MHz, $CDCl_3$): δ = 157.9, 147.6, 139.0, 138.2, 132.5, 131.3, 130.8, 130.5, 129.5, 129.4, 129.0, 127.9, 117.7. IR (cm^{-1}): $\tilde{\nu}$ = 2919, 1572, 1323, 1161, 1136, 1068, 1008, 823, 758, 741, 702. MS (70 eV, EI): *m/z* (%): 283 (11), 281 (41), 253 (17), 209 (14), 208 (22), 207 (100), 132 (10), 96 (11), 78 (15), 73 (25), 44 (25).

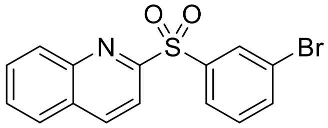

2-((3-bromophenyl)sulfonyl)quinoline (3g). Obtained following procedure C. White solid, 46.7 mg, 54% isolated yield. Spectroscopic data matched with previously reported on literature.[10] R_f = 0.61 (Hexane:EtOAc/1:1). ^{1}H NMR (400 MHz, $CDCl_3$): δ = 8.41 (d, *J* = 8.6 Hz, 1H), 8.28 (dd, *J* = 1.8, 1.7 Hz, 1H), 8.21 (d, *J* = 8.5 Hz, 1H), 8.17 (d, *J* = 8.6 Hz, 1H), 8.08 (ddd, *J* = 7.9, 1.7, 1.0 Hz, 1H), 7.90 (d, *J* = 8.3 Hz, 1H), 7.84-7.78 (m, 1H), 7.75 – 7.66 (m, 2H), 7.42 (t, *J* = 7.9 Hz, 1H). ^{13}C NMR (101 MHz, $CDCl_3$): δ = 157.7, 147.7, 141.1, 139.1, 136.9, 132.0, 131.3, 130.7, 130.6, 129.6, 129.1, 127.9, 127.9, 123.2, 117.8. IR (cm^{-1}): $\tilde{\nu}$ = 2962, 1618, 1571, 1496, 1462, 1383, 1319, 1296, 1240, 1213, 1163, 1134, 1103, 1072, 1028, 885, 837, 793, 771, 756, 673, 640. MS (70 eV, EI): *m/z* (%): 285 (14), 284 (23), 283 (19), 282 (26), 281 (22), 208 (21), 207 (100), 204 (23), 191 (11), 128 (27), 101 (13), 96 (12).

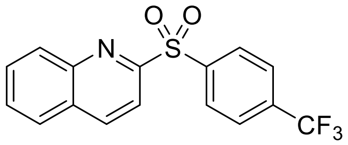

2-((4-(trifluoromethyl)phenyl)sulfonyl)quinoline (3h). Obtained following procedure C. White solid, 51.2 mg, 61% isolated yield. Spectroscopic data matched with previously reported on literature.[10] R_f = 0.61 (Hexane:EtOAc/1:1). ^{1}H NMR (400 MHz, $CDCl_3$): δ = 8.42 (dd, *J* = 8.6, 0.8 Hz, 1H), 8.29 (d, *J* = 7.7 Hz, 2H), 8.24 (d, *J* = 8.6 Hz,

1H), 8.15 (dd, J = 8.3, 1.0 Hz, 1H), 7.90 (dd, J = 8.2, 1.4 Hz, 1H), 7.84 – 7.79 (m, 3H), 7.69 (ddd, J = 8.1, 6.8, 1.2 Hz, 1H). ^{13}C NMR (101 MHz, $CDCl_3$): δ = 157.6, 147.7, 142.8, 139.1, 135.5 (q, $^{2}J_{C\text{-}F}$ = 33.0 Hz), 131.4, 130.5, 129.9 (2C), 129.7, 129.1, 127.9, 126.3 (q, $^{3}J_{C\text{-}F}$ = 3.6 Hz), 117.7 (The expected signal from the CF_3 carbon is missing due to low intensity). ^{19}F NMR (376 MHz, $CDCl_3$): δ = -63.6. IR (cm^{-1}): $\tilde{\nu}$ = 2973, 1379, 1088, 1045, 879. MS (70 eV, EI): *m/z* (%): 272 (85), 207 (41), 204 (17), 145 (10), 129 (14), 128 (100), 116 (12), 77 (17), 75 (19), 44 (26).

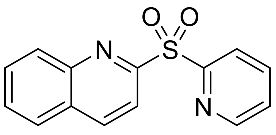

2-(pyridin-2-ylsulfonyl)quinoline (3i). Obtained following general procedure C. White solid, 52.2 mg, 78% isolated yield. Spectroscopic data matched with previously reported on literature.[11] R_f = 0.65 (Hexane:EtOAc/1:1). ^{1}H NMR (400 MHz, $CDCl_3$): δ = 8.63 (ddd, J = 4.8, 1.7, 0.9 Hz, 1H), 8.45 – 8.41 (m, 2H), 8.38 (d, J = 8.5 Hz, 1H), 8.13 (dd, J = 8.6, 1.2 Hz, 1H), 7.99 (td, J = 7.8, 1.7 Hz, 1H), 7.90 (ddd, J = 8.2, 1.5, 0.7 Hz, 1H), 7.77 (ddd, J = 8.5, 6.9, 1.5 Hz, 1H), 7.67 (ddd, J = 8.1, 6.9, 1.2 Hz, 1H), 7.49 (ddd, J = 7.7, 4.7, 1.2 Hz, 1H). ^{13}C NMR (101 MHz, $CDCl_3$): δ = 157.5, 156.7, 150.5, 147.6, 138.7, 138.1, 131.1, 130.5, 129.5, 129.3, 127.9, 127.4, 124.2, 119.4. IR (cm^{-1}): $\tilde{\nu}$ = 3068, 1576, 1495, 1425, 1321, 1171, 1113, 1076, 758, 739. MS (70 eV, EI): *m/z* (%): 253 (16), 208 (12), 207 (64), 206 (77), 205 (100), 129 (11), 128 (36), 101 (15), 73 (13).

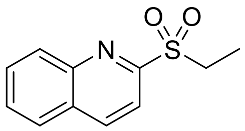

2-(ethylsulfonyl)quinoline (3j). Obtained following procedure C. White solid, 35.5 mg, 65% isolated yield. Spectroscopic data matched with previously reported on literature.[12] R_f = 0.57 (Hexane:EtOAc/1:1). ^{1}H NMR (400 MHz, $CDCl_3$): δ = 8.44 (d, J = 8.5 Hz, 1H), 8.23 (dq, J = 8.5, 0.9 Hz, 1H), 8.14 (d, J = 8.5 Hz, 1H), 7.94 (dd, J = 8.2, 1.4 Hz, 1H), 7.86 (ddd, J = 8.5, 6.9, 1.5 Hz, 1H), 7.72 (ddd, J = 8.1, 6.9, 1.2 Hz, 1H), 3.59 (q, J = 7.5 Hz, 2H), 1.38 (t, J = 7.5 Hz, 3H). ^{13}C NMR (101 MHz, $CDCl_3$): δ = 156.7, 147.4, 138.9, 131.3, 130.4, 129.4, 129.3, 128.0, 117.6, 46.5, 7.1. IR (cm^{-1}): $\tilde{\nu}$ = 1689, 1570, 1506, 1444, 1363, 1267, 1198, 1126, 1061, 877, 839. MS (70 eV, EI): *m/z* (%): 193 (10), 145 (10), 130 (11), 129 (100), 128 (82), 101 (24), 77 (10).

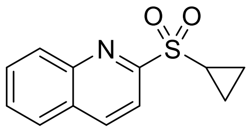

2-(cyclopropylsulfonyl)quinoline (3k). Obtained following procedure C. White solid, 36.2 mg, 62% isolated yield. Spectroscopic data matched with previously reported on literature.[13] R_f = 0.55 (Hexane:EtOAc/1:1). ^{1}H NMR (400 MHz, $CDCl_3$): δ = 8.41 (d, J = 8.6 Hz, 1H), 8.25 (dd, J = 8.6, 1.0 Hz, 1H), 8.06 (d, J = 8.5 Hz, 1H), 7.93 (dd, J = 8.2, 1.4 Hz, 1H), 7.84 (ddd, J = 8.5, 6.9, 1.5 Hz, 1H), 7.70 (ddd, J = 8.2, 6.9, 1.2 Hz, 1H), 2.99 (tt, J = 8.0, 4.8 Hz, 1H), 1.46 – 1.40 (m, 2H), 1.14 – 1.07 (m, 2H). ^{13}C NMR (101 MHz, $CDCl_3$): δ = 157.8, 147.5, 138.8, 131.2, 130.5, 130.0, 129.3, 128.0, 117.4, 29.8, 5.7. IR (cm^{-1}): $\tilde{\nu}$ = 2973, 2881, 2021, 1379, 1325, 1088, 1045, 879. MS (70 eV, EI): *m/z* (%): 169 (23), 168 (100), 129 (19), 128 (57), 101 (22), 77 (10).

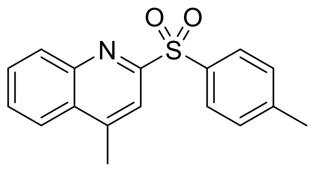

4-methyl-2-tosylquinoline (3l). Obtained following general procedure C. White solid, 60.8 mg, 82% isolated yield. Spectroscopic data matched with previously reported on literature.[11] R_f = 0.66 (Hexane:EtOAc/1:1). ^{1}H NMR (400 MHz, $CDCl_3$): δ = 8.16 (d, J = 8.0 Hz, 1H), 8.04 – 7.99 (m, 4H), 7.78-7.73 (m, 1H), 7.68-7.63 (m, 1H), 7.32 (d, J = 8.2 Hz, 2H), 2.79 (s, 3H), 2.39 (s, 3H). ^{13}C NMR (101 MHz, $CDCl_3$): δ = 158.1, 148.0, 147.4, 144.8, 136.4, 131.2, 130.6, 129.9, 129.2, 129.0, 128.9, 123.9, 118.2, 21.8, 19.3. IR (cm^{-1}): $\tilde{\nu}$ =2969, 2927, 1612, 1577, 1446, 1317, 1161, 1140, 1080, 1045, 860, 808, 762, 725, 685. MS (70 eV, EI): *m/z* (%): 234 (14), 233 (83), 232 (100), 218 (43), 207 (20), 140 (14), 116 (15), 115 (26).

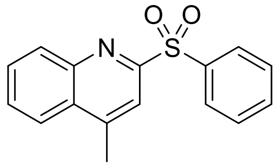

4-methyl-2-(phenylsulfonyl)quinoline (3m). Obtained following general procedure C. White solid, 54.7 mg, 92% isolated yield. Spectroscopic data matched with previously reported on literature.[8] R_f = 0.73 (Hexane:EtOAc/1:1). ^{1}H NMR (400 MHz, $CDCl_3$): δ = 8.17 – 8.12 (m, 3H), 8.05 (q, J = 0.9 Hz, 1H), 8.01 (ddd, J = 8.4, 1.5, 0.7 Hz, 1H), 7.78-7.73 (m, 1H), 7.68-7.63 (m, 1H), 7.61 – 7.56 (m, 1H), 7.55 – 7.50 (m, 2H), 2.79 (d, J = 1.0 Hz, 3H). ^{13}C NMR (101 MHz, $CDCl_3$): δ = 157.8, 148.1, 147.4, 139.4, 133.7, 131.2, 130.7, 129.2, 129.1, 129.0, 128.9, 123.9, 118.2, 19.3. IR (cm^{-1}): $\tilde{\nu}$ = 2970, 2927, 1612, 1576, 1504, 1446, 1313, 1157, 1140, 1111, 1076, 856, 756, 727, 687. MS (70 eV, EI): *m/z* (%): 281 (32), 253 (15), 219 (44), 218 (74), 209 (14), 208 (21), 207 (100), 204 (16), 191 (12), 142 (11), 116 (11), 115 (21), 96 (11), 73 (18), 44 (27).

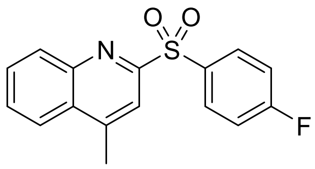

2-((4-fluorophenyl)sulfonyl)-4-methylquinoline (3n). Obtained following general procedure C. White solid, 48.9 mg, 65% isolated yield. R_f = 0.63 (Hexane:EtOAc/1:1). ^{1}H NMR (400 MHz, $CDCl_3$): δ = 8.19 – 8.12 (m, 3H), 8.04 (dd, J = 8.5, 1.3 Hz, 2H), 7.80-7.75 (m, 1H), 7.71-7.66 (m, 1H), 7.21 (t, J = 8.6 Hz, 2H), 2.81 (d, J = 0.9 Hz, 3H). ^{13}C NMR (75 MHz, $CDCl_3$): δ = 166.0 (d, $^1J_{C\text{-}F}$ = 256.3 Hz), 157.7, 148.3, 147.3, 135,3 (d, $^4J_{C\text{-}F}$ = 3.0 Hz), 132.1 (d, $^3J_{C\text{-}F}$ = 9.7 Hz), 131.1, 130.8, 129.1, 129.0, 124.0, 118.0, 116.5 (d, $^2J_{C\text{-}F}$ = 22.7 Hz), 19.3. ^{19}F NMR (377 MHz, $CDCl_3$): δ = -103.6. IR (cm^{-1}): $\tilde{\nu}$ = 2920, 1571, 1495, 1322, 1161, 1136, 1070, 829, 760, 741. MS (70 eV, EI): *m/z* (%): 281 (43), 253 (12), 237 (13), 236 (15), 209 (13), 208 (20), 207 (84), 191 (10), 114 (14), 96 (18), 78 (27). HRMS (EI) calcd. for $C_{16}H_{11}FN$ (M^+ - SO_2H): 236.0872, found: 236.0876.

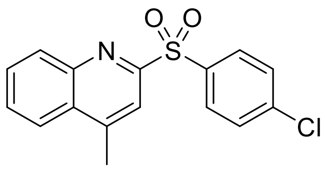

2-((4-chlorophenyl)sulfonyl)-4-methylquinoline (3o). Obtained following general procedure C. White solid, 52.3 mg, 66% isolated yield. Spectroscopic data matched with previously reported on literature.[14] R_f = 0.62 (Hexane:EtOAc/1:1). ^{1}H NMR (400 MHz, $CDCl_3$): δ = 8.14 (d, J = 8.6 Hz, 1H), 8.09 – 8.05 (m, 2H), 8.02 (d, J = 8.6 Hz, 2H), 7.79-7.74 (m, 1H), 7.70-7.64 (m, 1H), 7.52 – 7.48 (m, 2H), 2.80 (s, 3H). ^{13}C NMR (101 MHz, $CDCl_3$): δ = 157.5, 148.3, 147.3, 140.6, 137.8, 131.1, 130.8, 130.6, 129.5, 129.2, 129.0, 124.0, 118.0, 19.3. IR (cm^{-}

^{1}): $\tilde{\nu}$ = 3086, 1576, 1504, 1477, 1315, 1153, 1132, 725, 671. MS (70 eV, EI): *m/z* (%): 255 (29), 254 (47), 253 (90), 252 (100), 238 (28), 218 (19), 217 (11), 207 (17), 159 (12), 142 (26), 140 (25), 116 (31), 115 (55), 89 (13).

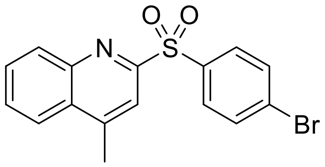

2-((4-bromophenyl)sulfonyl)-4-methylquinoline (3p). Obtained following general procedure C. White solid, 66.2 mg, 74% isolated yield. R_f = 0.71 (Hexane:EtOAc/1:1). ^{1}H NMR (400 MHz, $CDCl_3$): δ = 8.14 (ddd, *J* = 8.5, 1.4, 0.6 Hz, 1H), 8.04 – 7.97 (m, 4H), 7.77 (ddd, *J* = 8.4, 6.9, 1.4 Hz, 1H), 7.70 – 7.65 (m, 3H), 2.80 (d, *J* = 0.9 Hz, 3H). ^{13}C NMR (101 MHz, $CDCl_3$): δ = 157.5, 148.3, 147.4, 140.3, 138.3, 132.5, 131.2, 130.9, 130.8, 129.3, 129.2, 124.0, 118.1, 19.4. IR (cm^{-1}): ṽ = 3095, 1574, 1319, 1155, 1140, 1078, 1066, 1009, 908, 822, 764, 727. MS (70 eV, EI): m/z (%): 341 (22), 299 (11), 298 (15), 297 (12), 296 (14), 282 (14), 281 (41), 253 (27), 208 (21), 207 (100), 191 (11), 135 (14), 115 (10), 73 (23). HRMS (EI) calcd. for $C_{16}H_{12}BrNO_2S$ (M^+): 360.9772, found: 360.9773.

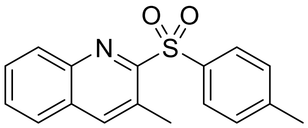

3-methyl-2-tosylquinoline (3q). Obtained following general procedure C. White solid, 45.9 mg, 62% isolated yield. Spectroscopic data matched with previously reported on literature.[11] R_f = 0.65 (Hexane:EtOAc/1:1). ^{1}H NMR (400 MHz, $CDCl_3$): δ = 8.05 (s, 1H), 7.96 – 7.89 (m, 3H), 7.76 (dd, *J* = 8.1, 1.5 Hz, 1H), 7.67-7.62 (m, 1H), 7.60-7.55 (m, 1H), 7.36 (d, *J* = 8.0 Hz, 2H), 2.86 (d, *J* = 1.0 Hz, 3H), 2.46 (s, 3H). ^{13}C NMR (101 MHz, $CDCl_3$): δ = 157.2, 144.8, 144.6, 139.9, 136.0, 130.1, 129.8, 129.6, 129.5, 129.3, 129.1, 128.7, 126.8, 21.8, 19.0. IR (cm^{-1}): $\tilde{\nu}$ = 3066, 1577, 1504, 1446, 1317, 1157, 1140, 1111, 1078, 856, 756, 727, 685. MS (70 eV, EI): *m/z* (%): 233 (45), 232 (100), 218 (19), 207 (28), 115 (16).

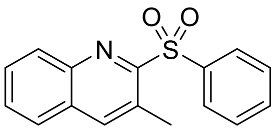

3-methyl-2-(phenylsulfonyl)quinoline (3r). Obtained following general procedure C. White solid, 54.3 mg, 91% isolated yield. Spectroscopic data matched with previously reported on literature.[8] R_f = 0.68 (Hexane:EtOAc/1:1^{1}H NMR (400 MHz, $CDCl_3$): δ = 8.06 (d, *J* = 7.1 Hz, 3H), 7.87 (d, *J* = 7.3 Hz, 1H), 7.75 (dd, *J* = 7.3, 1.2 Hz, 1H), 7.68 – 7.61 (m, 2H), 7.60 – 7.54 (m, 3H), 2.87 (d, *J* = 1.0 Hz, 3H). ^{13}C NMR (101 MHz, $CDCl_3$): δ = 157.0, 144.7, 140.0, 138.9, 133.6, 130.0, 129.9, 129.5, 129.2, 129.1, 128.8, 128.6, 126.8, 18.9. IR (cm^{-1}): $\tilde{\nu}$ = 2927, 1589, 1493, 1452, 1292, 1142, 1092, 1045, 904, 754, 725, 685. MS (70 eV, EI): *m/z* (%): 219 (42), 218 (100), 142 (12), 140 (12), 116 (13), 115 (24).

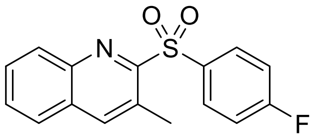

2-((4-fluorophenyl)sulfonyl)-3-methylquinoline (3s). Obtained following general procedure C. White solid, 41.9 mg, 56% isolated yield. R_f = 0.68 (Hexane:EtOAc/1:1). ^{1}H NMR (400 MHz, $CDCl_3$): δ = 8.01 (ddd, *J* = 7.1, 5.1, 2.1 Hz, 3H), 7.77 – 7.73 (m, 1H), 7.70 (dd, *J* = 8.3, 1.4 Hz, 1H), 7.60-7.54 (m, 1H), 7.55-7.48 (m, 1H), 7.21 – 7.16 (m, 2H), 2.83 (d, *J* = 1.0 Hz, 3H). ^{13}C NMR (101 MHz, $CDCl_3$): δ = 166.1 (d, *J* = 255.8 Hz), 157.2, 144.6, 140.0, 134.7, 134.6, 132.6 (d, *J* = 9.6 Hz),

130.0 (d, J = 1.5 Hz), 129.2, 129.2, 128.5, 126.9, 116.1 (d, J = 22.7 Hz), 18.8. ^{19}F NMR (377 MHz, $CDCl_3$): δ = -104.1. IR (cm^{-1}): $\tilde{\nu}$ = 2924, 1589, 1493, 1284, 1234, 1140, 1090, 1043, 835, 756, 673. MS (70 eV, EI): *m/z* (%): 237 (46), 236 (100), 142 (12), 140 (11), 116 (12), 115 (22). HRMS (EI) calcd. for $C_{16}H_{11}FN$ (M^+ - SO_2H): 236.0876, found: 236.0877.

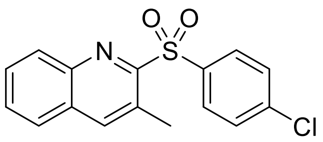

2-((4-chlorophenyl)sulfonyl)-3-methylquinoline (3t). Obtained following general procedure C. White solid, 61.9 mg, 78% isolated yield. R_f = 0.68 (Hexane:EtOAc/1:1). ^{1}H NMR (400 MHz, $CDCl_3$): δ = 8.08 (d, J = 1.0 Hz, 1H), 8.03 – 7.96 (m, 2H), 7.84 (dd, J = 8.3, 1.2 Hz, 1H), 7.77 (dd, J = 8.3, 1.5 Hz, 1H), 7.68-7.61 (m, 1H), 7.62 – 7.53 (m, 3H), 2.90 (s, 3H). ^{13}C NMR (101 MHz, $CDCl_3$): δ = 157.0, 144.6, 140.4, 140.1, 137.3, 131.2, 130.0, 129.9, 129.2 (2C), 129.1, 128.5, 126.9, 18.7. IR (cm^{-1}): $\tilde{\nu}$ = 2933, 1574, 1489, 1442, 1389, 1308, 1281, 1144, 1088, 1066, 1041, 1009, 904, 825, 754, 746. MS (70 eV, EI): *m/z* (%): 281 (30), 255 (16), 254 (42), 253 (56), 252 (100), 218 (10), 209 (12), 208 (19), 207 (91), 191 (10), 142 (12), 140 (11), 116 (13), 115 (23), 96 (10), 73 (17), 44 (10). HRMS (EI) calcd. for $C_{16}H_{11}ClN$ (M^+ - SO_2H): 252.0580, found: 252.0589.

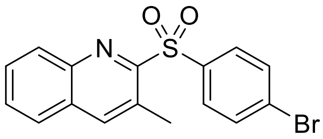

2-((4-bromophenyl)sulfonyl)-3-methylquinoline (3u). Obtained following general procedure C. White solid, 68.0 mg, 75% isolated yield. R_f = 0.71 (Hexane:EtOAc/1:1). ^{1}H NMR (400 MHz, $CDCl_3$): δ = 8.09 (s, 1H), 7.92 (dd, J = 8.7, 4.8 Hz, 2H), 7.87 – 7.82 (m, 1H), 7.77 (dd, J = 8.1, 1.1 Hz, 1H), 7.72 (dd, J = 8.7, 4.8 Hz, 2H), 7.68-7.62 (m, 1H), 7.62-7.57 (m, 1H), 2.90 (d, J = 1.0 Hz, 3H). ^{13}C NMR (101 MHz, $CDCl_3$): δ = 156.9, 144.6, 140.1, 137.8, 132.1, 131.3, 130.0, 129.9, 129.6, 129.3, 129.1, 128.5, 126.9, 18.7. IR (cm^{-1}): $\tilde{\nu}$ = 2929, 1574, 1488, 1469, 1444, 1389, 1309, 1142, 1088, 1068, 1041, 1009, 742. MS (70 eV, EI): *m/z* (%): 299 (26), 298 (65), 297 (28), 296 (65), 282 (10), 281 (35), 253 (15), 218 (27), 217 (15), 209 (13), 208 (22), 207 (100), 191 (13), 142 (14), 140 (15), 116 (16), 115 (30), 96 (13), 73 (18), 44 (10). HRMS (EI) calcd. for $C_{16}H_{12}BrNO_2S$ (M^+): 360.9772, found: 360.9759.

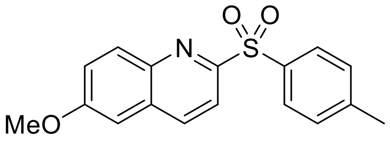

6-methoxy-2-tosylquinoline (3v). Obtained following procedure C. White solid, 62.2 mg, 80% isolated yield. Spectroscopic data matched with previously reported on literature.[13] R_f = 0.60 (Hexane:EtOAc/1:1). ^{1}H NMR (400 MHz, $CDCl_3$): δ = 8.21 (d, J = 7.9 Hz, 1H), 8.13 (d, J = 8.5 Hz, 1H), 8.05 (d, J = 9.3 Hz, 1H), 8.02 – 7.97 (m, 2H), 7,41 (dd, J = 9.3, 2.8 Hz, 1H), 7.31 (d, J = 7.9 Hz, 2H), 7.08 (d, J = 2.8 Hz, 1H), 3.94 (s, 3H), 2.39 (s, 3H). ^{13}C NMR (101 MHz, $CDCl_3$): δ = 159.9, 155.9, 144.7, 143.8, 136.9, 136.7, 132.0, 130.5, 129.9, 129.0, 124.4, 118.4, 104.7, 55.9, 21.8. IR (cm^{-1}): $\tilde{\nu}$ = 2962, 1618, 1595, 1498, 1468, 1385, 1317, 1298, 1240, 1163, 1130, 1074, 1026. MS (70 eV, EI): *m/z* (%): 281 (18), 253 (12), 250 (18), 249 (100), 248 (65), 235 (10), 218 (35), 209 (10), 208 (16), 207 (78), 206 (24), 191 (13), 158 (26), 146 (16), 143 (11), 115 (15), 103 (10), 73 (10).

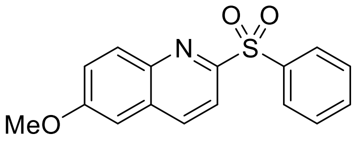

6-methoxy-2-(phenylsulfonyl)quinoline (3w). Obtained following procedure C. White solid, 53.7 mg, 72% isolated yield. Spectroscopic data matched with previously reported on literature.[15] R_f = 0.64 (Hexane:EtOAc/1:1). ^{1}H NMR (400 MHz, $CDCl_3$): δ = 8.21 (d, *J* = 8.7 Hz, 1H), 8.16 – 8.10 (m, 3H), 8.05 – 8.02 (m, 1H), 7.60 – 7.55 (m, 1H), 7.54 – 7.48 (m, 2H), 7.40 (dd, *J* = 9.3, 2.8 Hz, 1H), 7.08 (d, *J* = 2.8 Hz, 1H), 3.92 (s, 3H). ^{13}C NMR (101 MHz, $CDCl_3$): δ = 160.0, 155.6, 143.8, 139.7, 137.0, 133.6, 131.9, 130.6, 129.2, 129.0, 124.4, 118.4, 104.8, 55.9. IR (cm^{-1}): $\tilde{\nu}$ = 1618, 1498, 1469, 1446, 1385, 1308, 1240, 1163, 1136, 1074, 1024, 844, 804, 735, 719, 680, 640. MS (70 eV, EI): *m/z* (%): 281 (21), 234 (11), 209 (13), 208 (21), 207 (100), 191 (12), 96 (11).

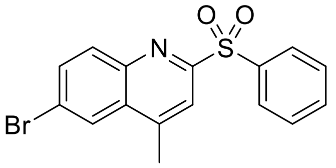

6-bromo-4-methyl-2-(phenylsulfonyl)quinoline (3x). Obtained following procedure C. White solid, 56.4 mg, 63% isolated yield. R_f = 0.59 (Hexane:EtOAc/1:1). ^{1}H NMR (400 MHz, $CDCl_3$): δ = 8.17 (d, *J* = 2.1 Hz, 1H), 8.14 – 8.11 (m, 2H), 8.07 (d, *J* = 1.0 Hz, 1H), 8.01 (d, *J* = 9.0 Hz, 1H), 7.82 (dd, *J* = 9.0, 2.2 Hz, 1H), 7.64 – 7.59 (m, 1H), 7.57 – 7.52 (m, 2H), 2.76 (d, *J* = 0.9 Hz, 3H). ^{13}C NMR (101 MHz, $CDCl_3$): δ = 158.3, 147.3, 146.0, 139.1, 134.3, 133.9, 132.8, 130.1, 129.3, 129.2, 126.5, 123.7, 119.08, 19.30. IR (cm^{-1}): $\tilde{\nu}$ = 3095, 3045, 2023, 1606, 1554, 1498, 1350, 1290, 1240, 1217, 1153, 1086, 887, 825, 742, 677, 638. MS (70 eV, EI): *m/z* (%): 299 (50), 298 (77), 297 (52), 296 (72), 284 (16), 282 (18), 219 (17), 218 (88), 217 (19), 208 (14), 207 (48), 142 (13), 141 (100), 140 (71), 129 (18), 125 (31), 115 (20), 114 (46), 113 (19), 77 (24), 51 (18). HRMS (EI) calcd. for $C_{16}H_{12}N$ (M^+ - CH_2SO_2Br): 218.0970, found: 218.0972.

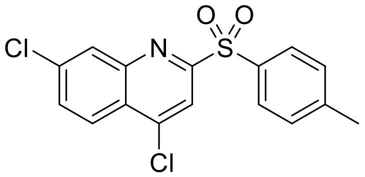

4,7-dichloro-2-tosylquinoline (3y). Obtained following procedure C. White solid, 46.6 mg, 53% isolated yield. Spectroscopic data matched with previously reported on literature.[16] R_f = 0.59 (Hexane:EtOAc/1:1). ^{1}H NMR (400 MHz, $CDCl_3$): δ = 8.27 (s, 1H), 8.21 – 8.16 (m, 2H), 8.00 (d, *J* = 8.3 Hz, 2H), 7.68 (dd, *J* = 9.0, 2.1 Hz, 1H), 7.35 (d, *J* = 8.2 Hz, 2H), 2.42 (s, 3H). ^{13}C NMR (101 MHz, $CDCl_3$): δ = 159.7, 148.6, 145.5, 145.5, 138.4, 135.4, 131.3, 130.1, 129.7, 129.5, 125.72, 125.70, 118.2, 21.9. IR (cm^{-1}): $\tilde{\nu}$ = 3091, 2920, 1599, 1560, 1549, 1483, 1446, 1402, 1329, 1296, 1257, 1147, 1111, 1078, 980, 910, 872, 848, 820, 717, 679, 642, 621. MS (70 eV, EI): *m/z* (%): 319 (18), 318 (12), 317 (49), 281 (14), 253 (12), 252 (13), 238 (21), 219 (17), 218 (100), 208 (16), 207 (72), 162 (14), 139 (48), 135 (18), 127 (10), 99 (18), 91 (32), 65 (11).

Green Metrics calculations.

Yield = $\frac{obtained\ mass\ of\ product}{maximum\ theoretical\ mass\ desired\ product} x100$

Yield CA = $\frac{992\ mg\ of\ \mathbf{3a}}{1417\ mg\ of\ \mathbf{3a}} x100 = 70\%$

Yield OA = $\frac{1051\ mg\ of\ \mathbf{3a}}{1501\ mg\ of\ \mathbf{3a}} x100 = 70\%$

RME (%) = $\frac{obtained\ mass\ of\ product}{mass\ of\ reactants} x100$

RME CA = $\frac{991.7\ mg\ of\ \mathbf{3a}}{2227\ mg\ of\ \mathbf{1a} + 725.8\ mg\ of\ \mathbf{2a}} x100 = 34\%$

RME OA = $\frac{1051\ mg\ of\ \mathbf{3a}}{2370\ mg\ of\ \mathbf{1a} + 769\ mg\ of\ \mathbf{2a}} x100 = 34\%$

PMI = $\frac{obtained\ mass\ of\ reagents + mass\ of\ catalysts + mass\ of\ solvents}{mass\ of\ isolated\ product}$

PMI CA = $\frac{2.2\ \text{g of }\mathbf{1a} + 0.72\ \text{g of }\mathbf{2a}+\mathbf{0}.33\ \text{g TBABF}_4 + 1\ 0.5\ \text{g AcOH} + 10.0\ \text{g H}_2\text{O} + 62.9\ \text{g MeCN}}{0.99\ \text{g of }\mathbf{3a}} = 87.40$

PMI OA = $\frac{2.4\ \text{g of }\mathbf{1a} + 0.77\ \text{g of }\mathbf{2a}+\mathbf{12.4}\ \text{g DES} + 10.0\ \text{g H}_2\text{O}}{1.05\ \text{g of }\mathbf{3a}} = 24.32$

E-Factor = $\frac{total\ mass\ of\ waste}{isolated\ mass\ of\ product}$

E-Factor CA= $\frac{0.67\ \text{g of }\mathbf{1a}+0.22\ \text{g of }\mathbf{2a}+\mathbf{0}.33\ \text{g TBABF}_4+10.5\ \text{g AcOH}+10.0\ \text{g H}_2\text{O}+62.9\ \text{g MeCN}+0.60\ \text{g }p\text{TsOH}}{0.99\ \text{g of }\mathbf{3a}} = 85.93$

E-Factor OA= $\frac{0.71\ \text{g of }\mathbf{1a}+0.23\ \text{g of }\mathbf{2a}+12.5\ \text{g DES}+10.0\ \text{g H}_2\text{O}+0.64\ \text{g }p\text{TsOH}}{1.05\ \text{g of }\mathbf{3a}} = 22.84$

Ecoscale[17]

Classical Approach

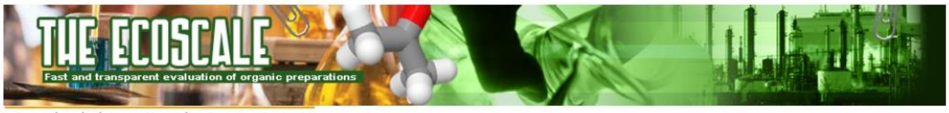

Ecoscale calculator   Manual   Paper   Contact

Reagents

☑ Link

		identifier*	name	MF*	MW	density	purity*	ml	g
1	+ -		4-Methylbenzenesulfinic acid, sodium salt	C7H8O2NaS	179.18909		100%	0	2.239864
2	+ -		Quinoline-N-oxide hydrate	C9H7NO . xH2O			100%	0	0
3	+ -		Tetrabutylammonium tetrafluoroborate	C16H36N . BF4	329.272152		100%	0	0.329272
4	+ -		Acetic acid	C2H4O2	60.05256	1.048	100%	10	10.48
5	+ -		Water	H2O	18.01528	1	100%	10	10
6	+ -		Acetonitrile	CH3CN	41.05252	0.781	100%	80	62.48

Products

identifier*:	name:	MF*:	MW:	g:	mmoles:	g theor:	y
	2-[(4-Methylphenyl)sulfonyl]quinoline	C16H13NO2S	283.35	0.991725	3.5	1.41675	[illegible]

Conditions

Reagents

Name	mmoles	eq.	Bp	Hazard	Price
4-Methylbenzenesulfinic acid, sodium salt	12.6	2.5			
Quinoline-N-oxide hydrate	5.04	1			
Tetrabutylammonium tetrafluoroborate	1	0.2			
Acetic acid	175.96	34.9	117		
Water	559.71	111.01			
Acetonitrile	1534.65	304.39	81		

Yield	70		-15
Price / availability			-16
Safety			-15
Technical setup	Possible items: Common set-up; Instruments for controlled addition of chemicals; Unconventional activation technique	Selected items: (Inert) gas atmosphere	-1
Temperature / time	Possible items: Room temperature, < 1h; Room temperature, < 24h; Heating, < 1h	Selected items: Room temperature, < 24h	-1
Workup and purification	Possible items: Sublimation; Liquid - liquid extraction or washing; Classical chromatography	Selected items: Classical chromatography	-10
EcoScale			42

Copyright 2006

Our Approach

THE ECOSCALE
Fast and transparent evaluation of organic preparations

Ecoscale calculator   Manual   Paper   Contact

Reagents

☑ Link

	identifier*	name	MF*	MW	density	purity*	ml	g
1		4-Methylbenzenesulfinic acid, sodium salt	C7H8O2NaS	179.18909		100%	0	2.239864
2		Quinoline-N-oxide hydrate	C9H7NO . xH2O			100%	0	0
3		Water	H2O	18.01528	1	100%	10	10
4		Tetrabutylammonium bromide	C16H36BrN	322.37254		100%	0	7.86
5		Ethylene glycol	C2H6O2	62.06844	1.113	100%	4.079066	4.54

Products

identifier*:	name:	MF*:	MW:	g:	mmoles:	g theor:	y
	2-[(4-Methylphenyl)sulfonyl]quinoline	C16H13NO2S	283.35	0.991725	3.5	1.41675	7

Conditions

Reagents

Name	mmoles	eq.	Bp	Hazard	Price
4-Methylbenzenesulfinic acid, sodium salt	12.6	2.5			
Quinoline-N-oxide hydrate	5.04	1			
Water	559.71	111.01			
Tetrabutylammonium bromide	24.58	4.87			
Ethylene glycol	73.75	14.62	196		

	Possible items	Selected items	Penalty
Yield	70		-15
Price / availability			-14
Safety			-5
Technical setup	Any additional special glassware<br>(Inert) gas atmosphere<br>Glove box	Unconventional activation technique	-2
Temperature / time	Room temperature, < 1h<br>Room temperature, < 24h<br>Heating, < 1h	Room temperature, < 24h	-1
Workup and purification	None<br>Cooling to room temperature<br>Adding solvent	Simple filtration<br>Adding solvent	0
**EcoScale**			63

Copyright 2006

Table S2. Summary of Green Metrics.

	Classical Approach	Our Approach
**Yield / %**	70	70
**REM / %**	34	34
**PMI / %**	87	24
**E-Factor**	86	23
**EcoScale**	42	63

References

[1] T. C. Johnson, Bryony L. Elbert, A. J. M. Farley, T. W. Gorman, C. Genicot, B. Lallemand, P. Pasau, J. Flasz, J. L. Castro, M. MacCoss, D. J. Dixon, R. S. Paton, C. J. Schofield, M. D. Smith, M. C. Willis, *Chem. Sci.* **2018**, *9*, 629-633.

[2] A. A. Borovleva, E. K. Avakyan, G. A. Amangasieva, O. P. Demidov, D. Y. Pobedinskaya, A. P. Ermolenko, A. N. Larin, I. V. Borovlev, *Chem. Heterocycl. Compd.* **2022**, *58*, 235-242.

[3] A. U. Meyer, S. Jäger, D. Prasad Hari, B. König, *Adv. Synth. Catal.* **2015**, *357*, 2050-2054.

[4] Y.-X. Chen, Z.-J. Wang, J.-A. Xiao, K. Chen, H.-Y. Xiang, H. Yang, *Org. Lett.* **2021**, *23*, 6558-6562.

[5] Y. Zhang, S. Zhang, G. Xu, M. Li, C. Tang, W. Fan, *Org. Biomol. Chem.* **2019**, *17*, 309-314.

[6] C. Sen, S. C. Ghosh, *Adv. Synth. Catal.* **2018**, *360*, 905-910.

[7] L. Bering, A. P. Antonchick, *Org. Lett.* **2015**, *17*, 3134-3137.

[8] M. Jiang, Y. Yuan, T. Wang, Y. Xiong, J. Li, H. Guo, A. Lei, *Chem. Commun.* **2019**, *55*, 13852-13855.

[9] P. Li, Y. Jiang, H. Li, W. Dong, Z. Peng, D. **2018**, *48*, 1909-1918.

[10] L.-Y. Xie, Y.-J. Li, J. Qu, Y. Duan, J. Hu, K.-J. Liu, Z. Cao, W.-M. He, *Green Chem.* **2017**, *19*, 5642-5646.

[11] R. Wang, Z. Zeng, C. Chen, N. Yi, J. Jiang, Z. Cao, W. Deng, J. Xiang, *Org. Biomol. Chem.* **2016**, *14*, 5317-5321.

[12] K. Sun, X.-L. Chen, X. Li, L.-B. Qu, W.-Z. Bi, X. Chen, H.-L. Ma, S.-T. Zhang, B.-W. Han, Y.-F. Zhao, C.-J. Li, *Chem. Commun.* **2015**, *51*, 12111-12114.

[13] B. Du, P. Qian, Y. Wang, H. Mei, J. Han, Y. Pan, *Org. Lett.* **2016**, *18*, 4144-4147.

[14] W.-K. Fu, K. Sun, C. Qu, X.-L. Chen, L.-B. Qu, W.-Z. Bi, Y.-F. Zhao, *Asian J. Org. Chem.* **2017**, *6*, 492-495.

[15] Y. Su, X. Zhou, C. He, W. Zhang, X. Ling, X. Xiao, *J. Org. Chem.* **2016**, *81*, 4981-4987.

[16] L. Wang, Z. Ning, Z. Xu, R. Liu, Z. Du, *ChemistrySelect* **2023**, *8*, e202205013.

[17] K. Van Aken, L. Strekowski, L. Patiny, *Beilstein J. Org. Chem.* **2006**, *2*, 3.

Copies of ^{1}H NMR and ^{13}C NMR

^{1}H NMR (400 MHz, D_2O) (**1c**)

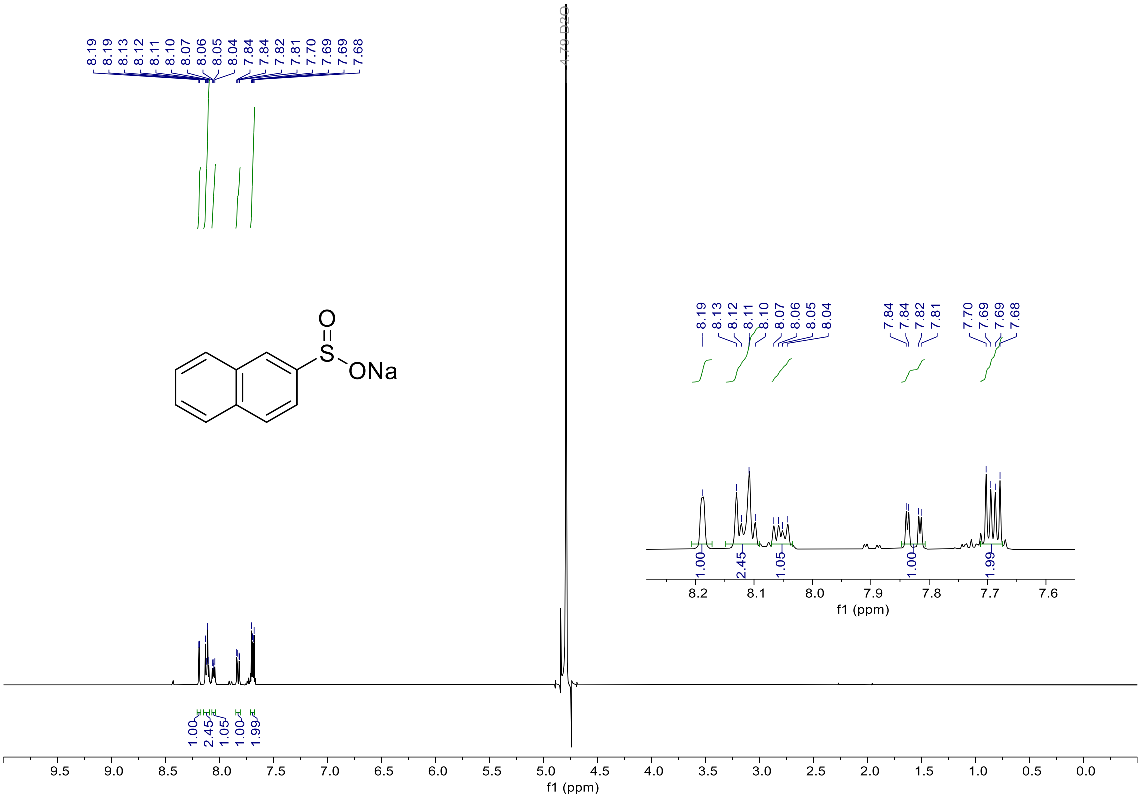

^{13}C NMR (101 MHz, MeOD) (**1c**)

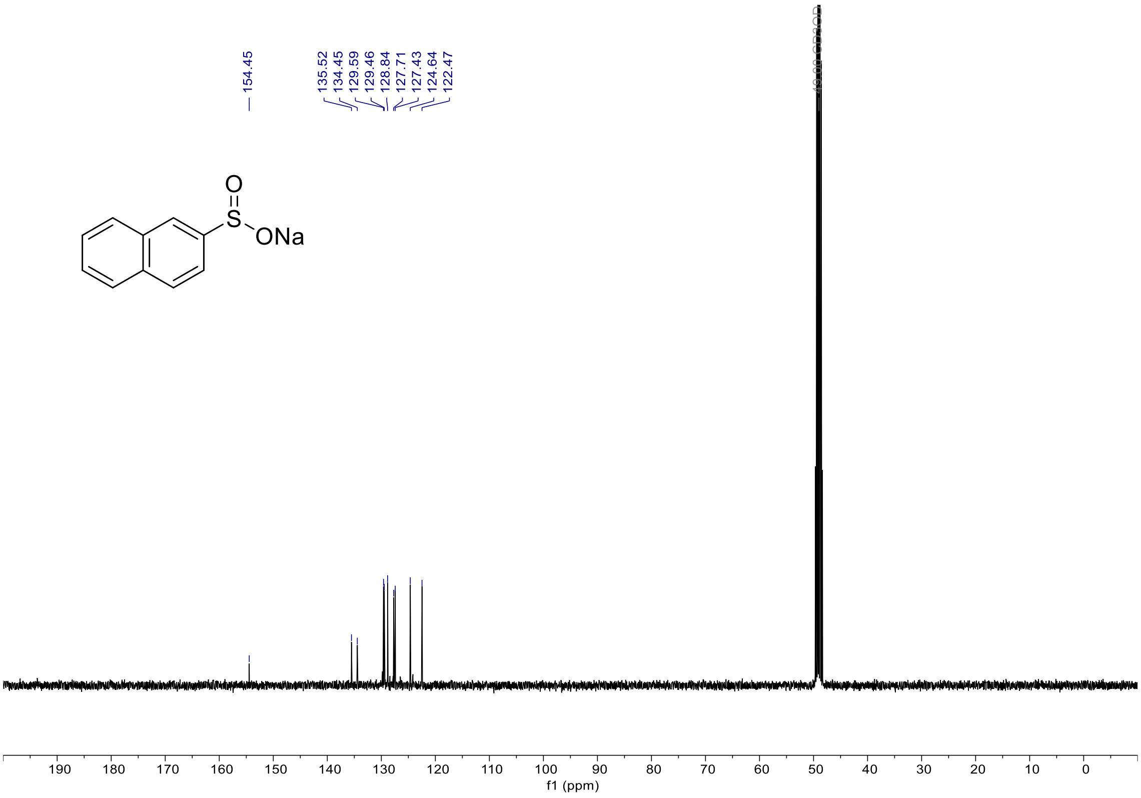

^{1}H NMR (400 MHz, D_2O) (**1g**)

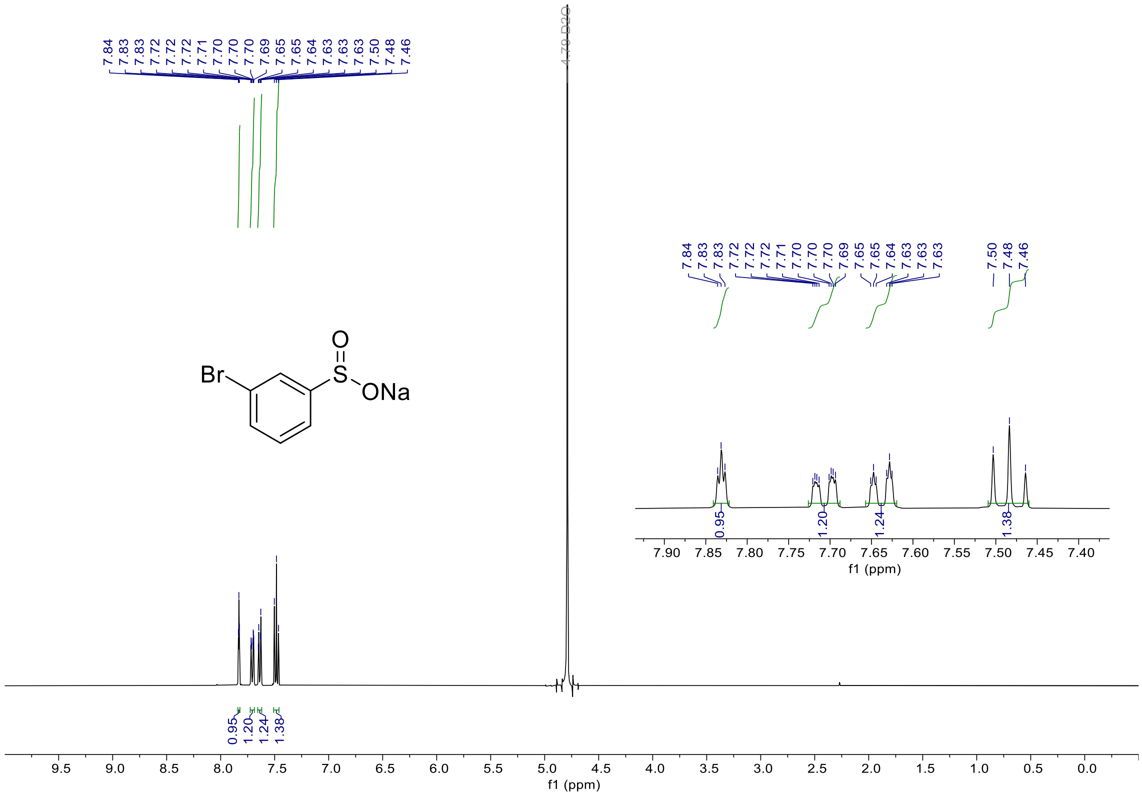

^{13}C NMR (101 MHz, MeOD) (**1g**)

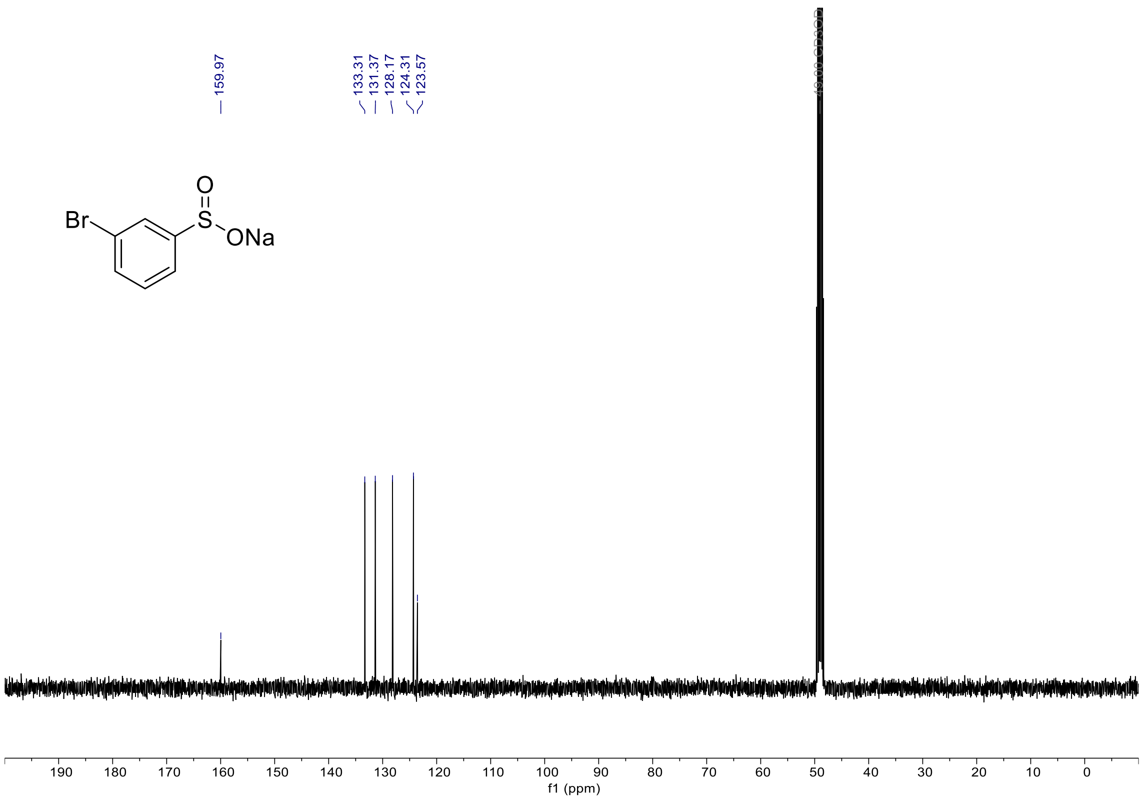

^{1}H NMR (400 MHz, $CDCl_3$) (**2a**)

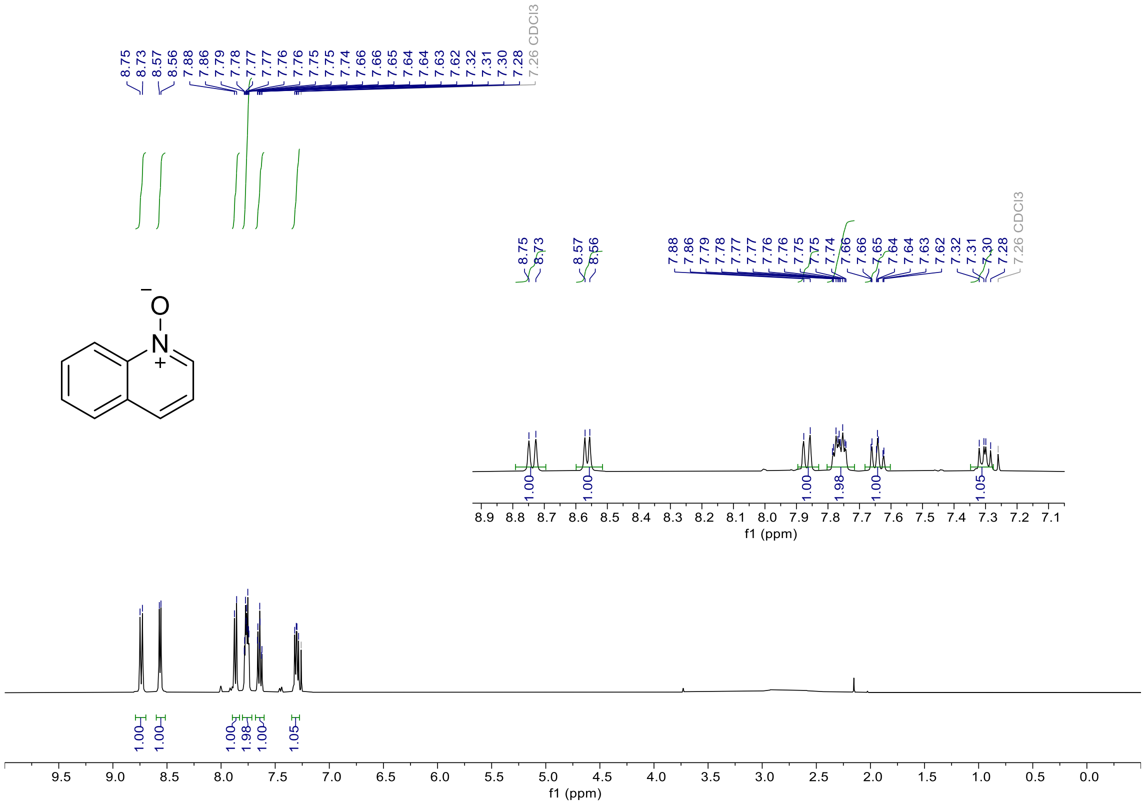

^{13}C NMR (101 MHz, $CDCl_3$) (**2a**)

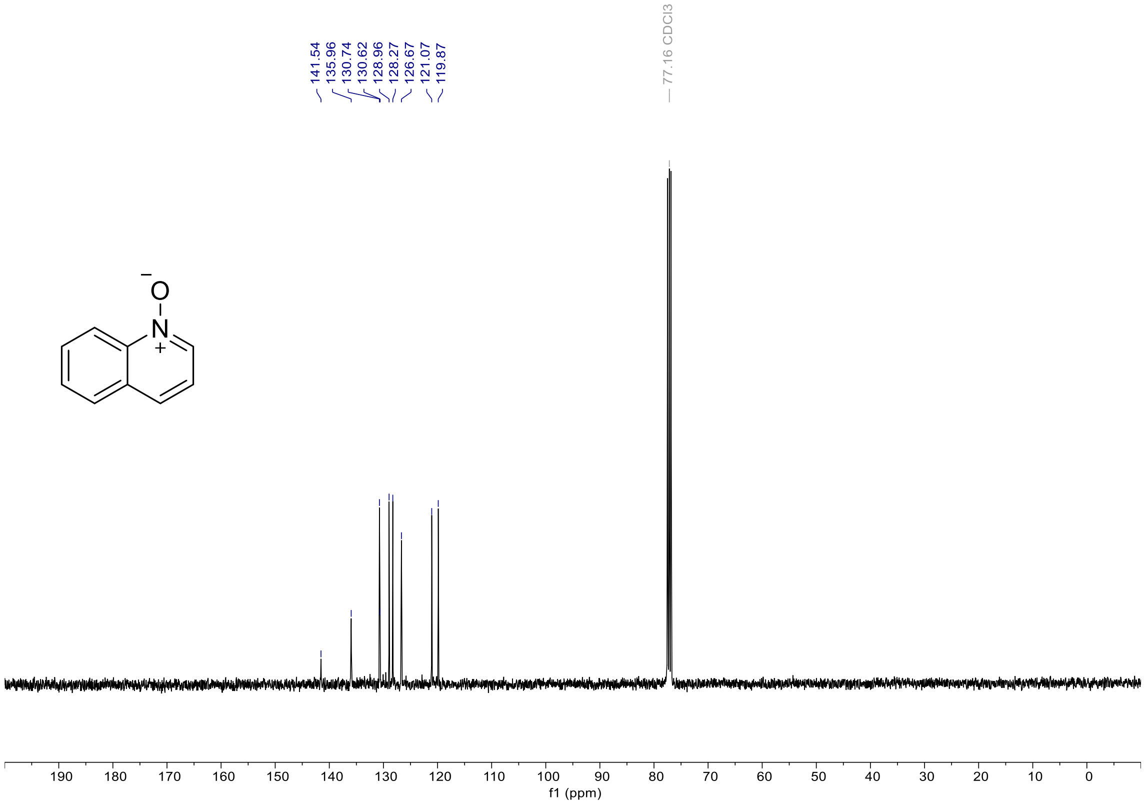

^{1}H NMR (400 MHz, $CDCl_3$) (**2l**)

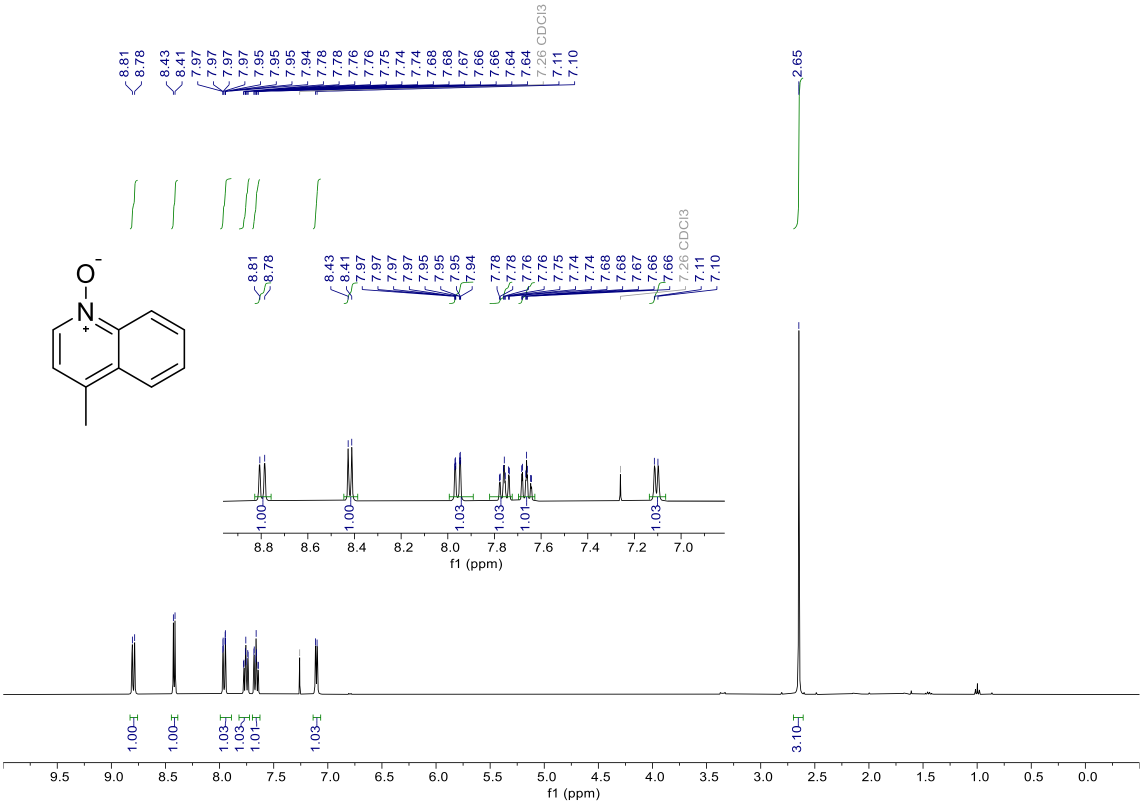

^{13}C NMR (101 MHz, $CDCl_3$) (**2l**)

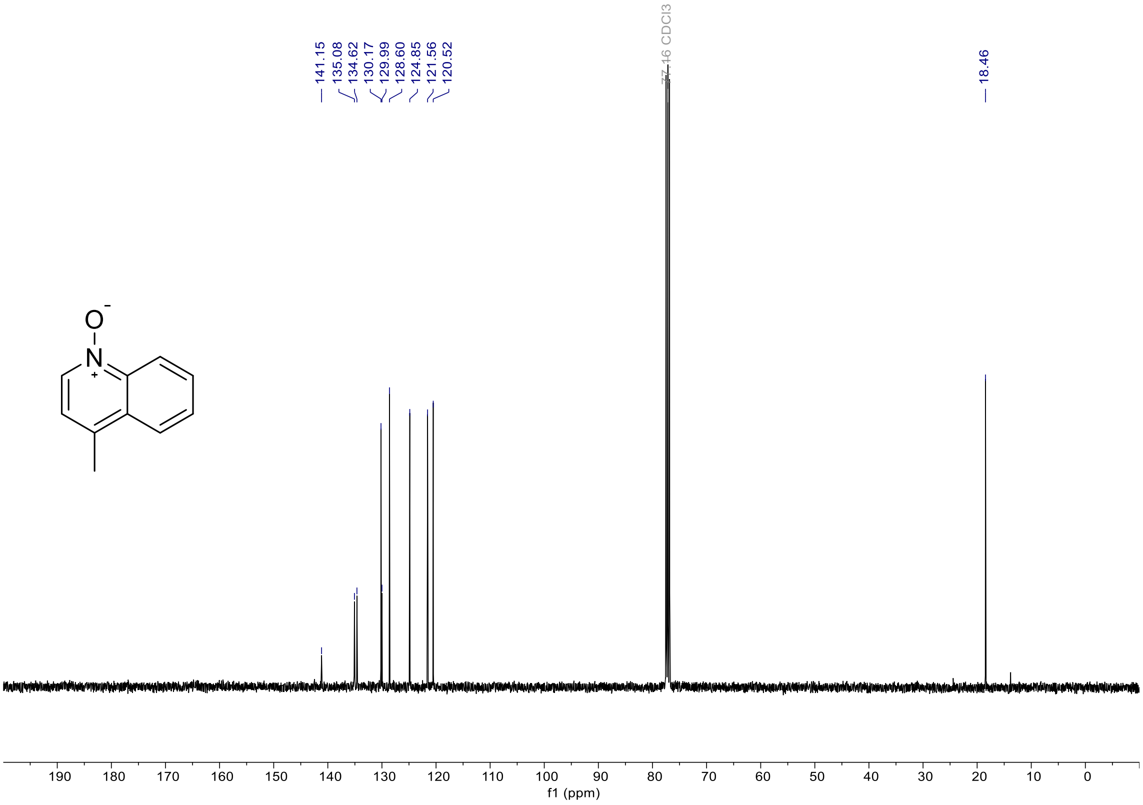

^{1}H NMR (400 MHz, $CDCl_3$) (**2q**)

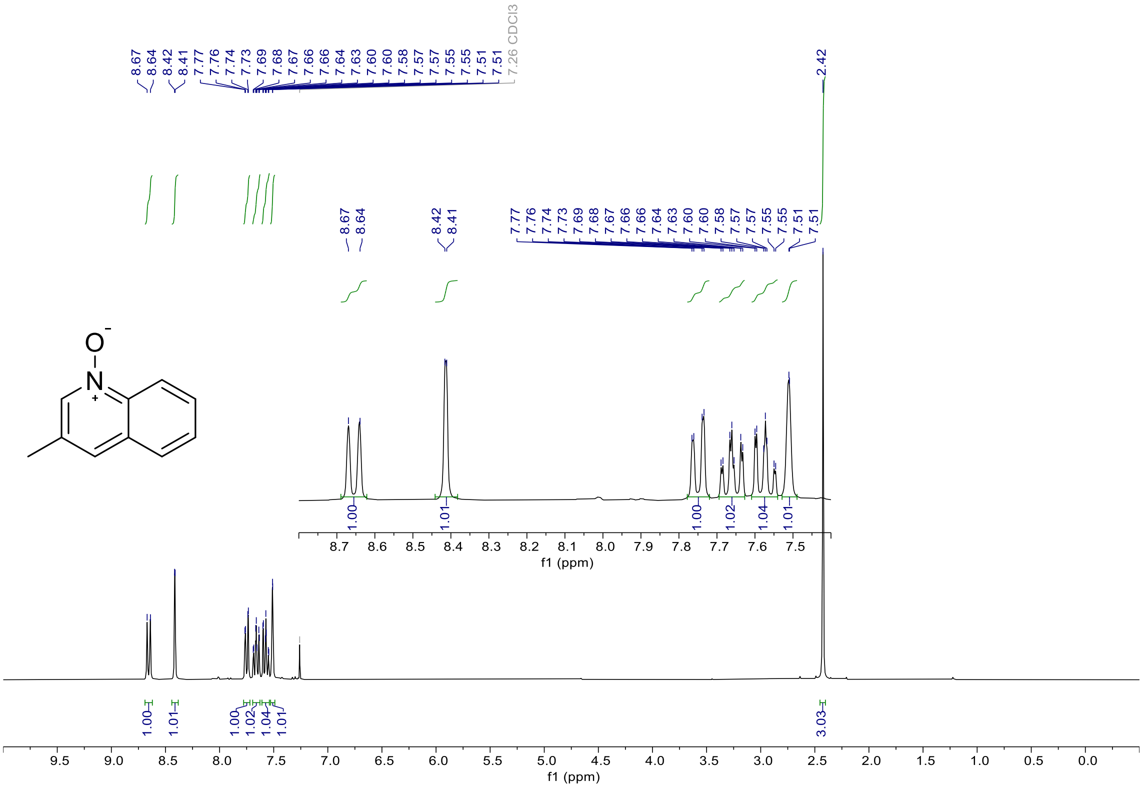

^{13}C NMR (101 MHz, $CDCl_3$) (**2q**)

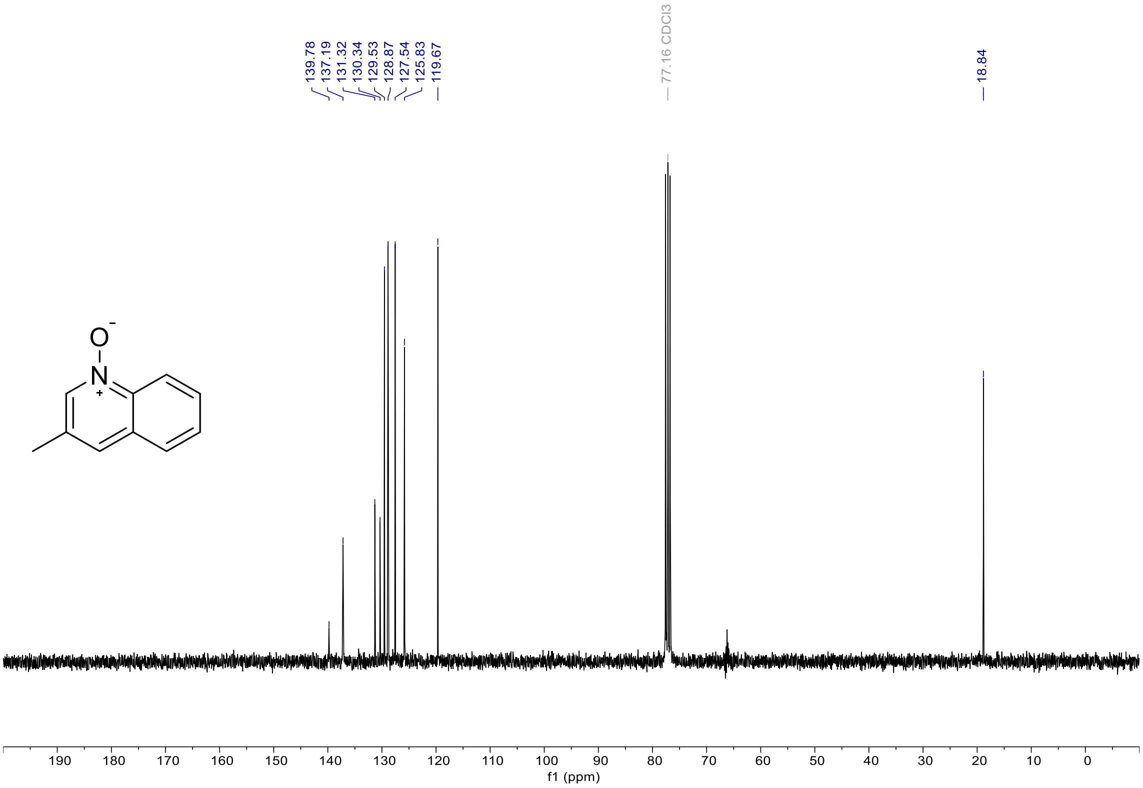

^1H NMR (400 MHz, $CDCl_3$) (**2v**)

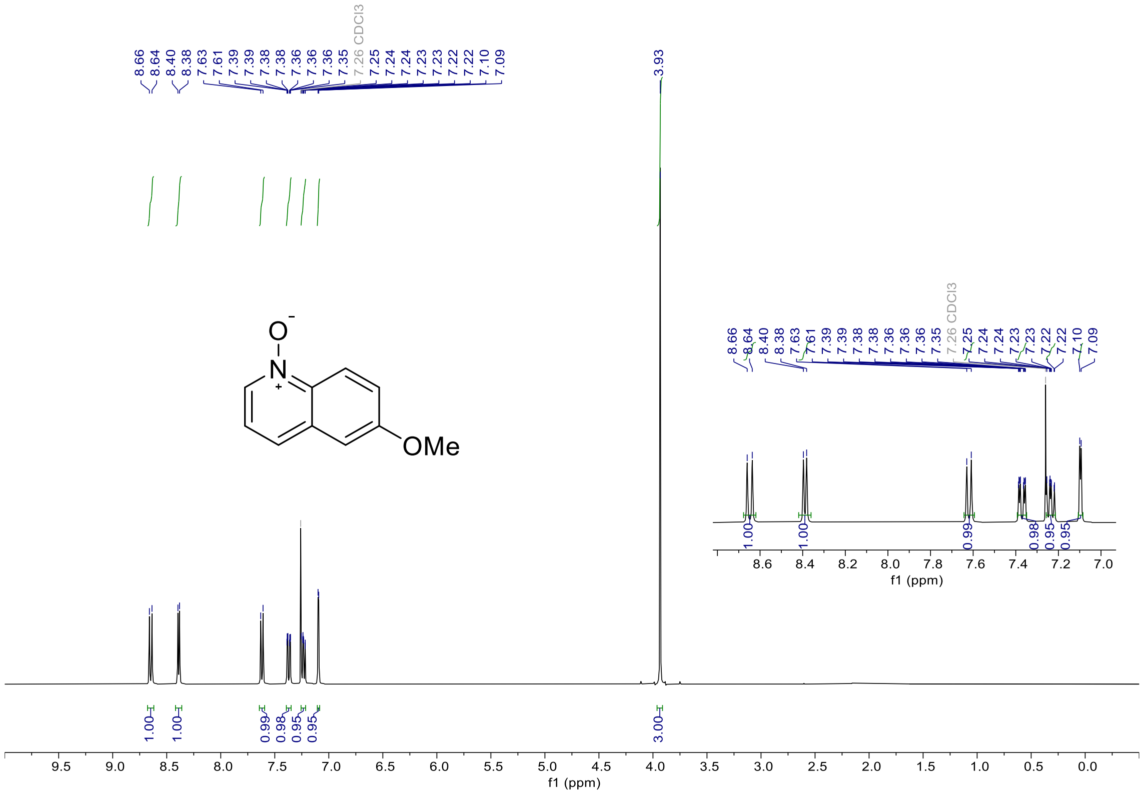

^{13}C NMR (101 MHz, $CDCl_3$) (**2v**)

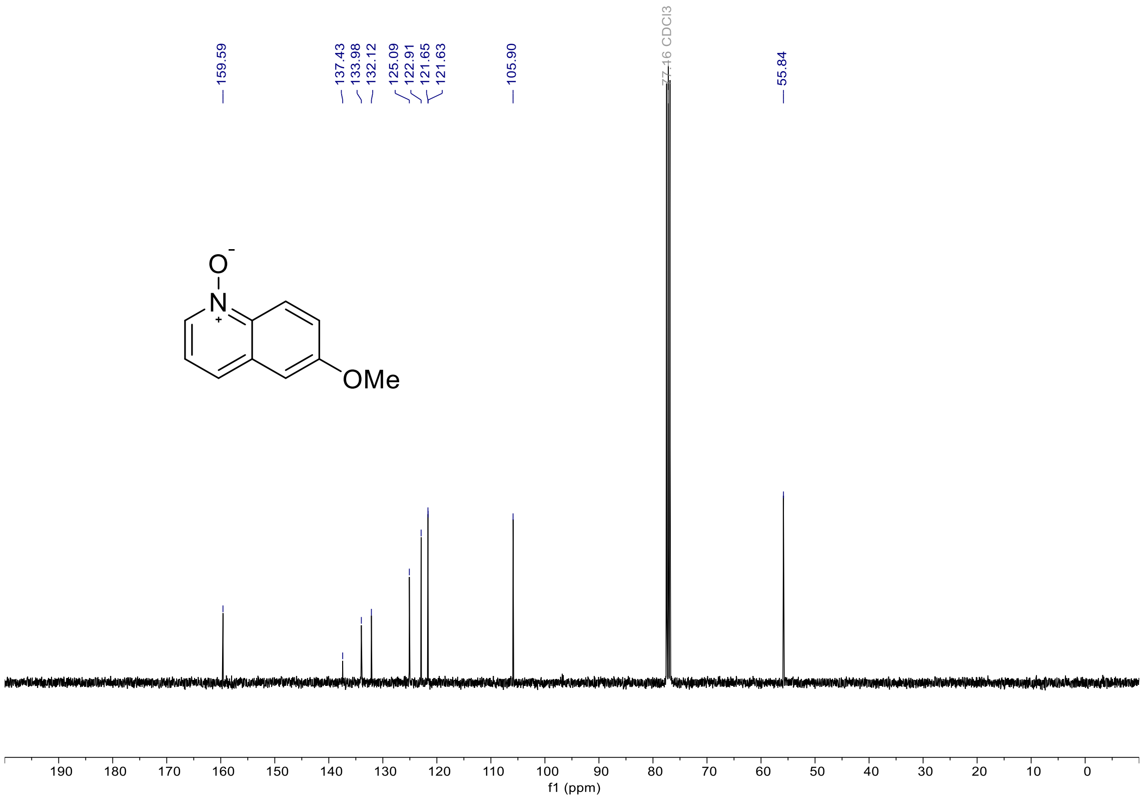

^{1}H NMR (400 MHz, $CDCl_3$) (**2x**)

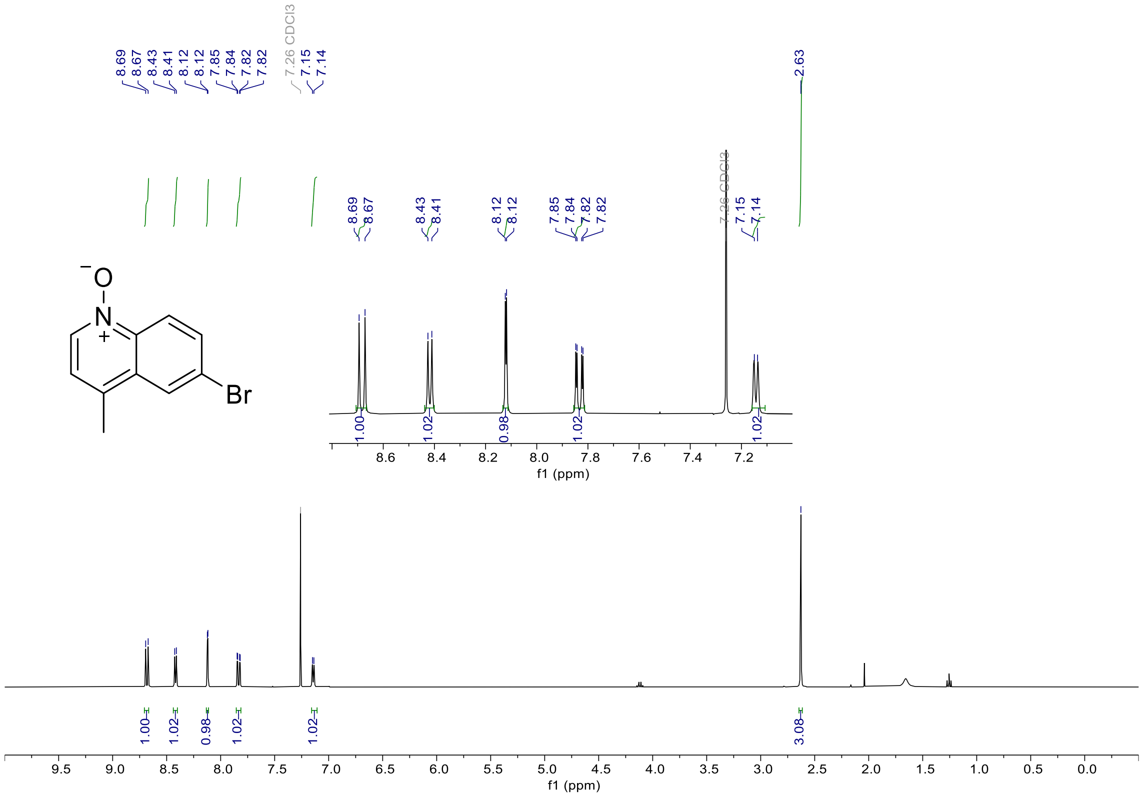

^{13}C NMR (101 MHz, $CDCl_3$) (**2x**)

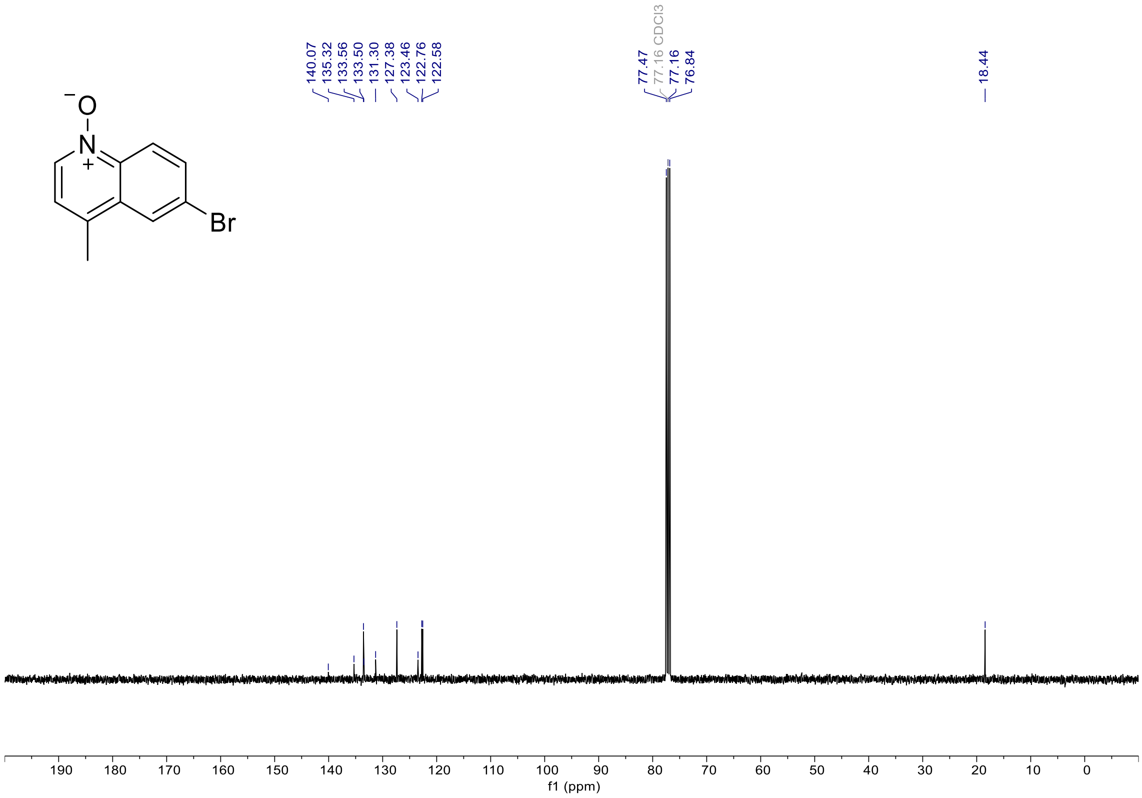

^{1}H NMR (400 MHz, $CDCl_3$) (**2y**)

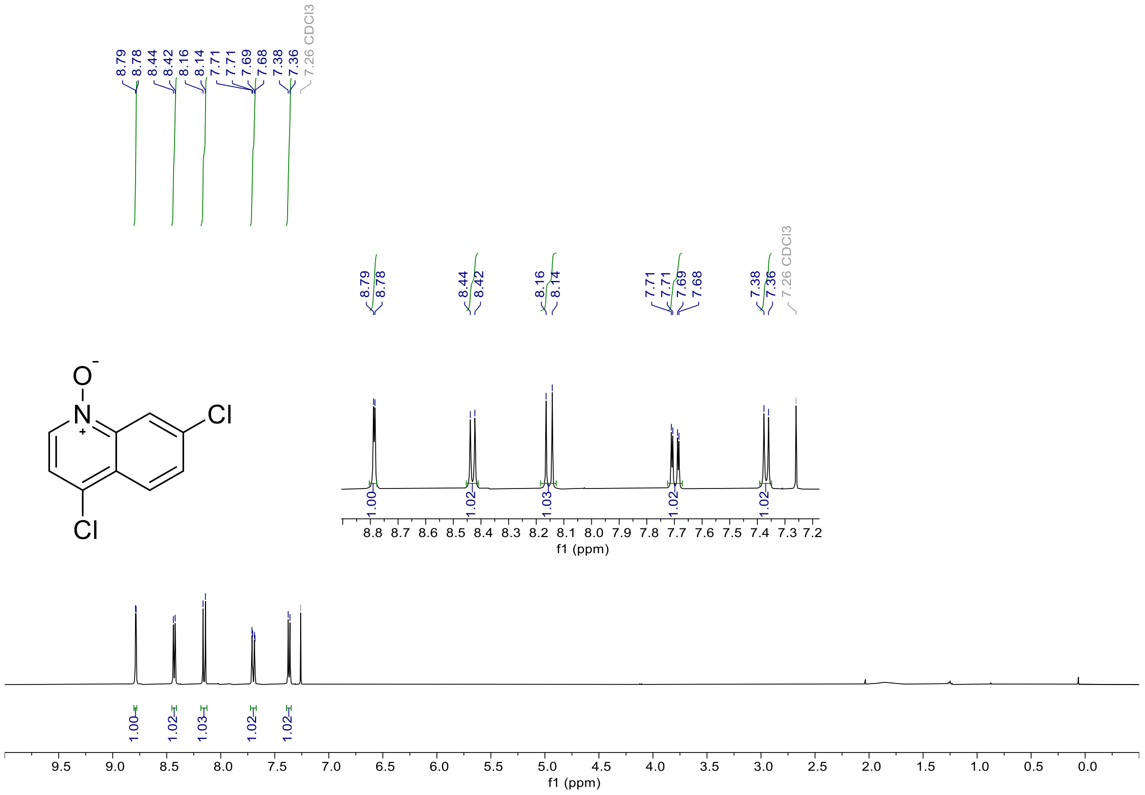

^{13}C NMR (101 MHz, $CDCl_3$) (**2y**)

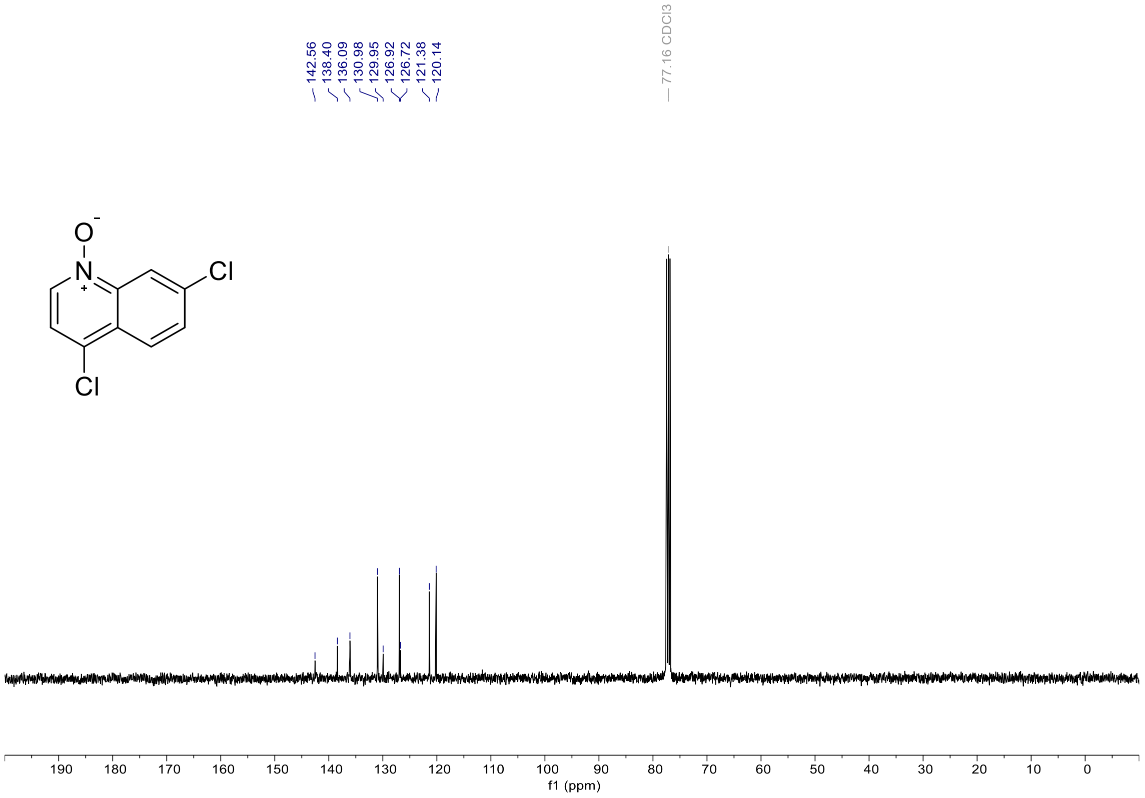

^{1}H NMR (400 MHz, $CDCl_3$) (**3a**)

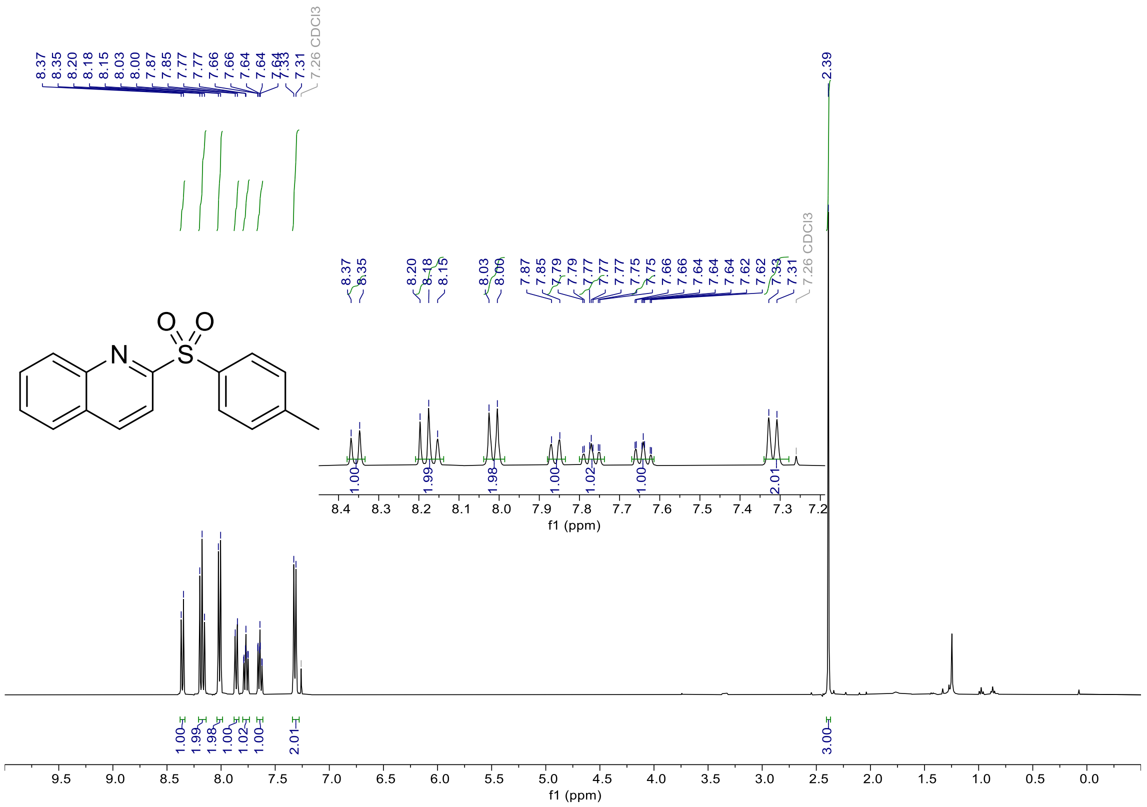

^{13}C NMR (101 MHz, $CDCl_3$) (**3a**)

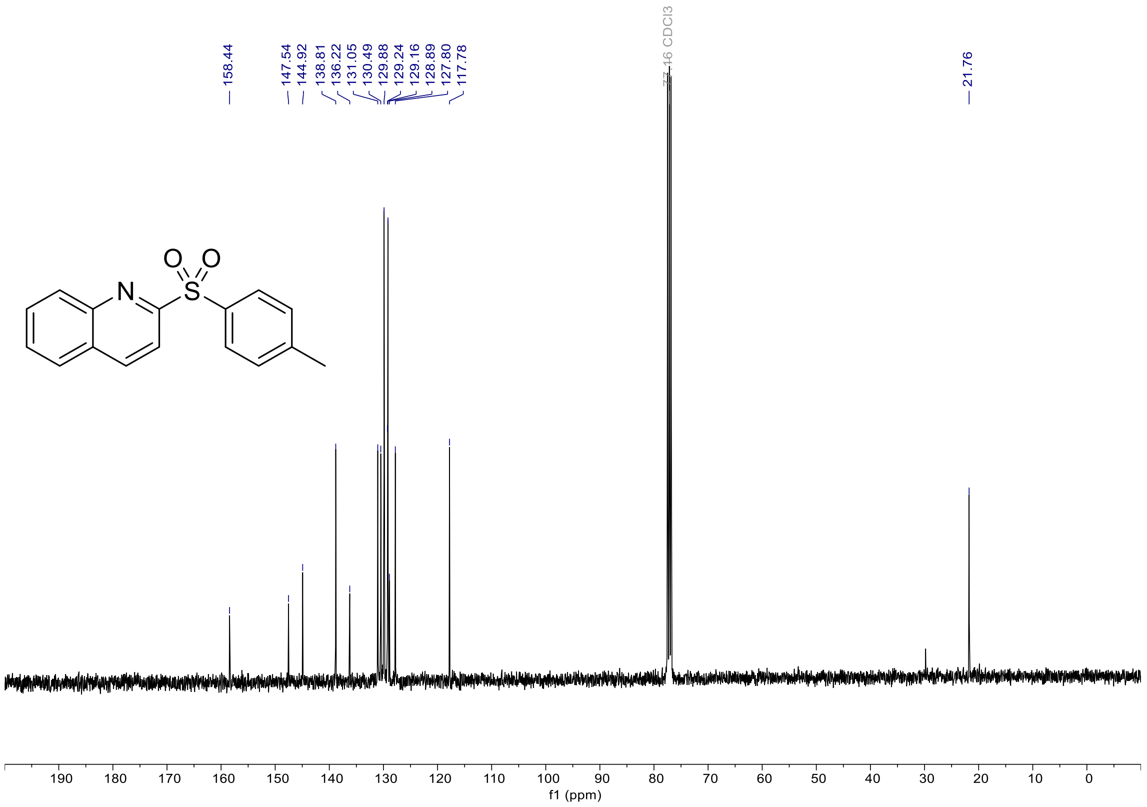

^{1}H NMR (400 MHz, $CDCl_3$) (**3b**)

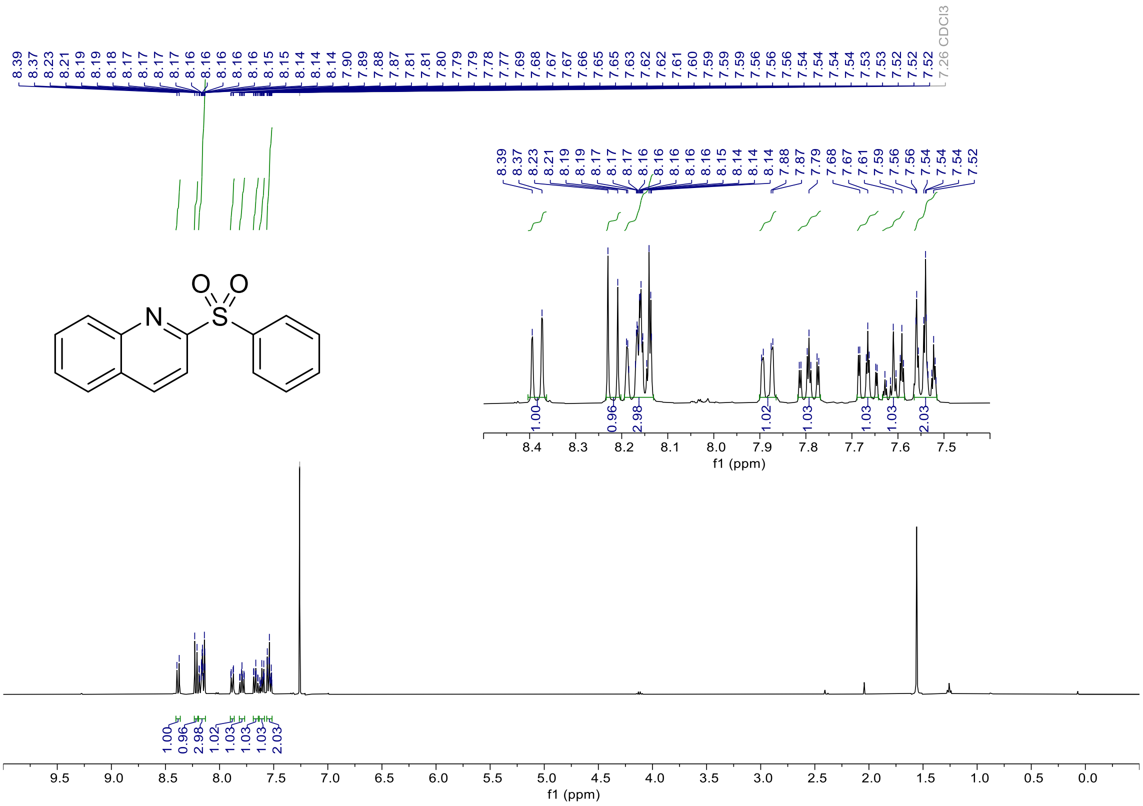

^{13}C NMR (101 MHz, $CDCl_3$) (**3b**)

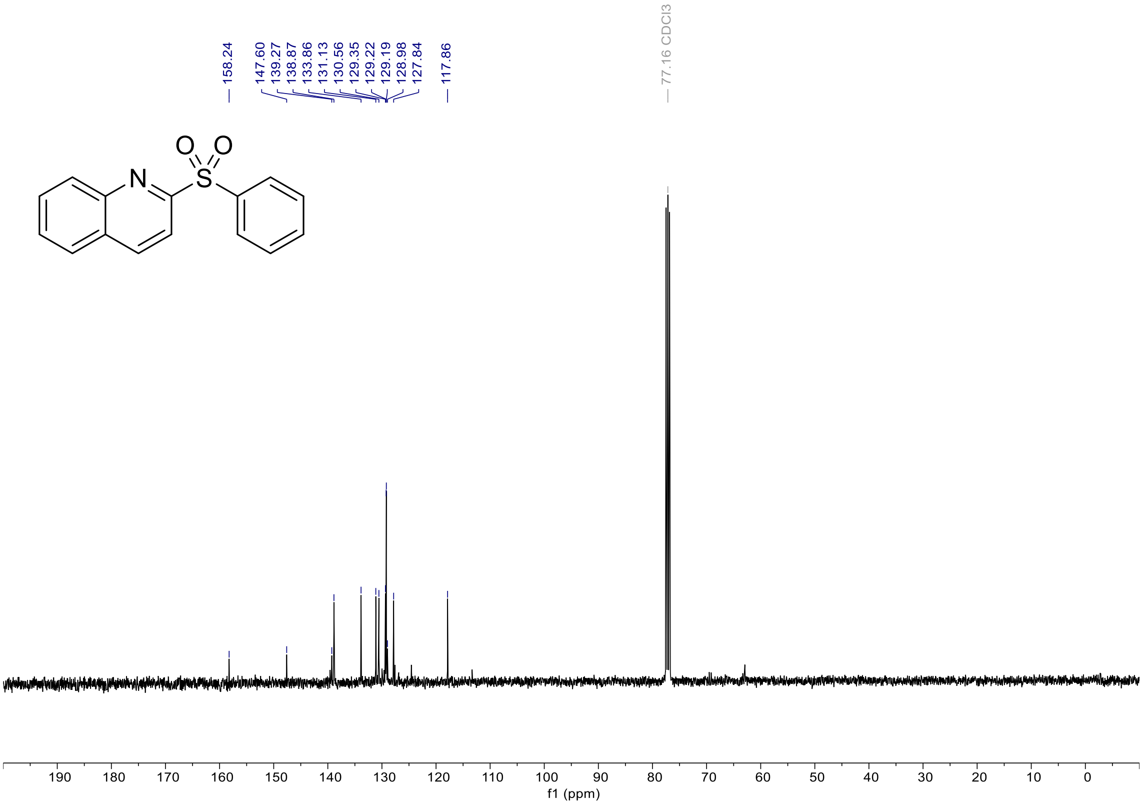

^{1}H NMR (400 MHz, $CDCl_3$) (**3c**)

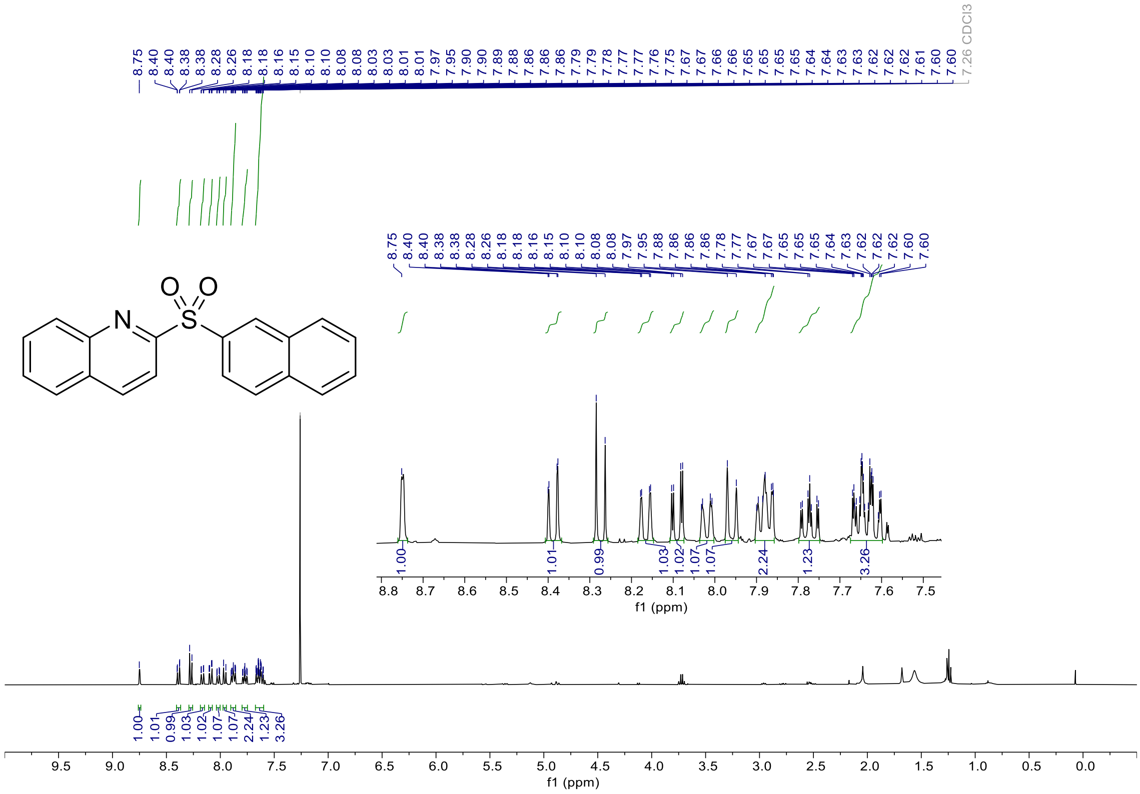

^{13}C NMR (101 MHz, $CDCl_3$) (**3c**)

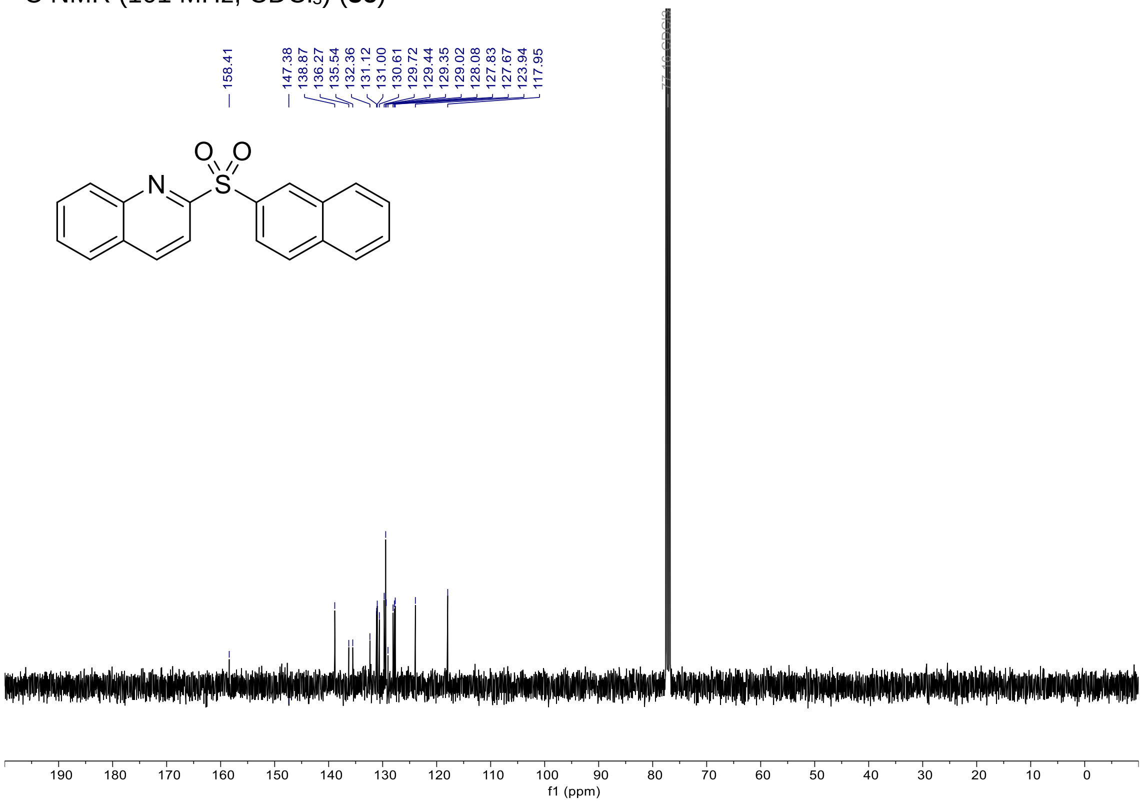

^1H NMR (400 MHz, $CDCl_3$) (**3d**)

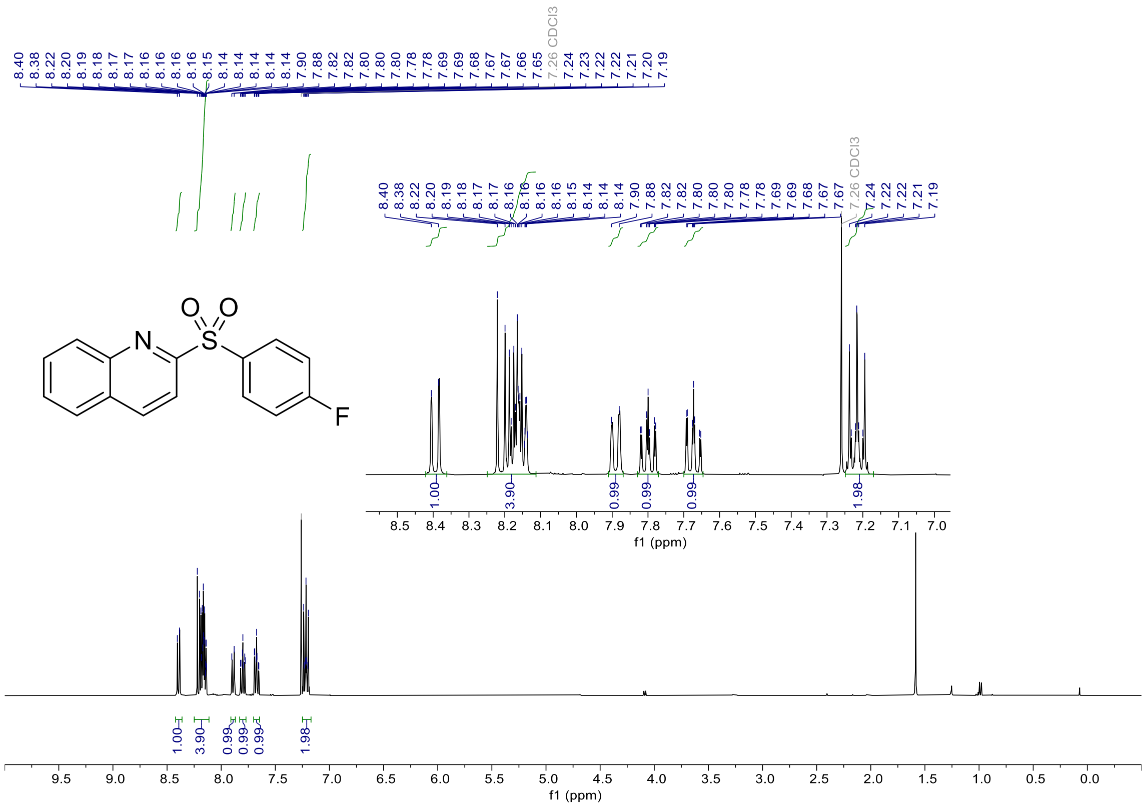

^{13}C NMR (101 MHz, $CDCl_3$) (**3d**)

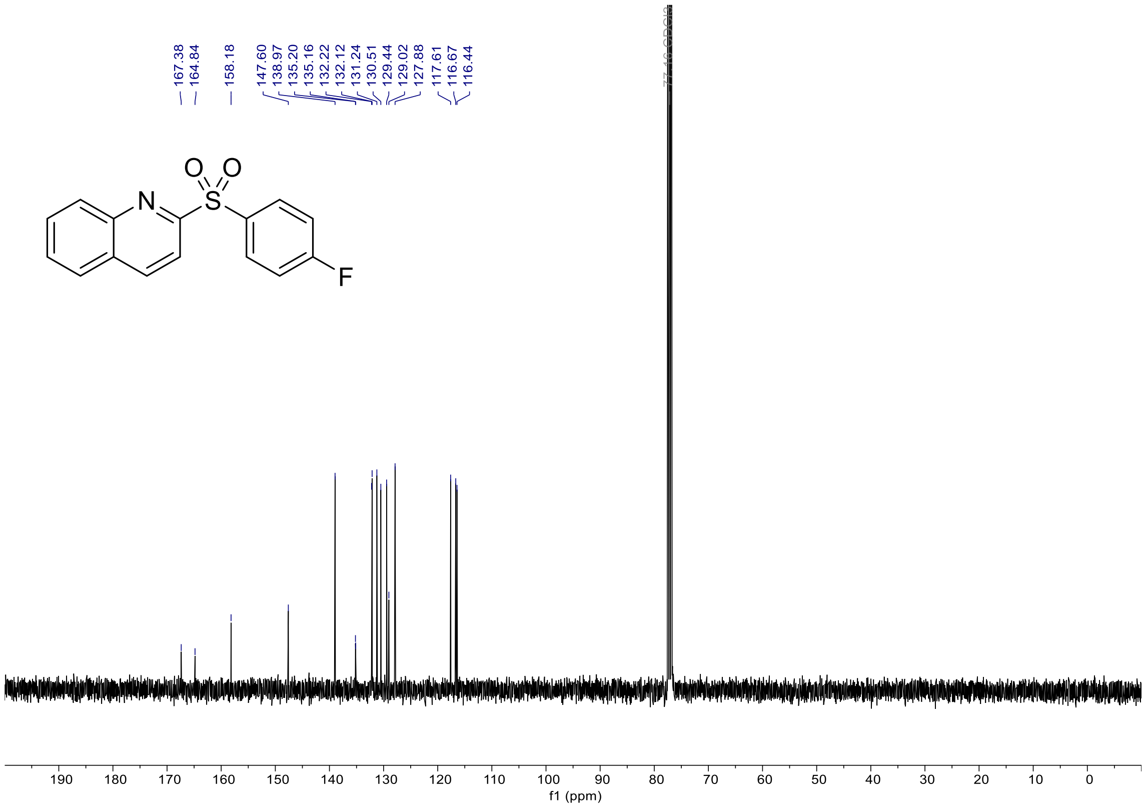

^{19}F NMR (377 MHz, $CDCl_3$) (**3d**)

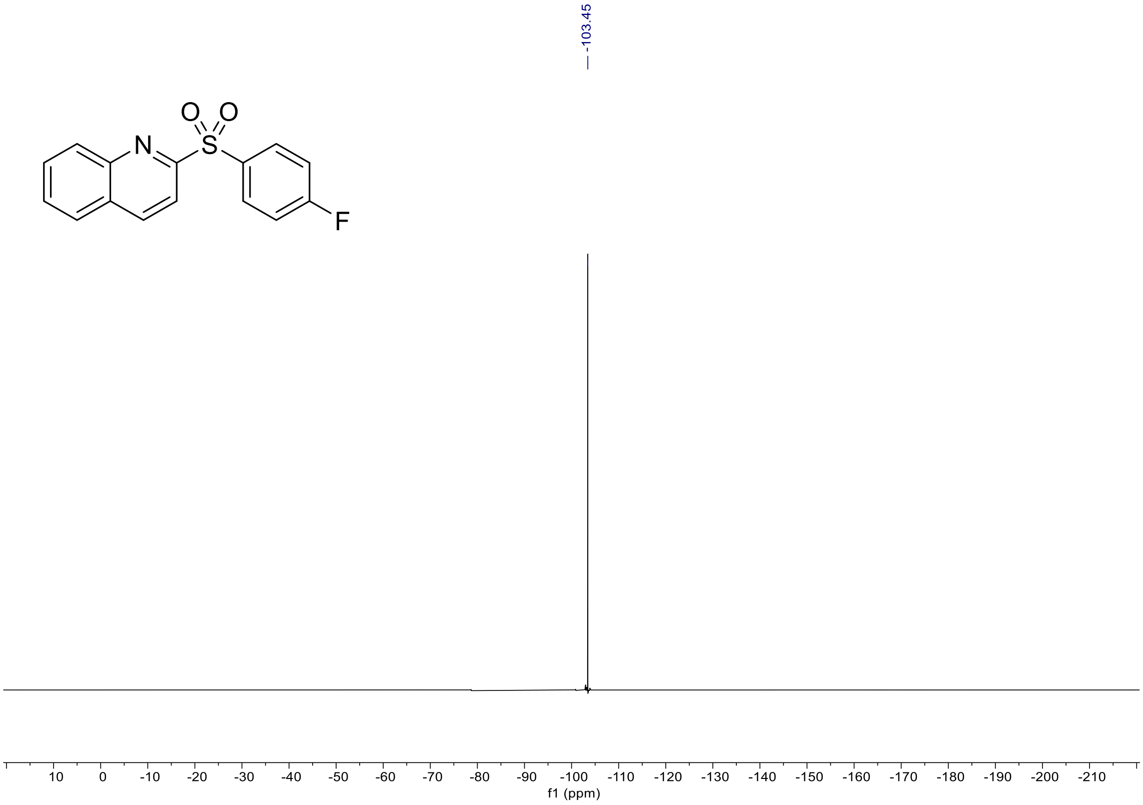

^{1}H NMR (400 MHz, $CDCl_3$) (**3e**)

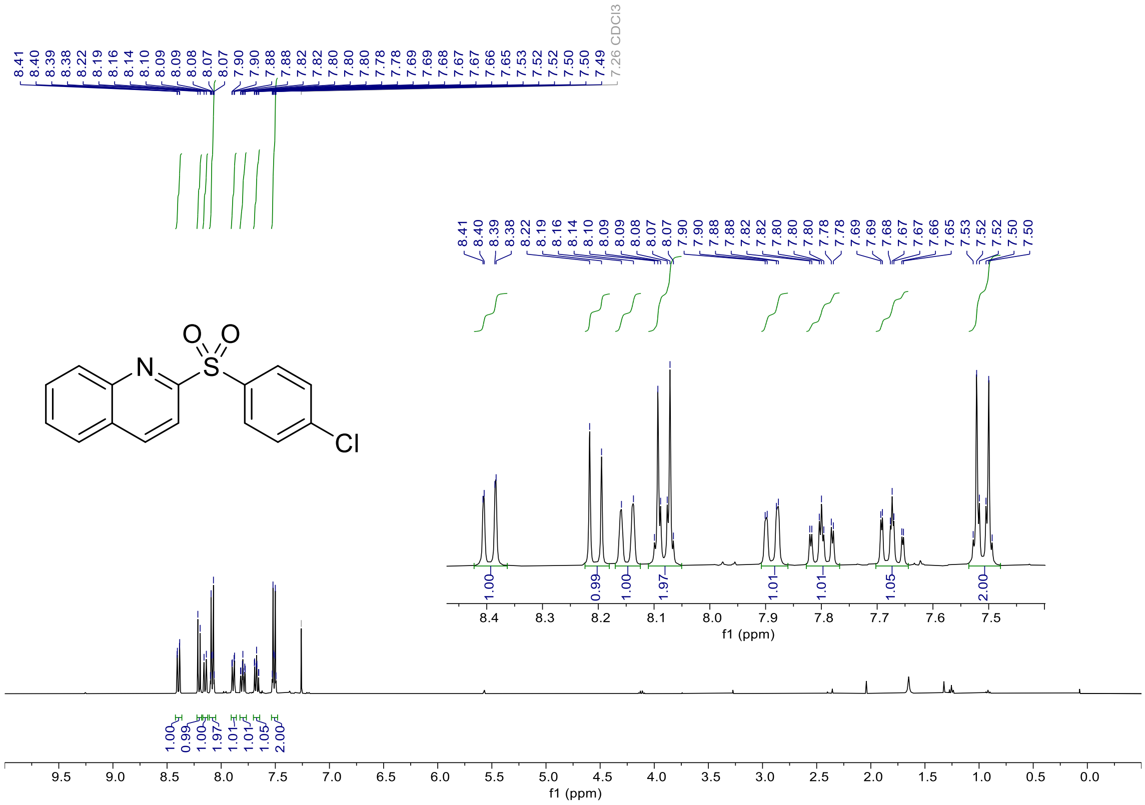

^{13}C NMR (101 MHz, $CDCl_3$) (**3e**)

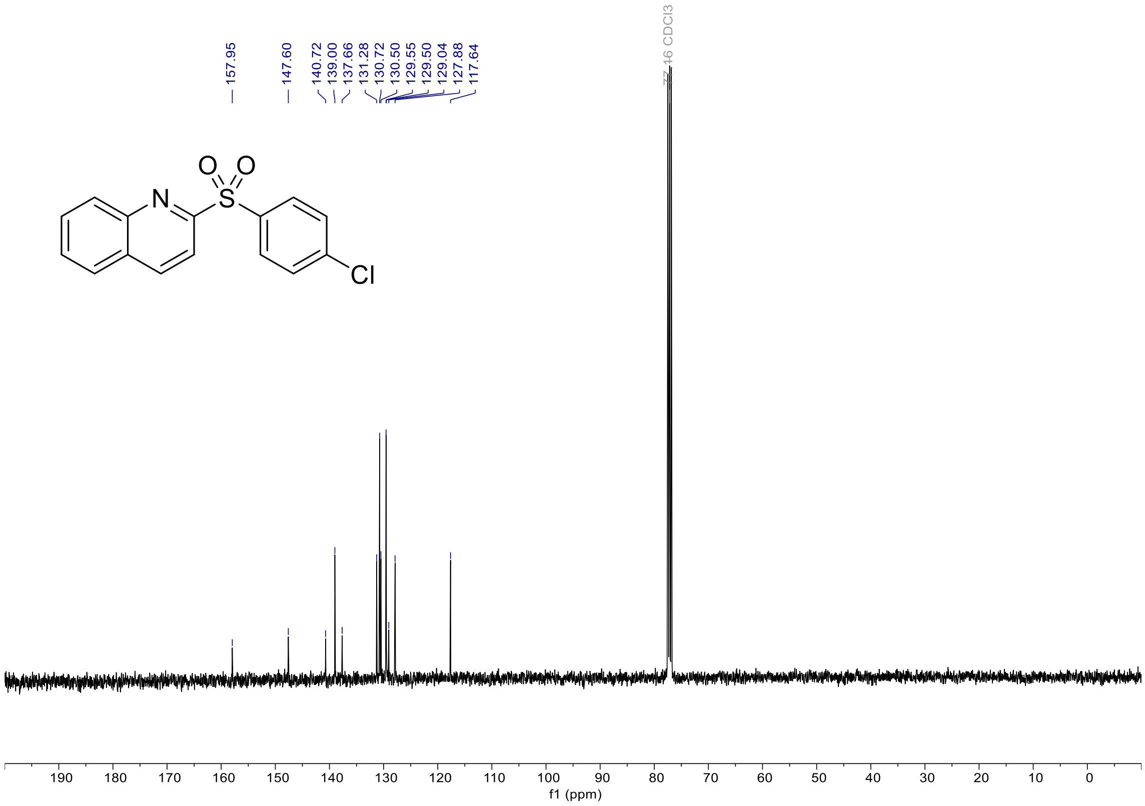

^{1}H NMR (400 MHz, $CDCl_3$) (**3f**)

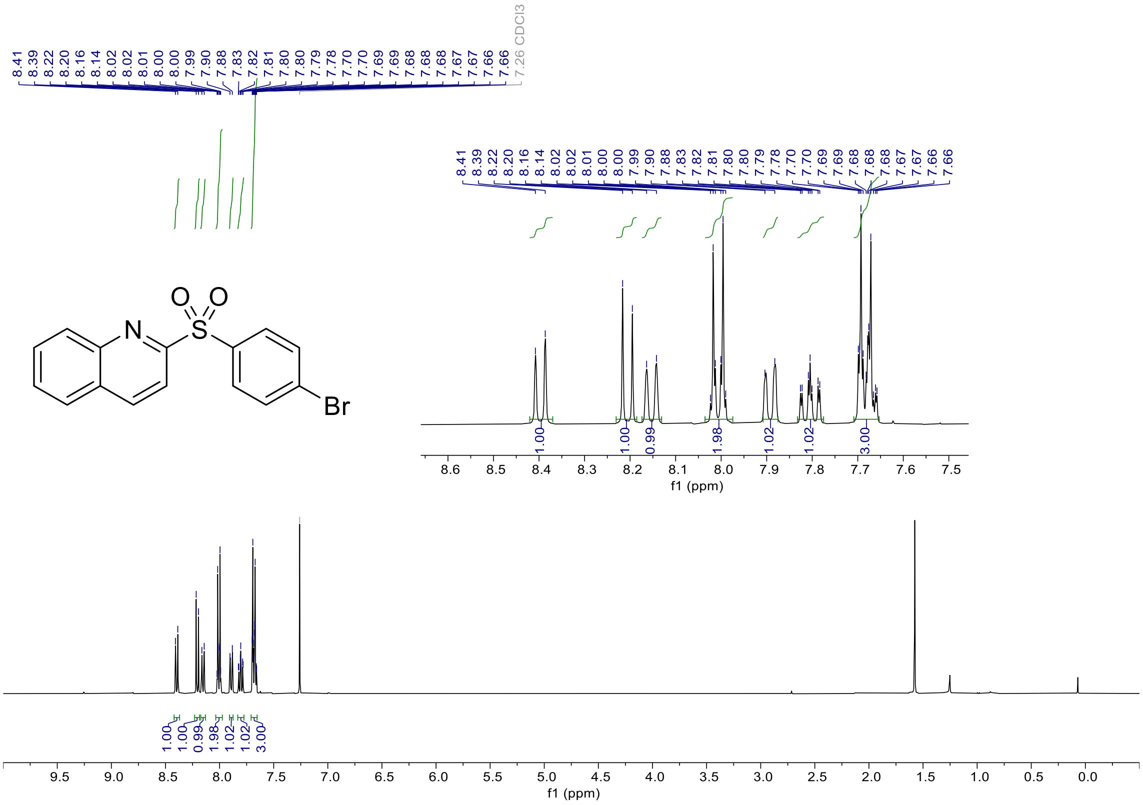

^{13}C NMR (101 MHz, $CDCl_3$) (**3f**)

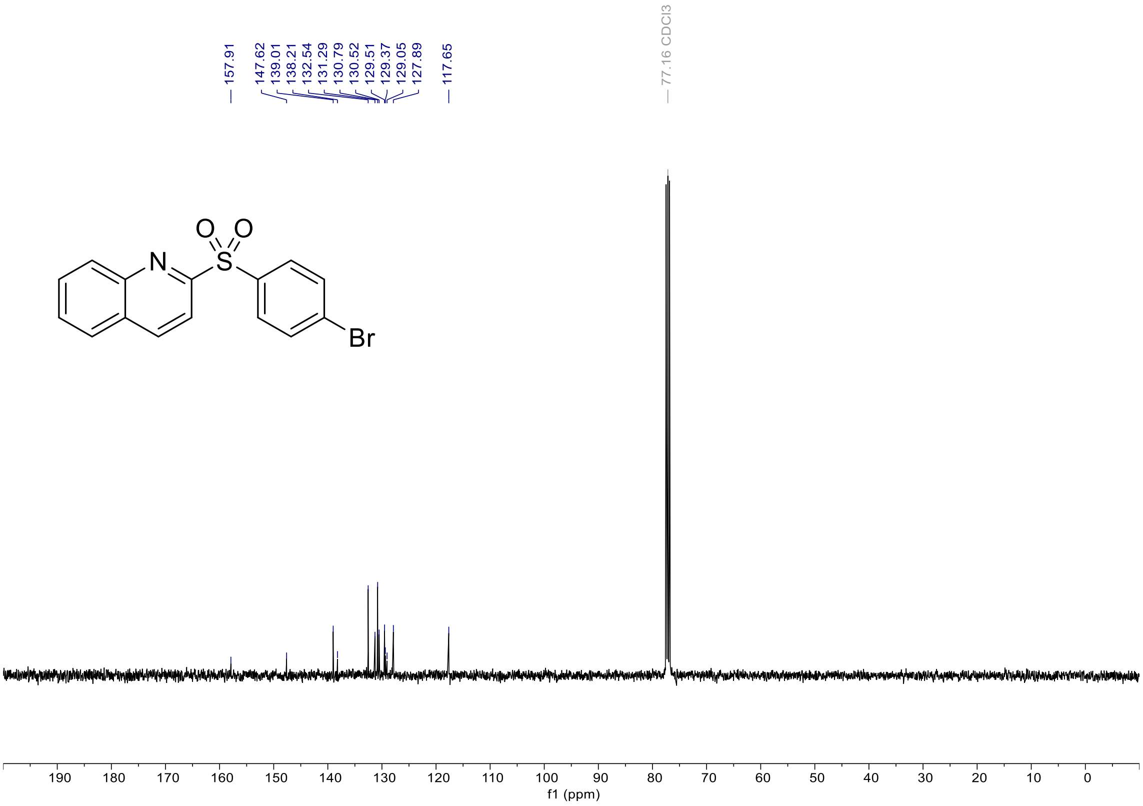

^{1}H NMR (400 MHz, $CDCl_3$) (**3g**)

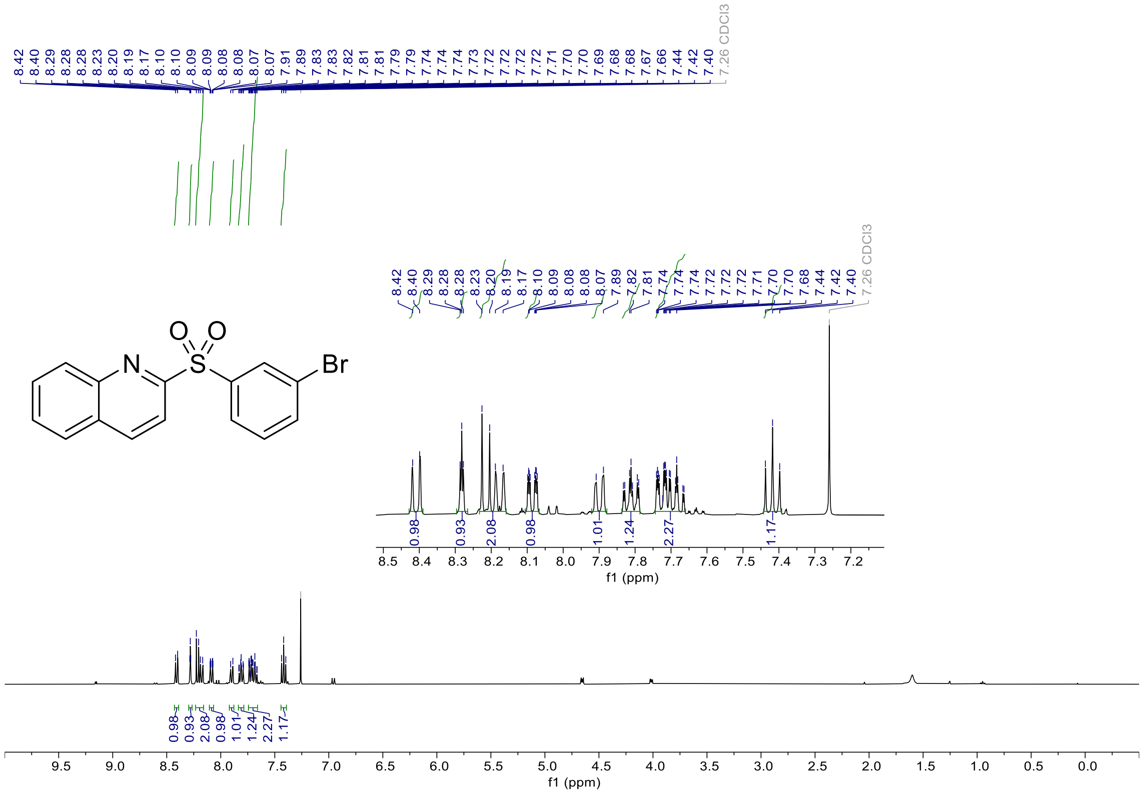

^{13}C NMR (101 MHz, $CDCl_3$) (**3g**)

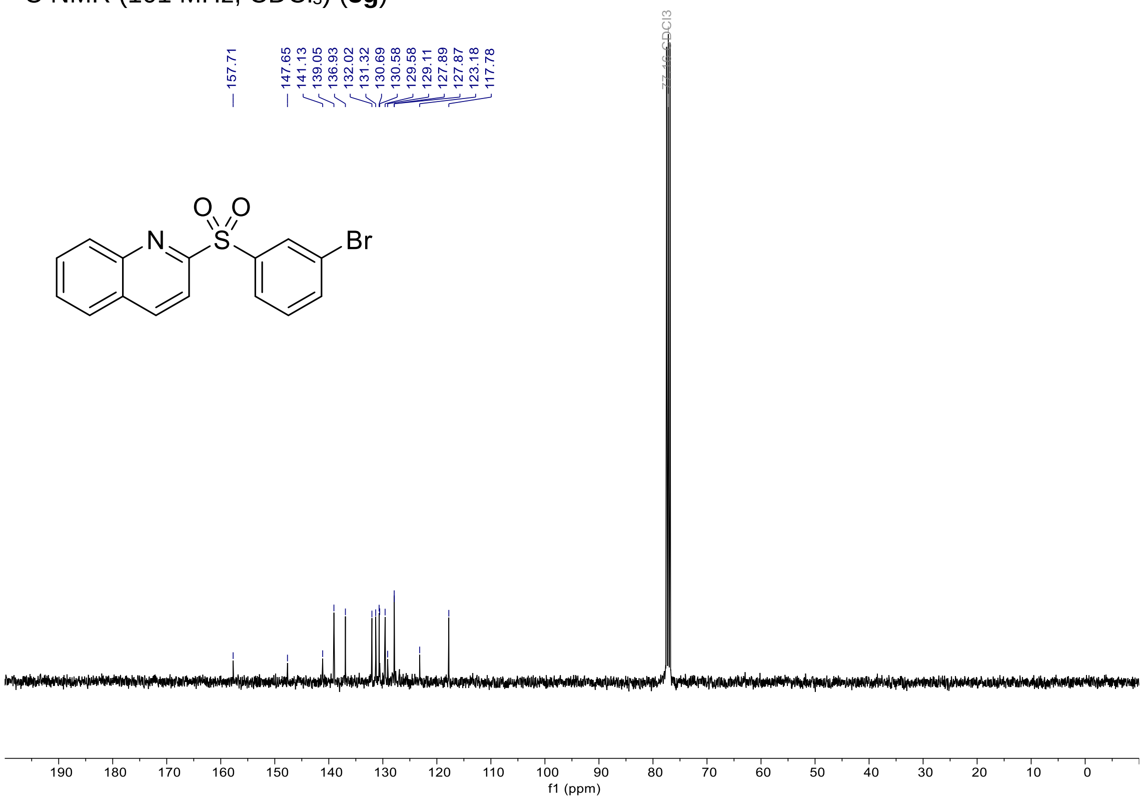

^{1}H NMR (400 MHz, $CDCl_3$) (**3h**)

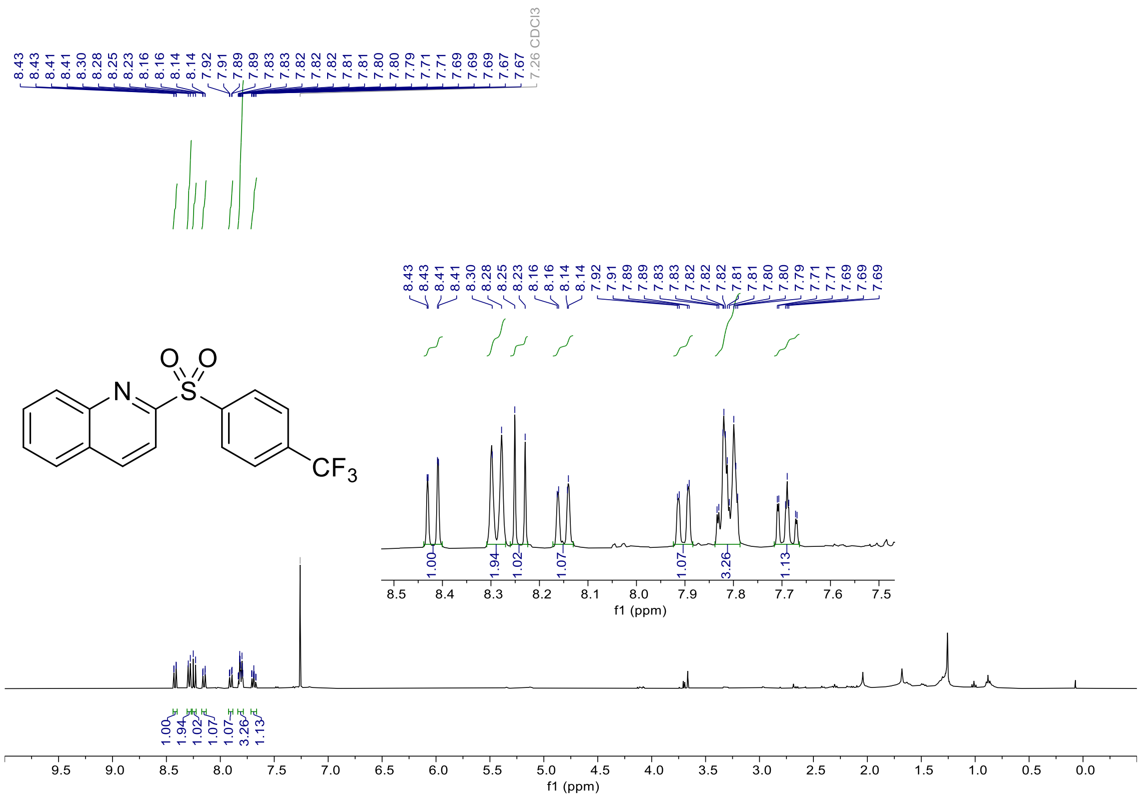

^{13}C NMR (101 MHz, $CDCl_3$) (**3h**)

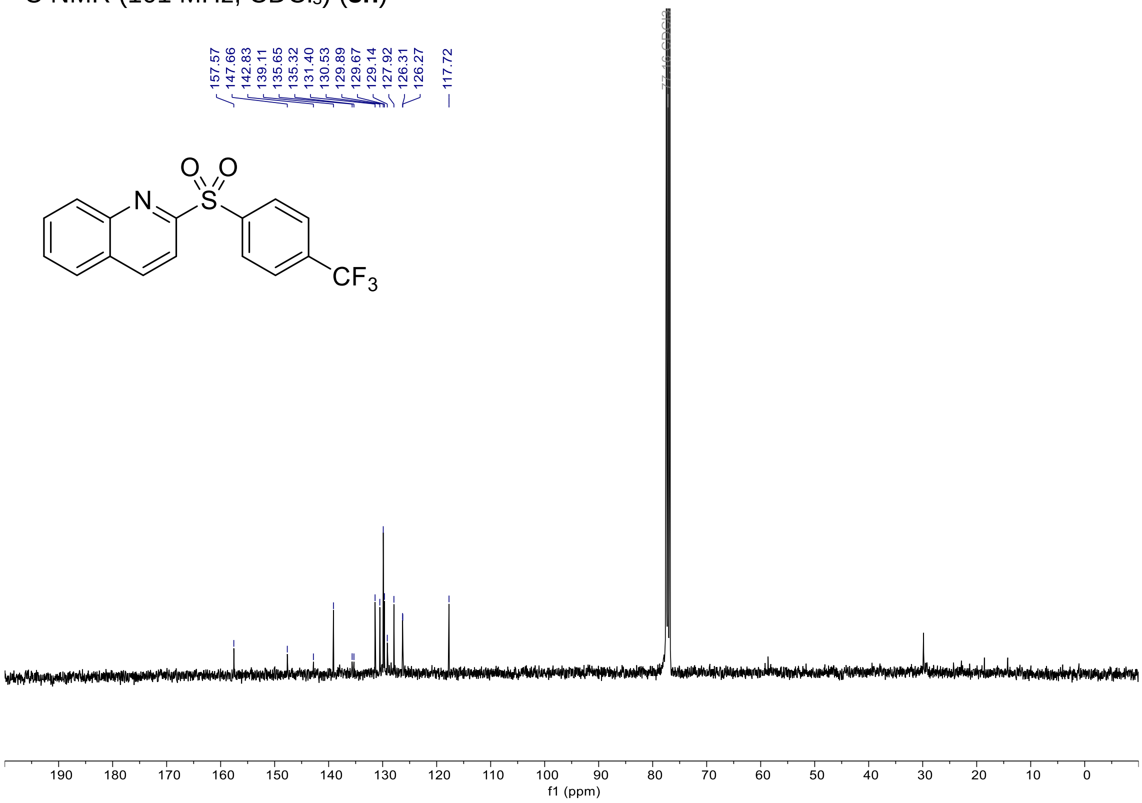

^{19}F NMR (376 MHz, $CDCl_3$) (**3h**)

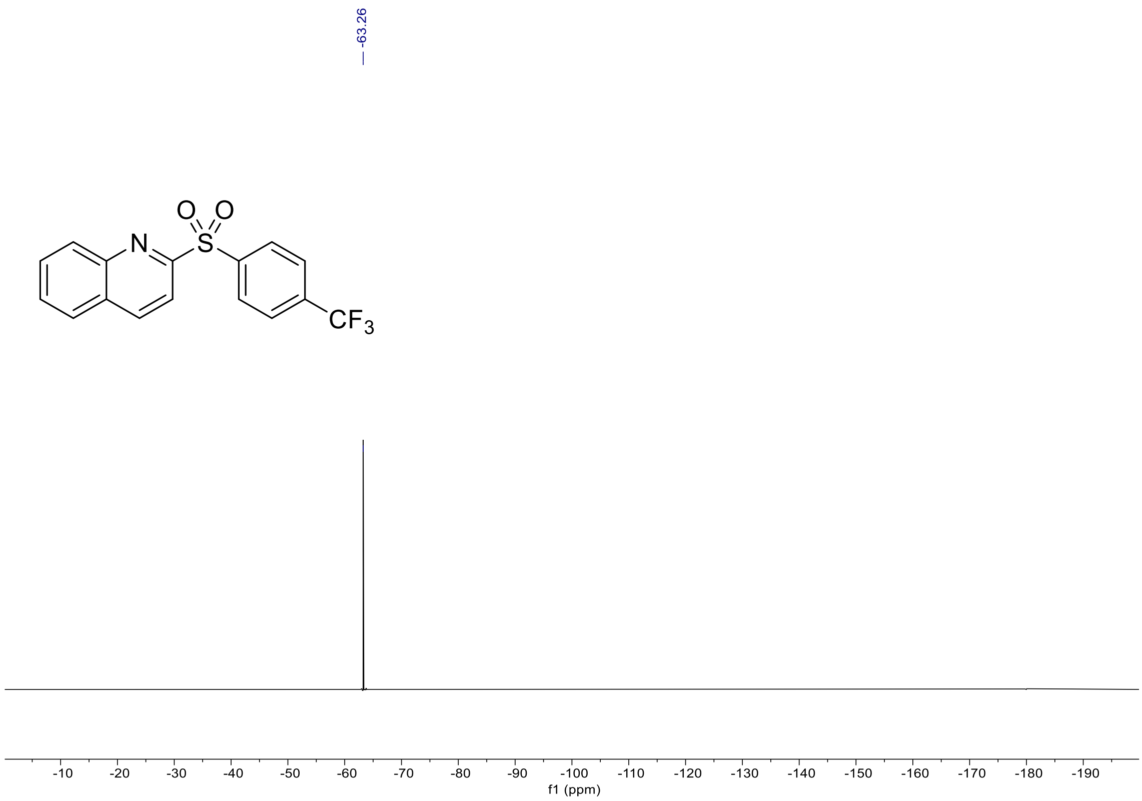

^{1}H NMR (400 MHz, $CDCl_3$) (**3i**)

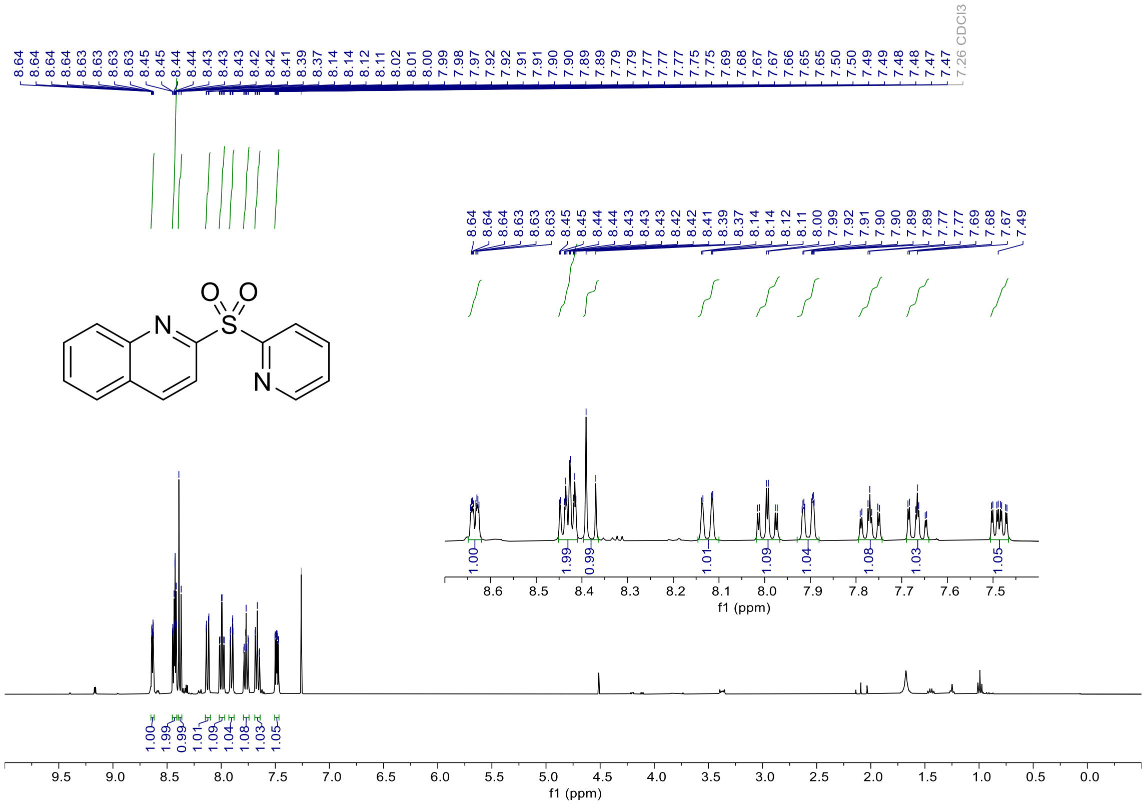

^{13}C NMR (101 MHz, $CDCl_3$) (**3i**)

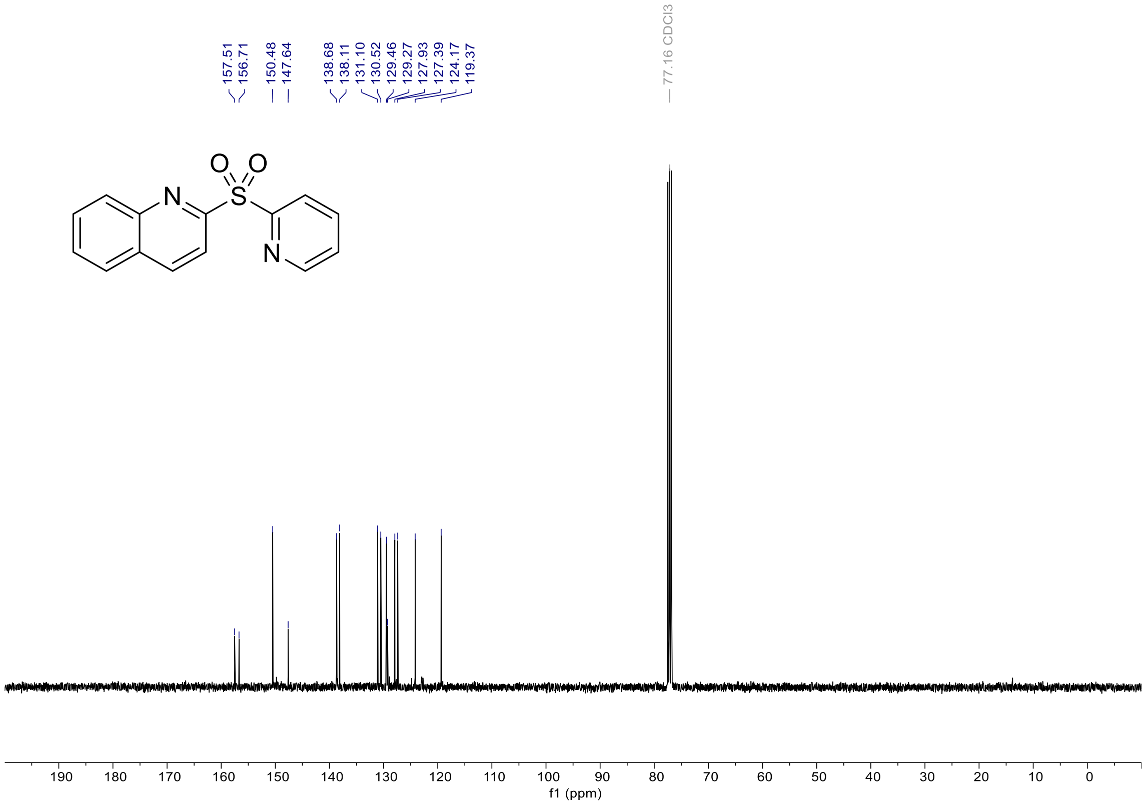

^{1}H NMR (400 MHz, $CDCl_3$) (**3j**)

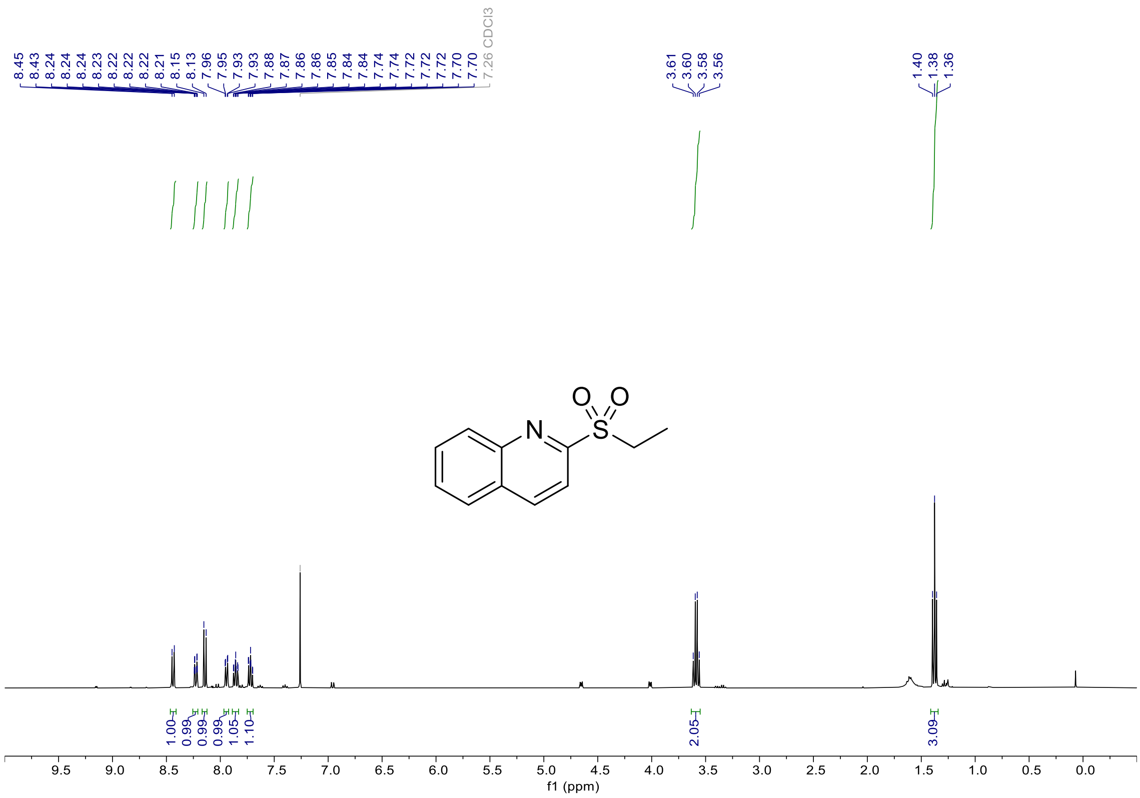

^{13}C NMR (101 MHz, $CDCl_3$) (**3j**)

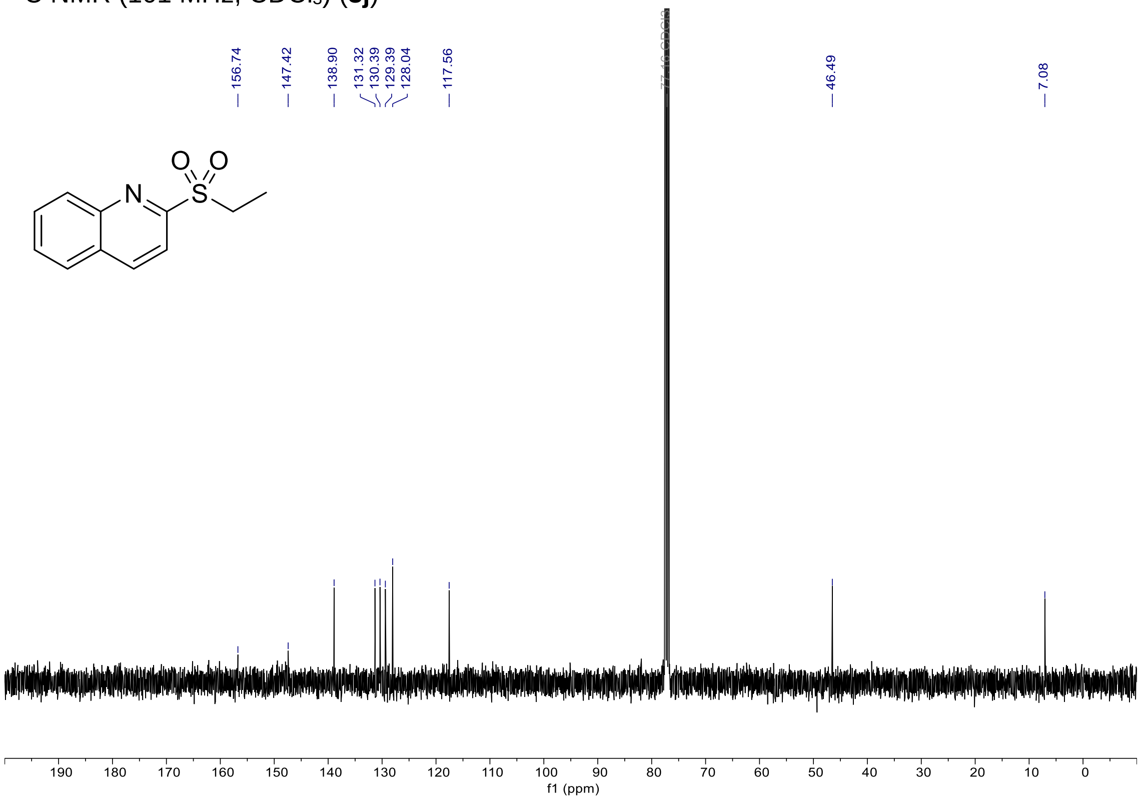

^{1}H NMR (400 MHz, $CDCl_3$) (**3k**)

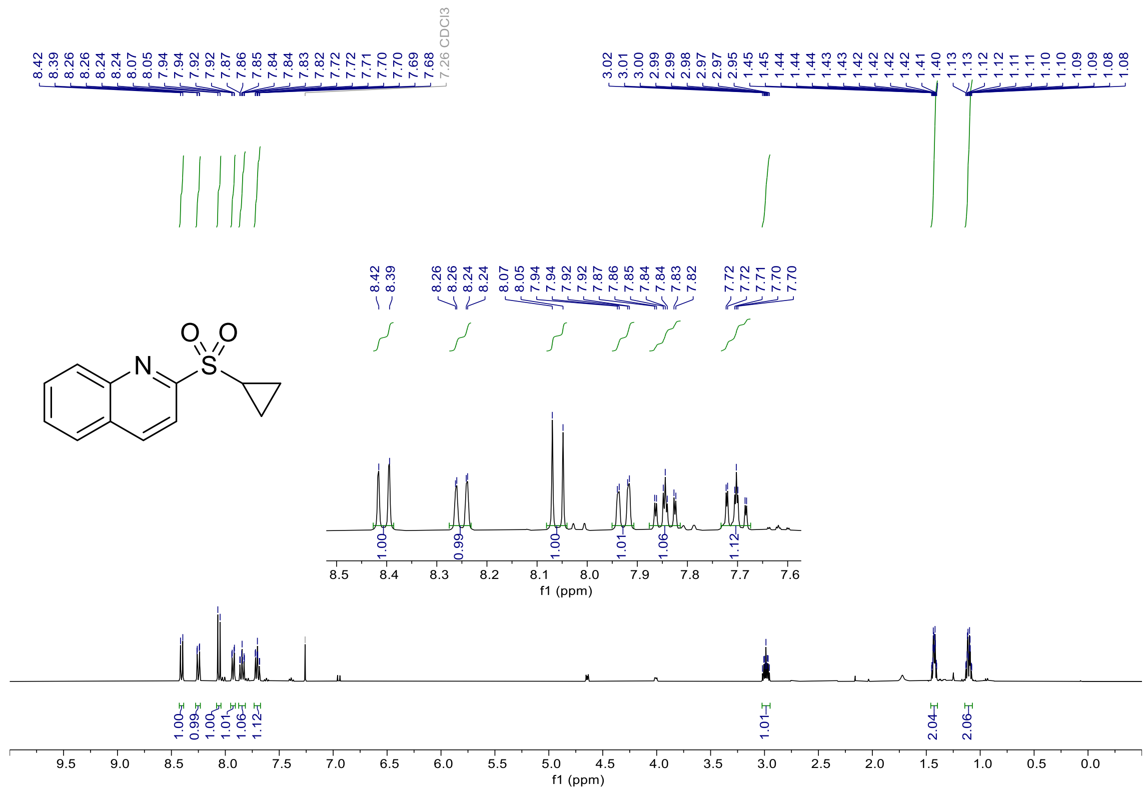

^{13}C NMR (101 MHz, $CDCl_3$) (**3k**)

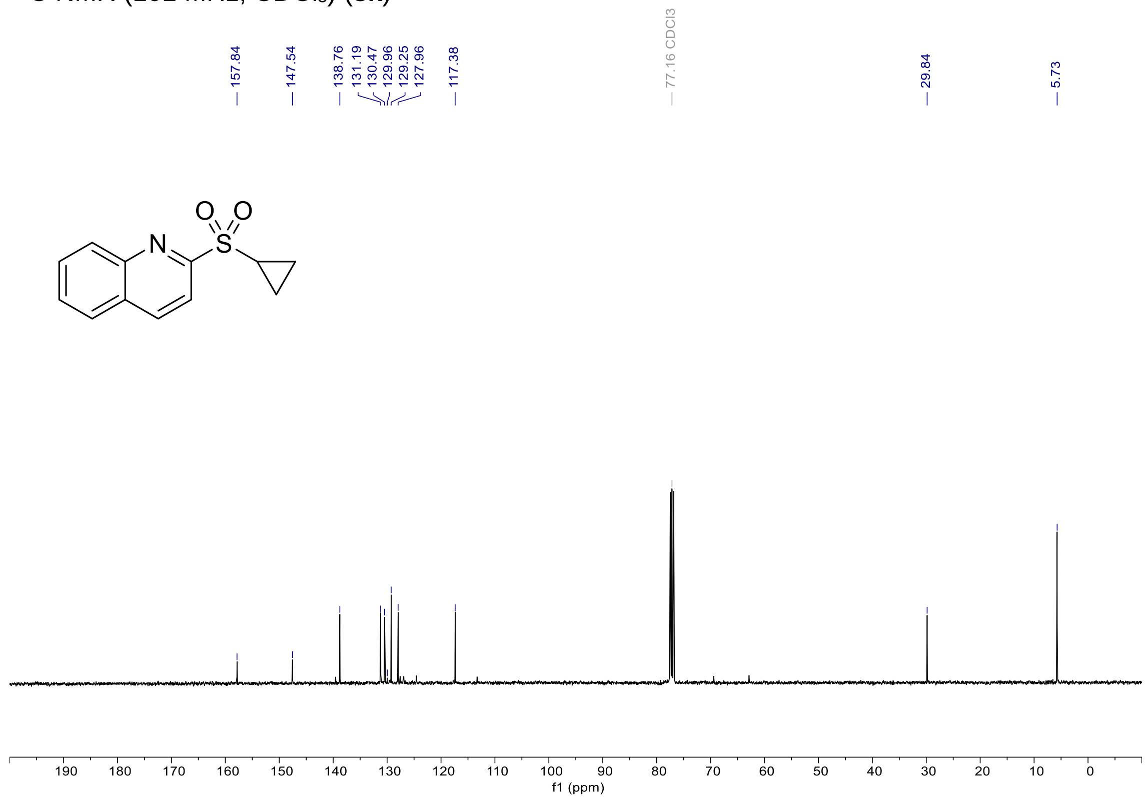

^{1}H NMR (400 MHz, CDCl$_3$) (**3l**)

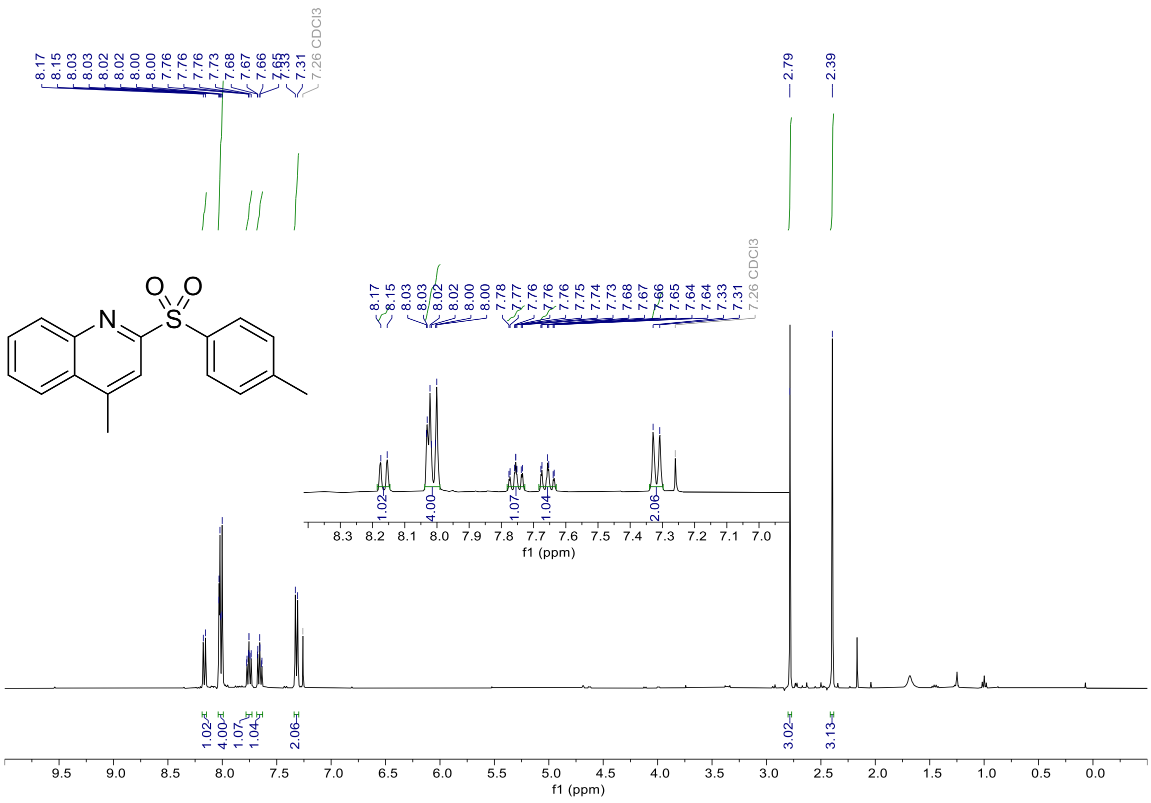

^{13}C NMR (101 MHz, CDCl$_3$) (**3l**)

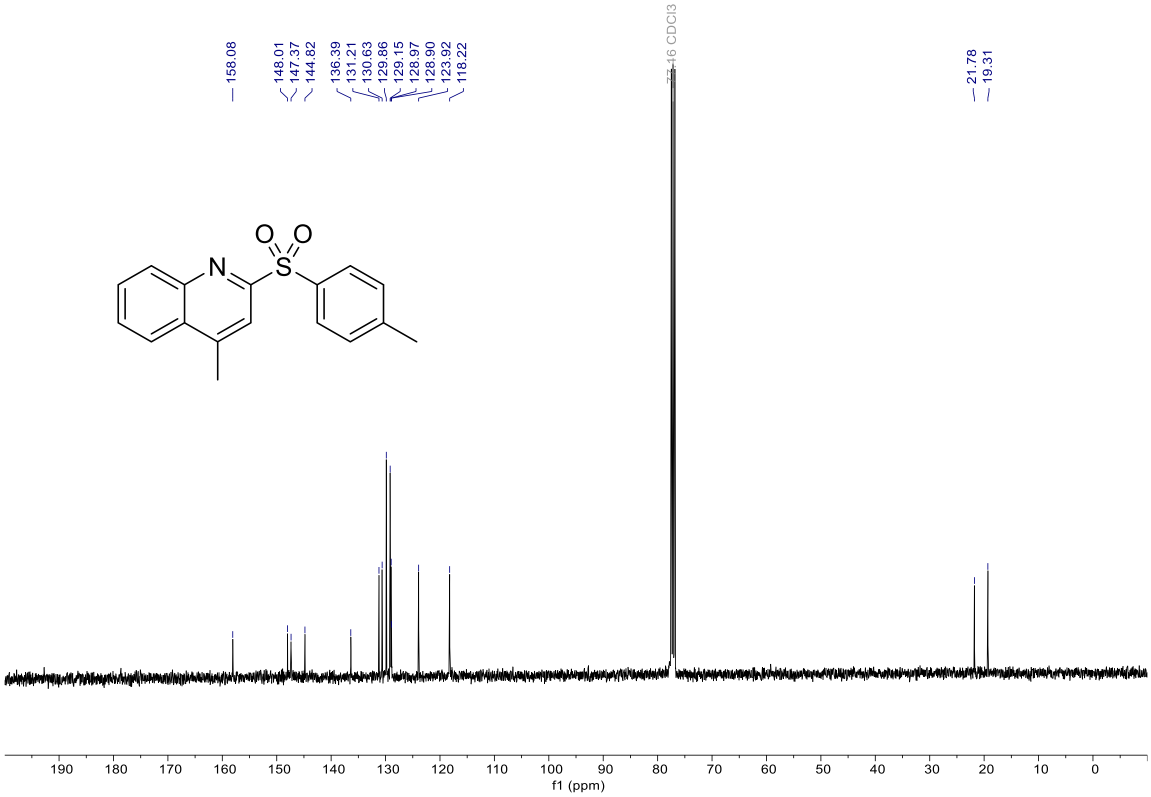

^{1}H NMR (400 MHz, $CDCl_3$) (**3m**)

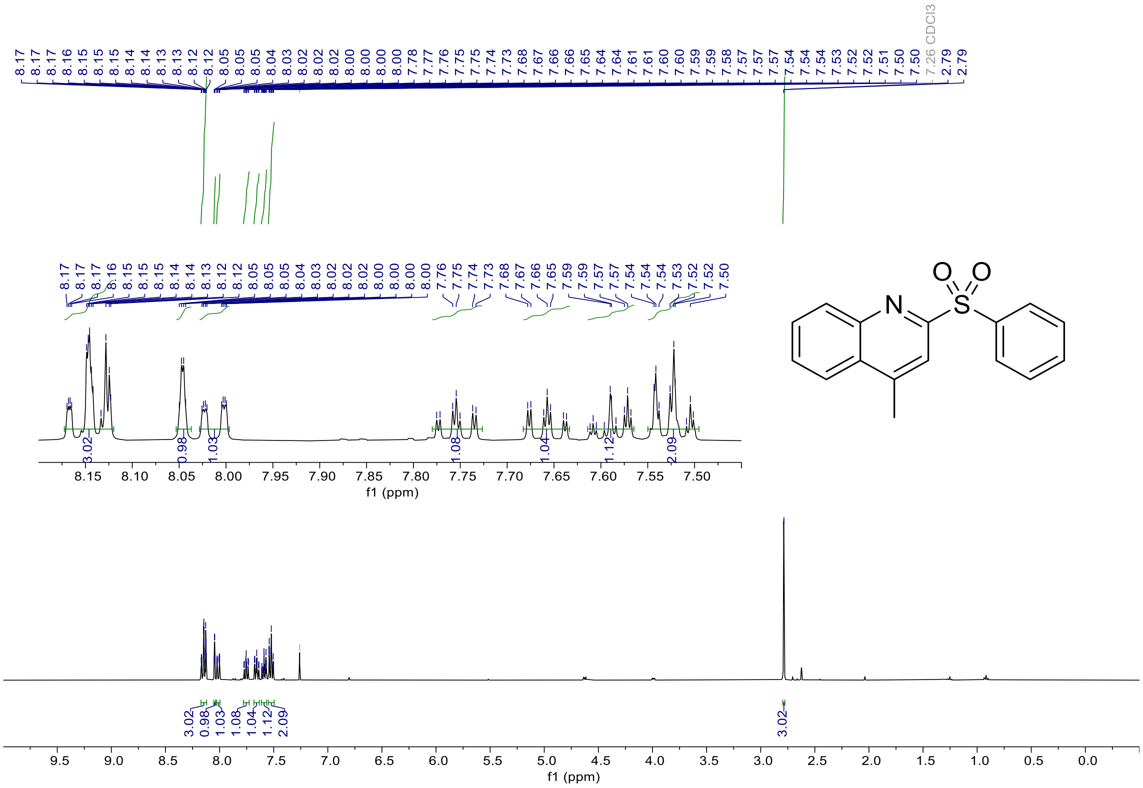

^{13}C NMR (101 MHz, $CDCl_3$) (**3m**)

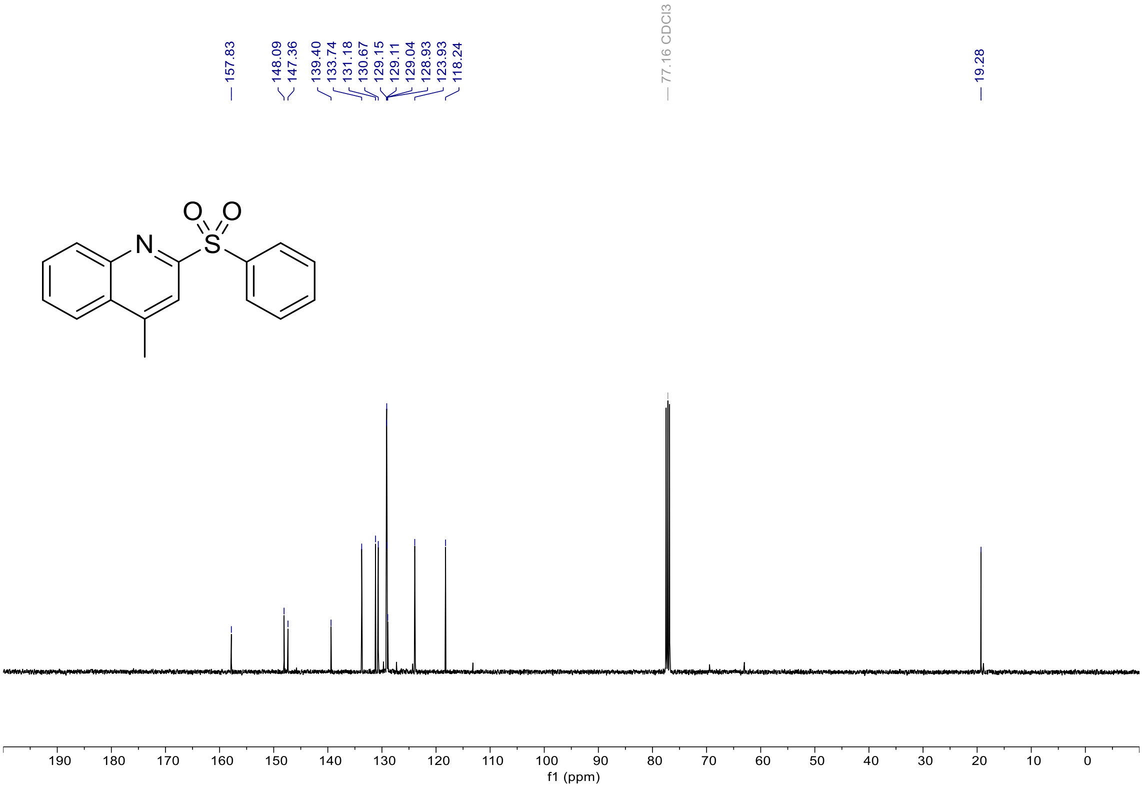

^{1}H NMR (400 MHz, CDCl$_3$) (**3n**)

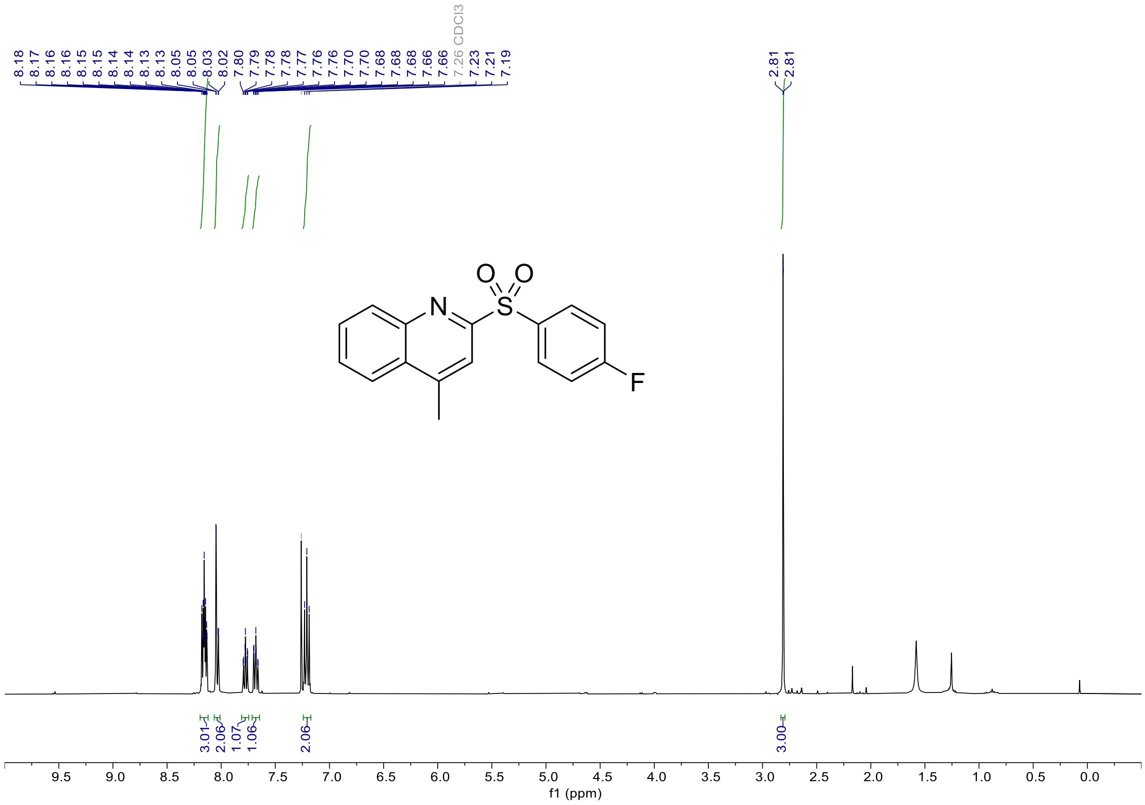

^{13}C NMR (101 MHz, CDCl$_3$) (**3n**)

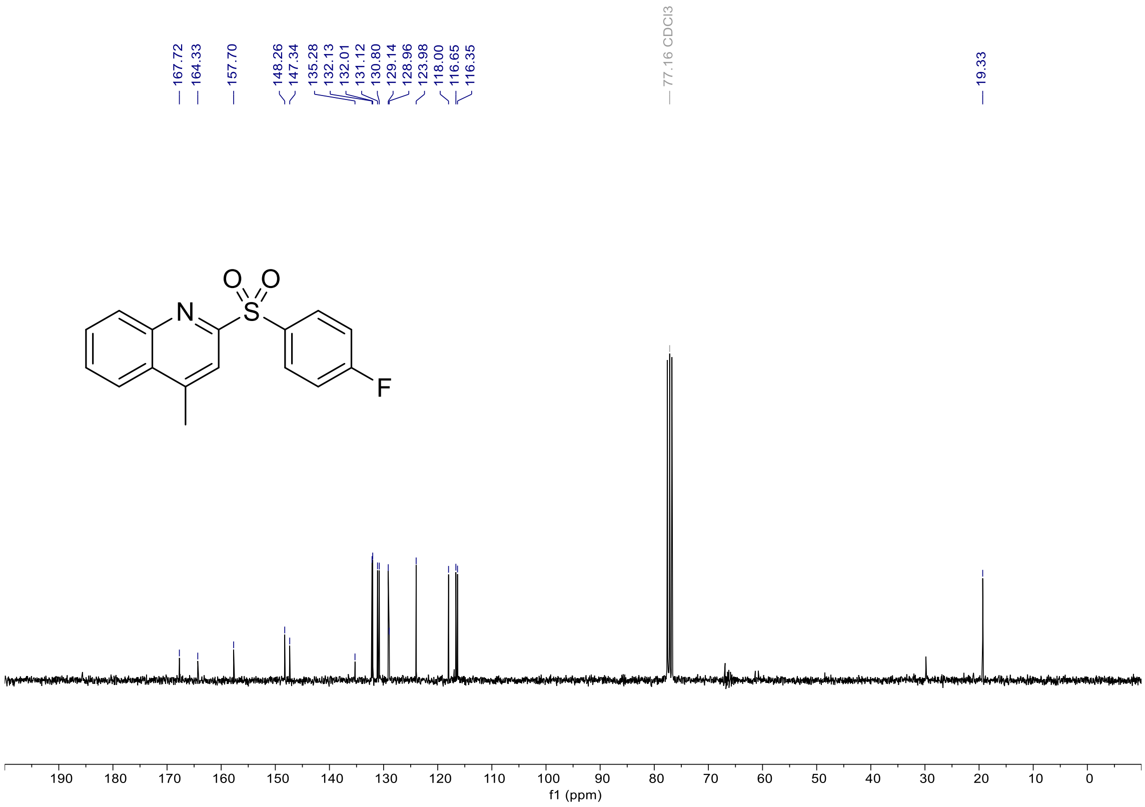

^{19}F NMR (377 MHz, $CDCl_3$) (**3n**)

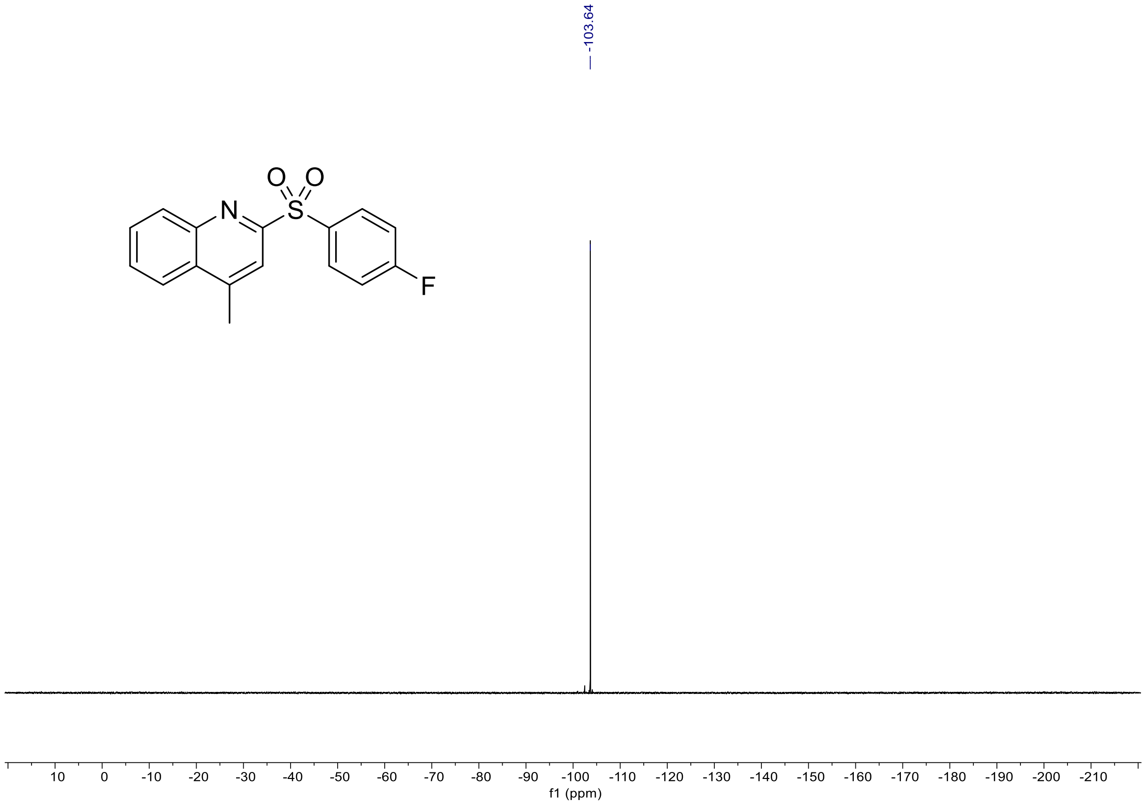

^{1}H NMR (400 MHz, CDCl$_3$) (**3o**)

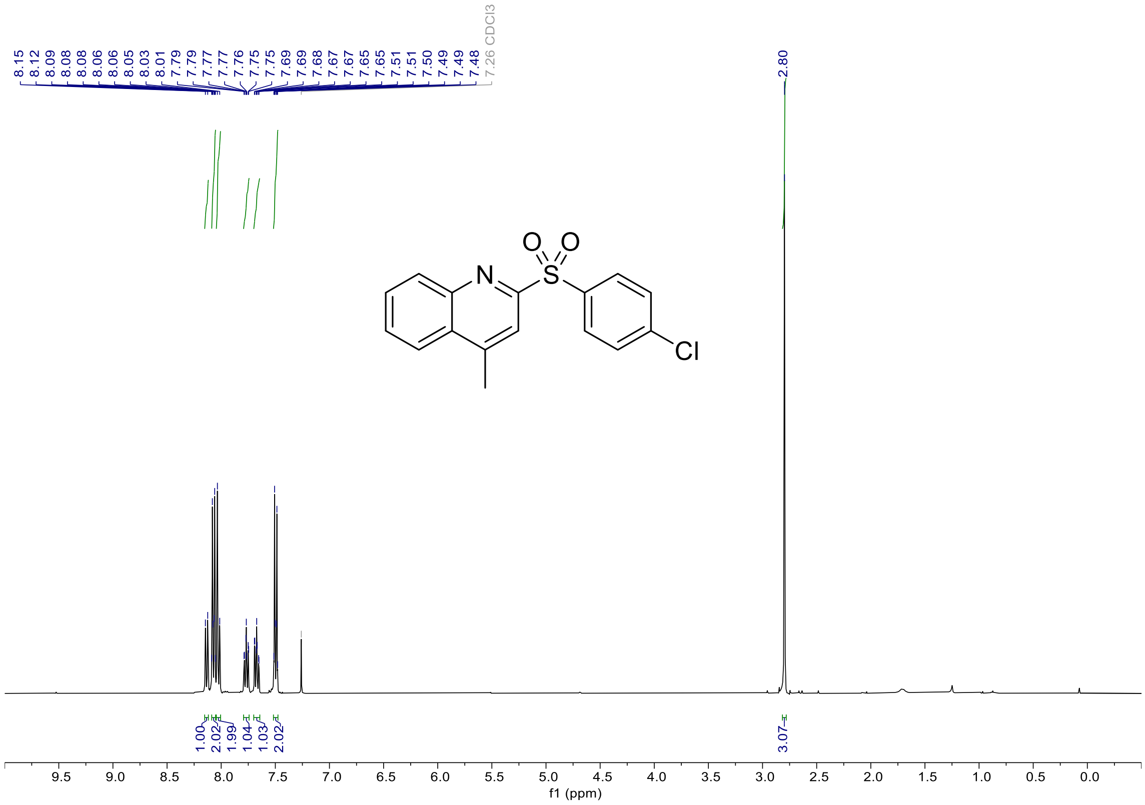

^{13}C NMR (101 MHz, CDCl$_3$) (**3o**)

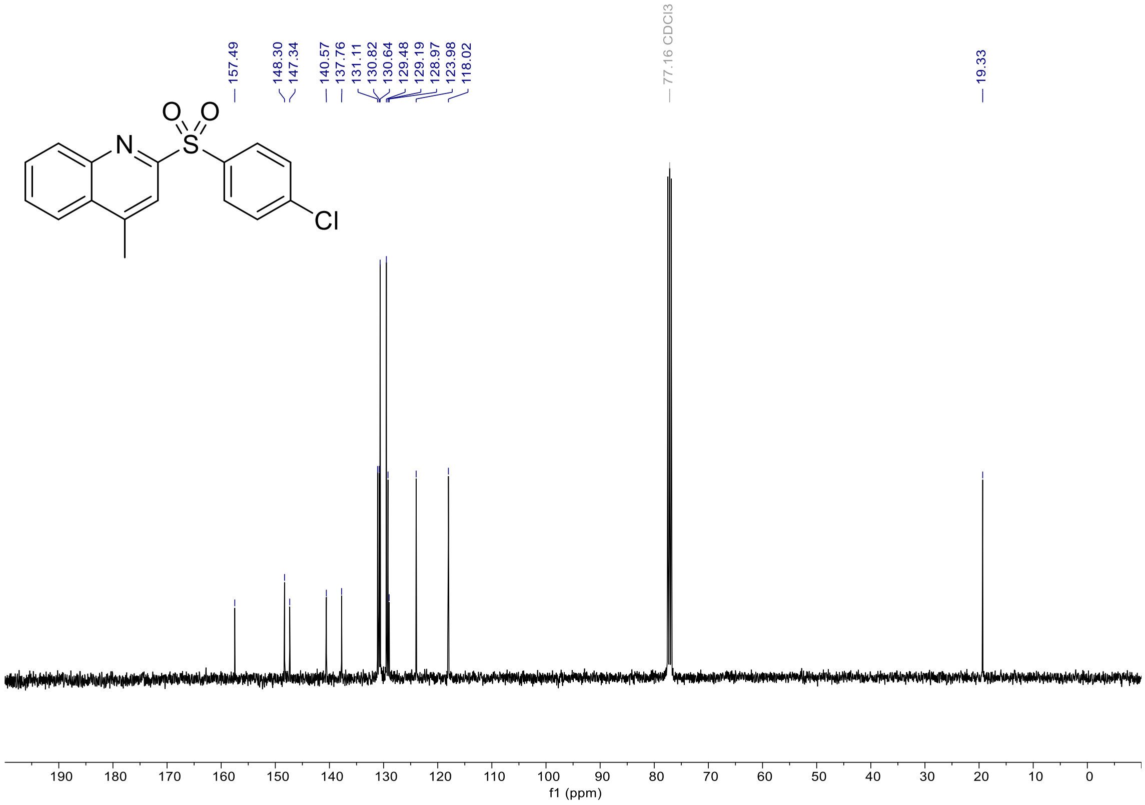

^1H NMR (400 MHz, $CDCl_3$) (**3p**)

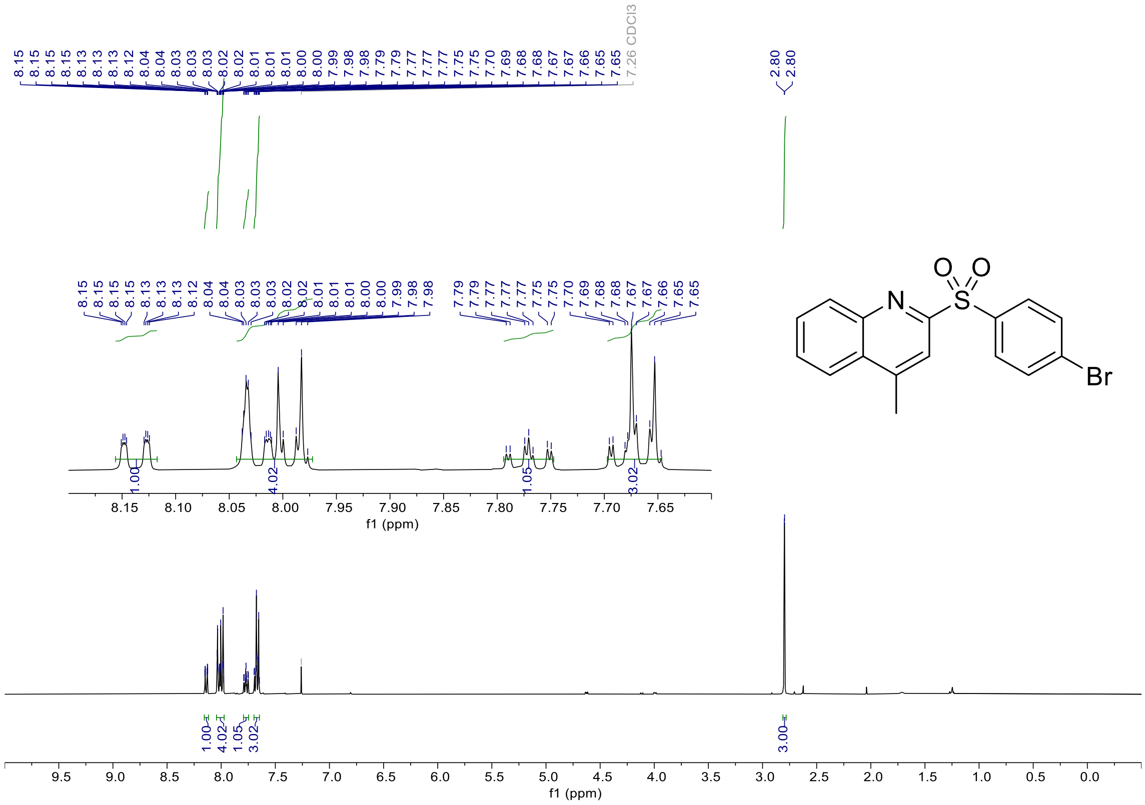

^{13}C NMR (101 MHz, $CDCl_3$) (**3p**)

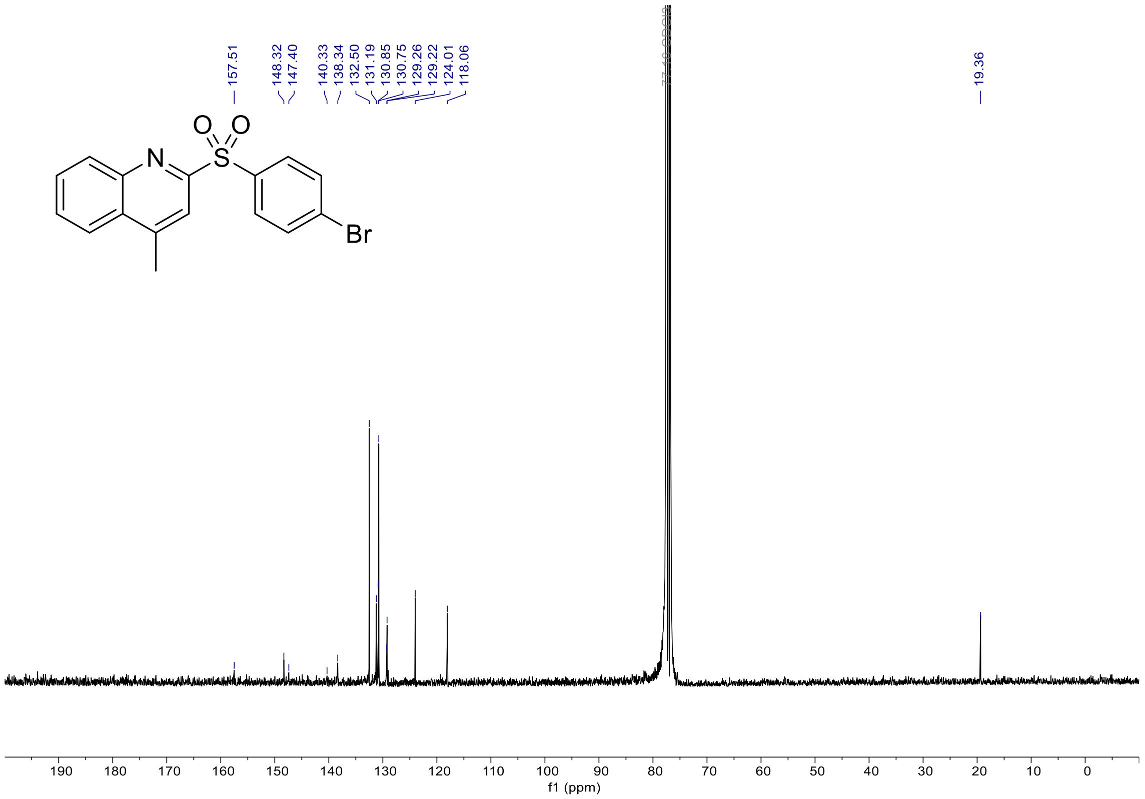

^{1}H NMR (400 MHz, $CDCl_3$) (**3q**)

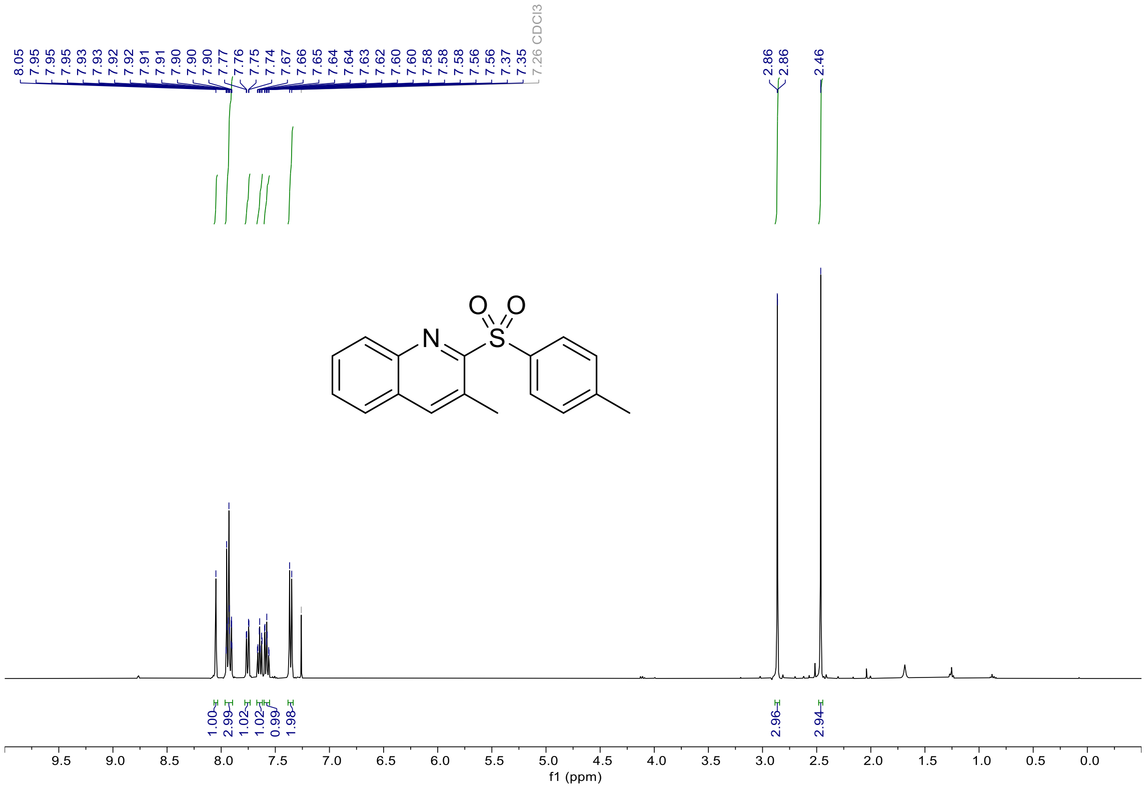

^{13}C NMR (101 MHz, $CDCl_3$) (**3q**)

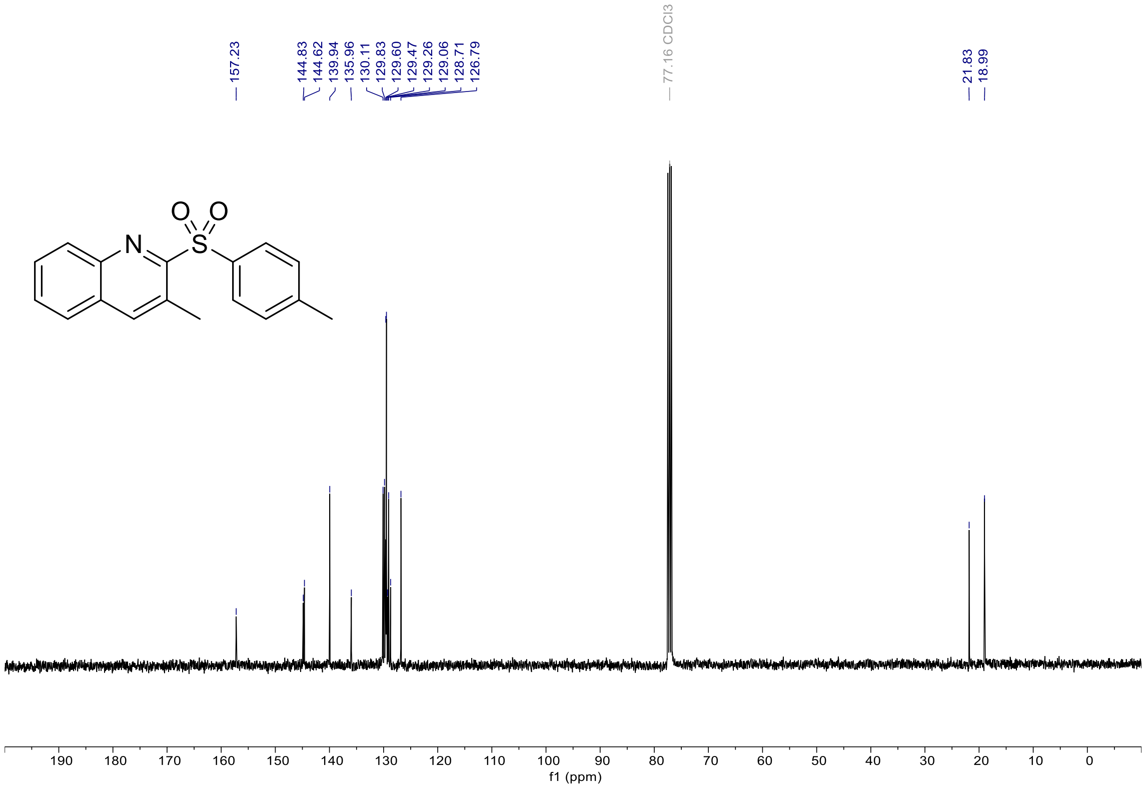

^{1}H NMR (400 MHz, CDCl$_3$) (**3r**)

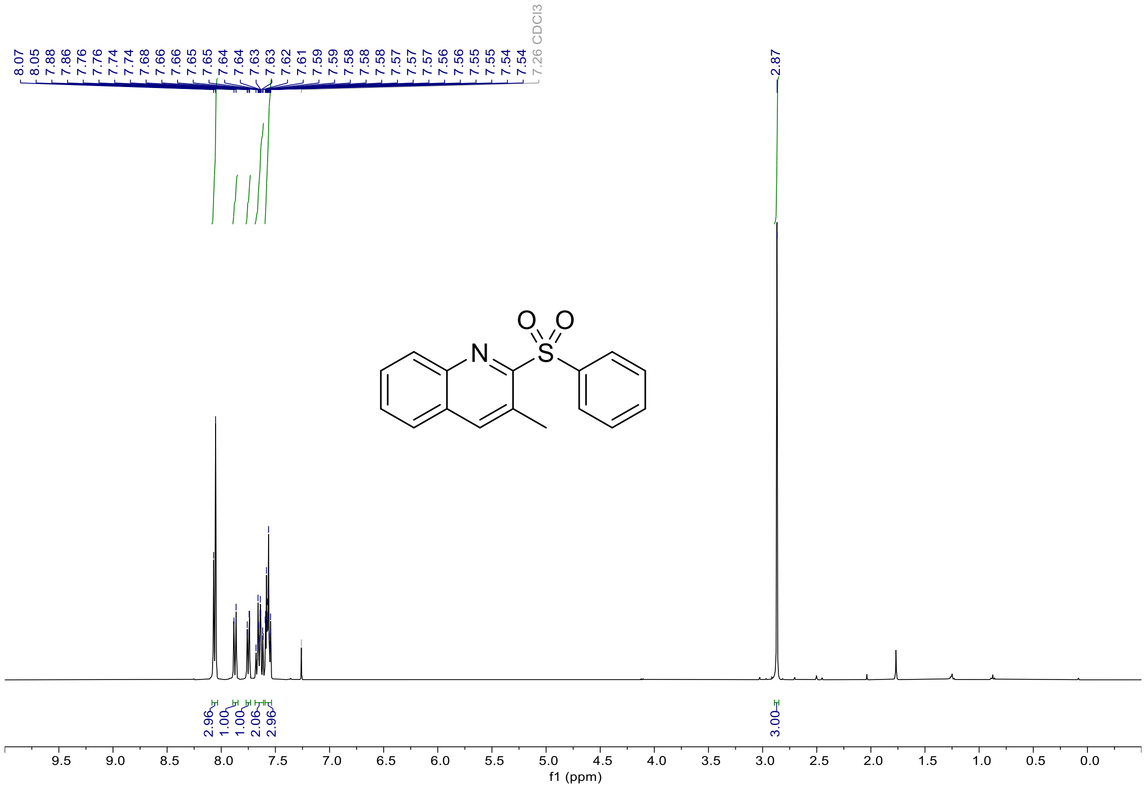

^{13}C NMR (101 MHz, CDCl$_3$) (**3r**)

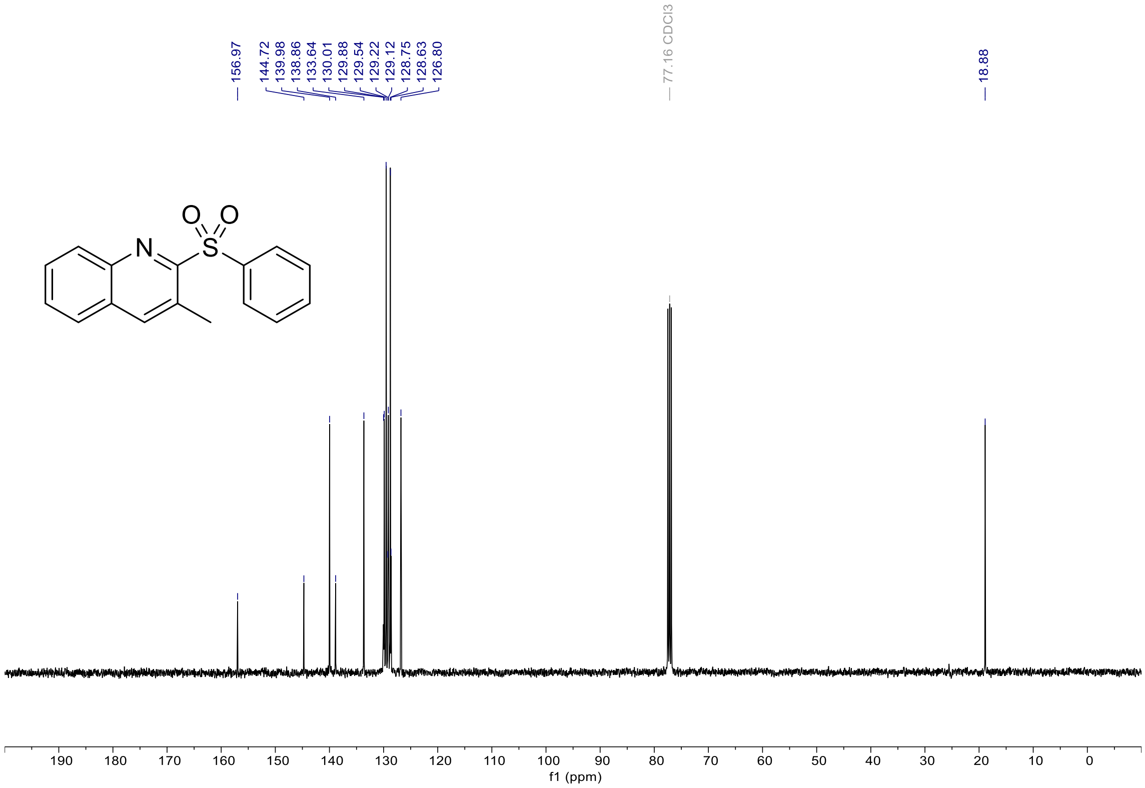

^{1}H NMR (400 MHz, $CDCl_3$) (**3s**)

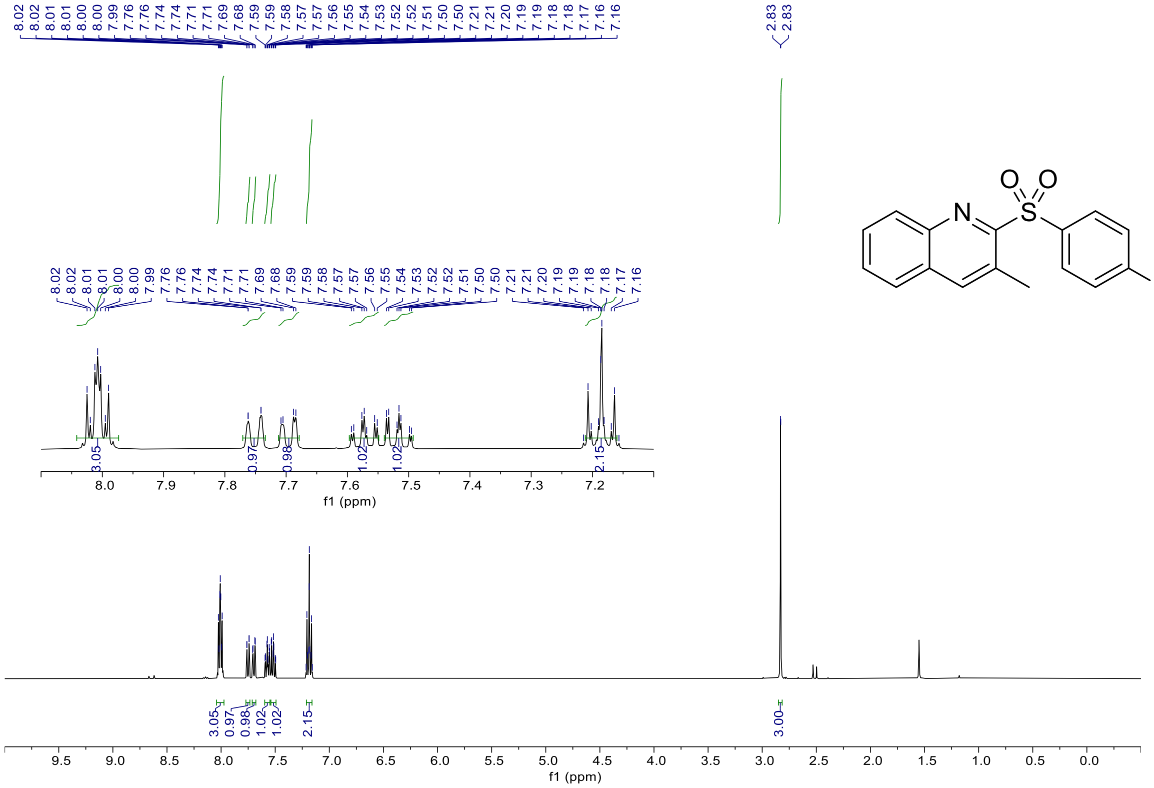

^{13}C NMR (101 MHz, $CDCl_3$) (**3s**)

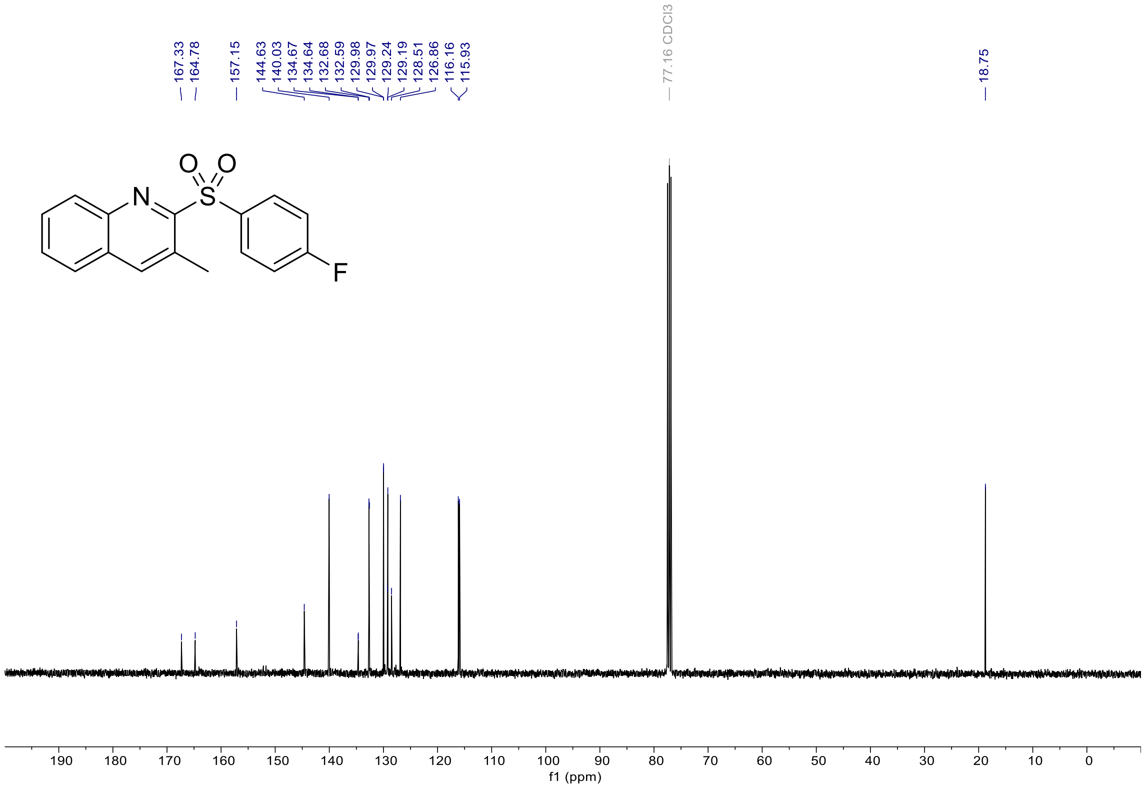

^{19}F NMR (377 MHz, $CDCl_3$) (**3s**)

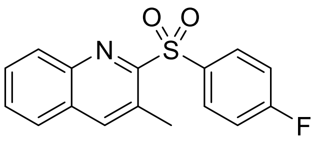

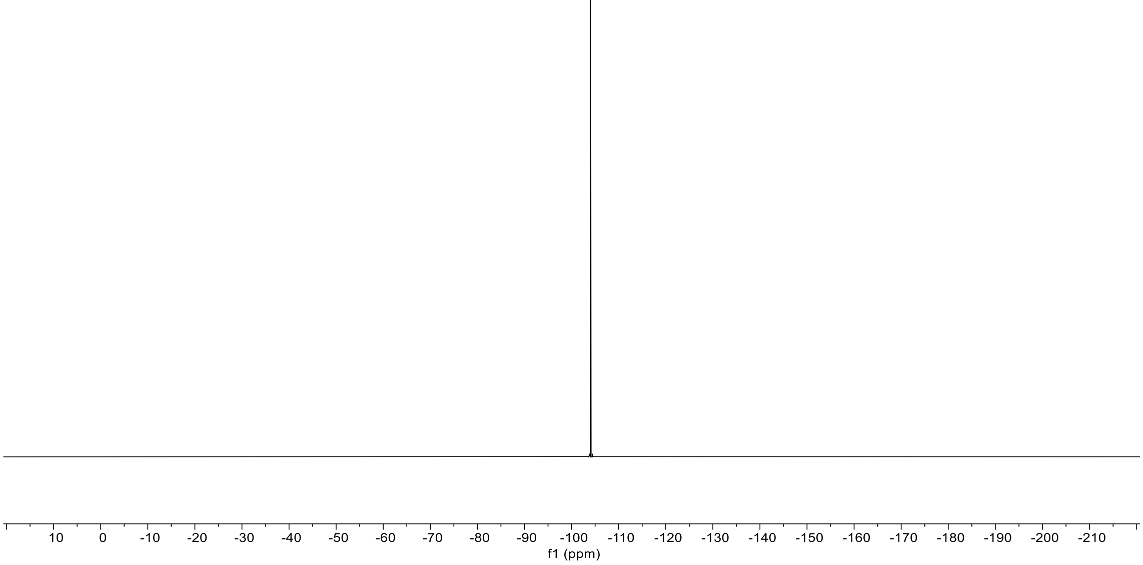

^{1}H NMR (400 MHz, $CDCl_3$) (**3t**)

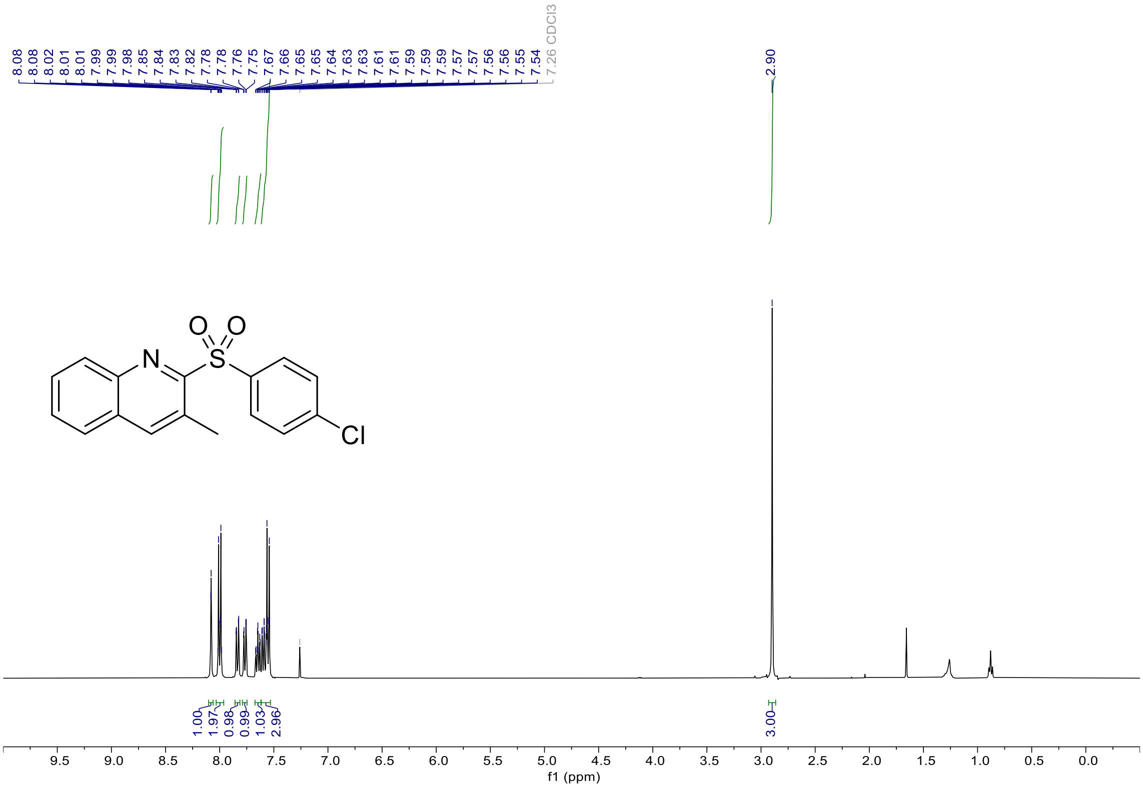

^{13}C NMR (101 MHz, $CDCl_3$) (**3t**)

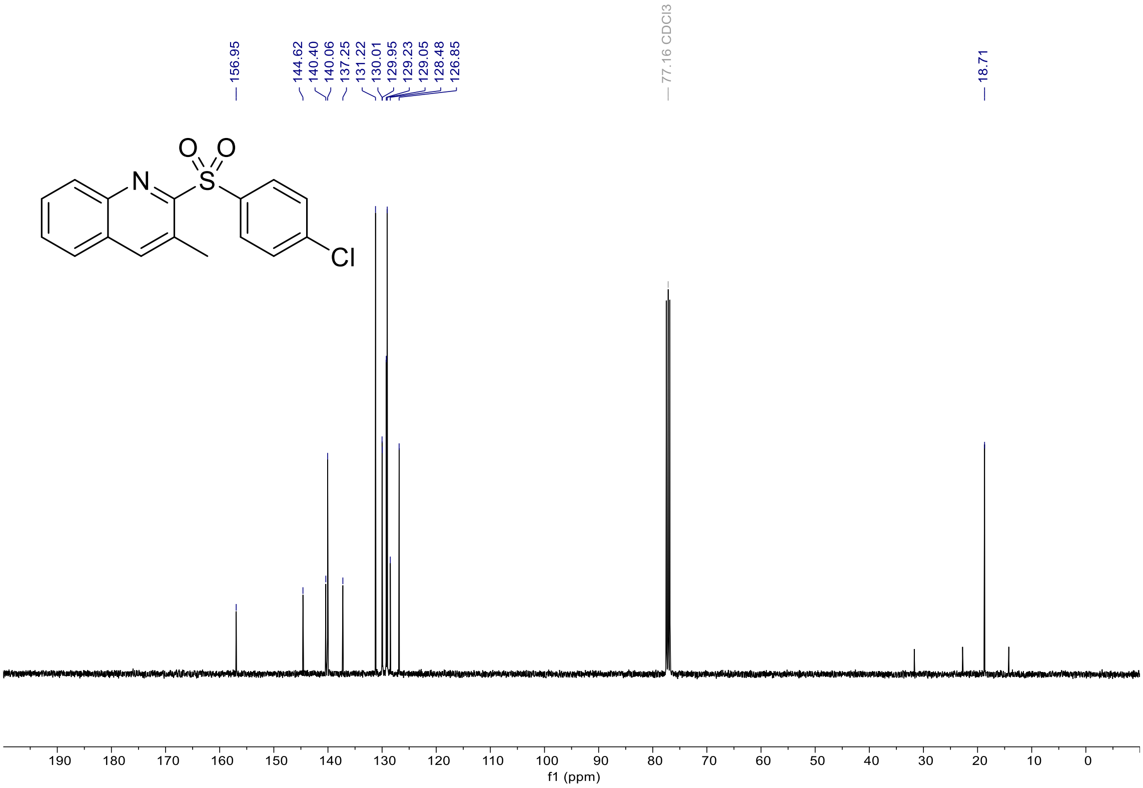

^1H NMR (400 MHz, $CDCl_3$) (**3u**)

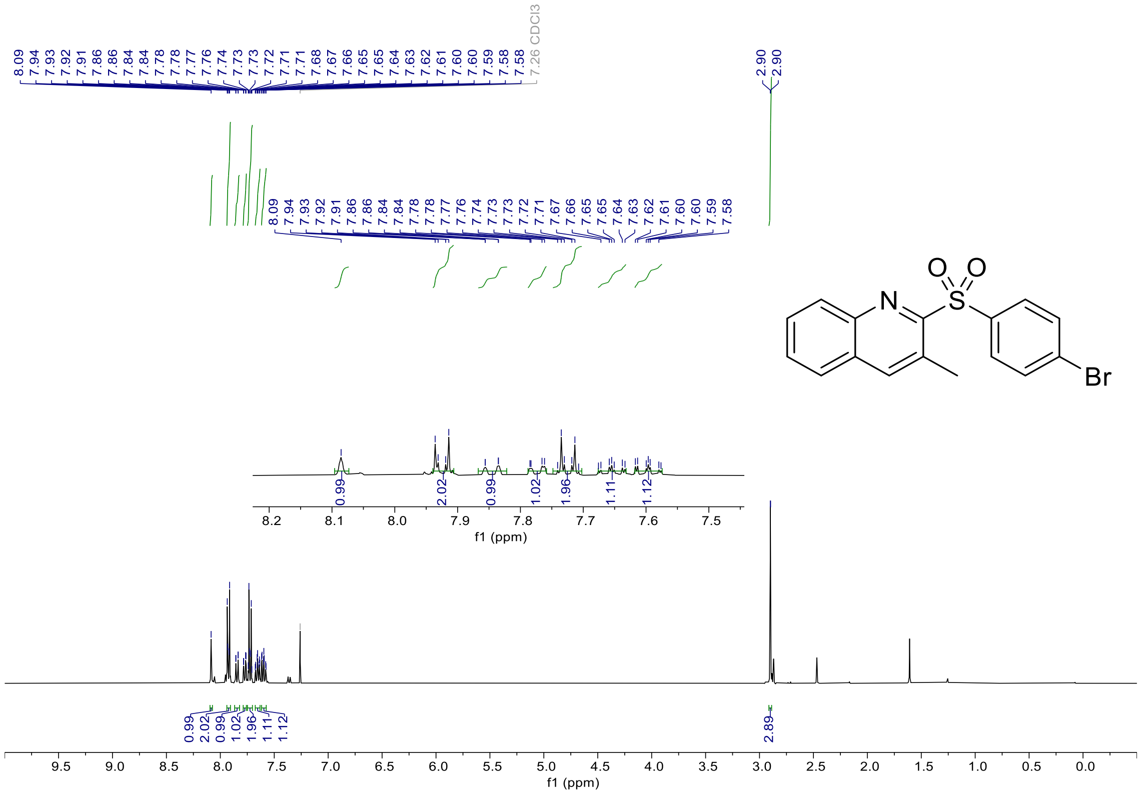

^{13}C NMR (101 MHz, $CDCl_3$) (**3u**)

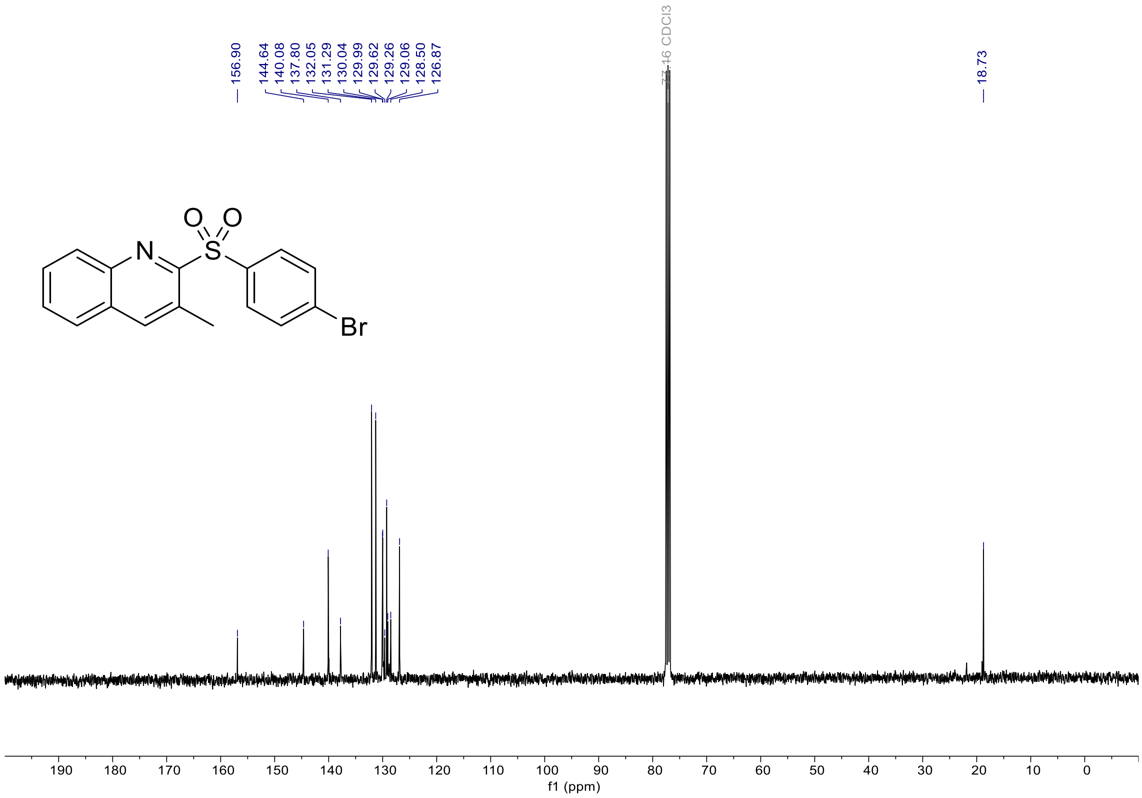

H NMR (400 MHz, CDCl$_3$) (**3v**)

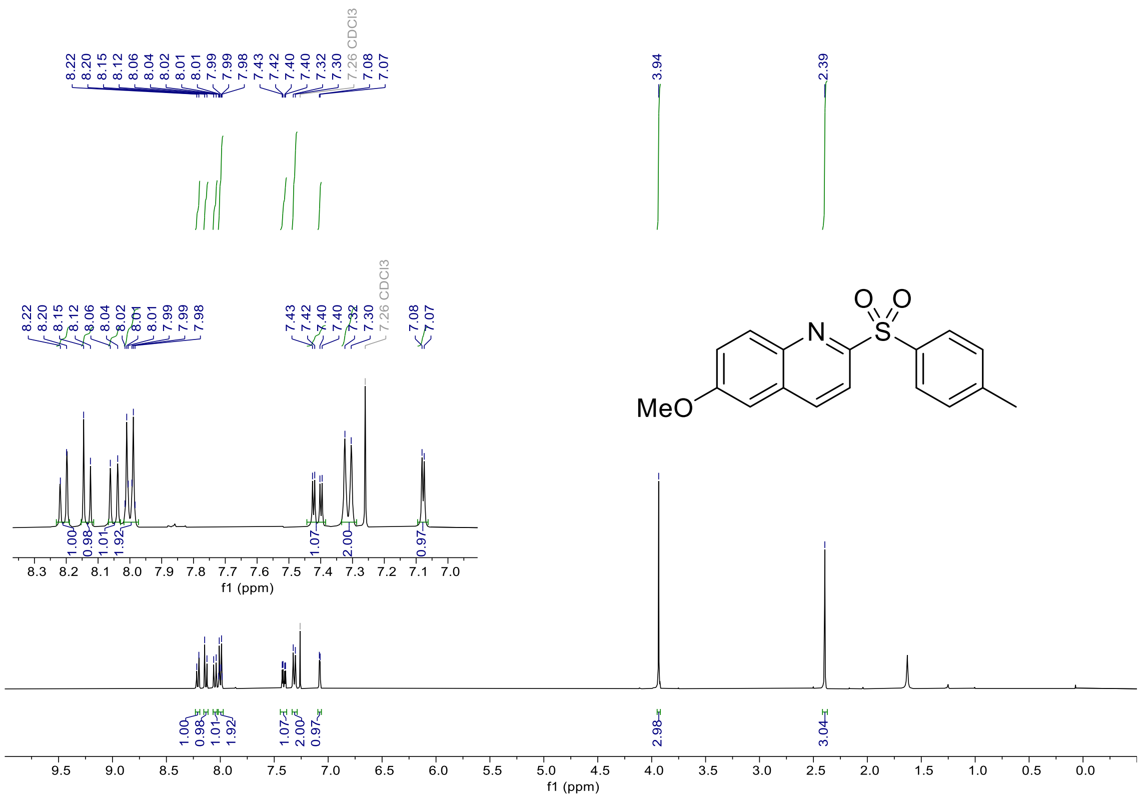

^{13}C NMR (101 MHz, CDCl$_3$) (**3v**)

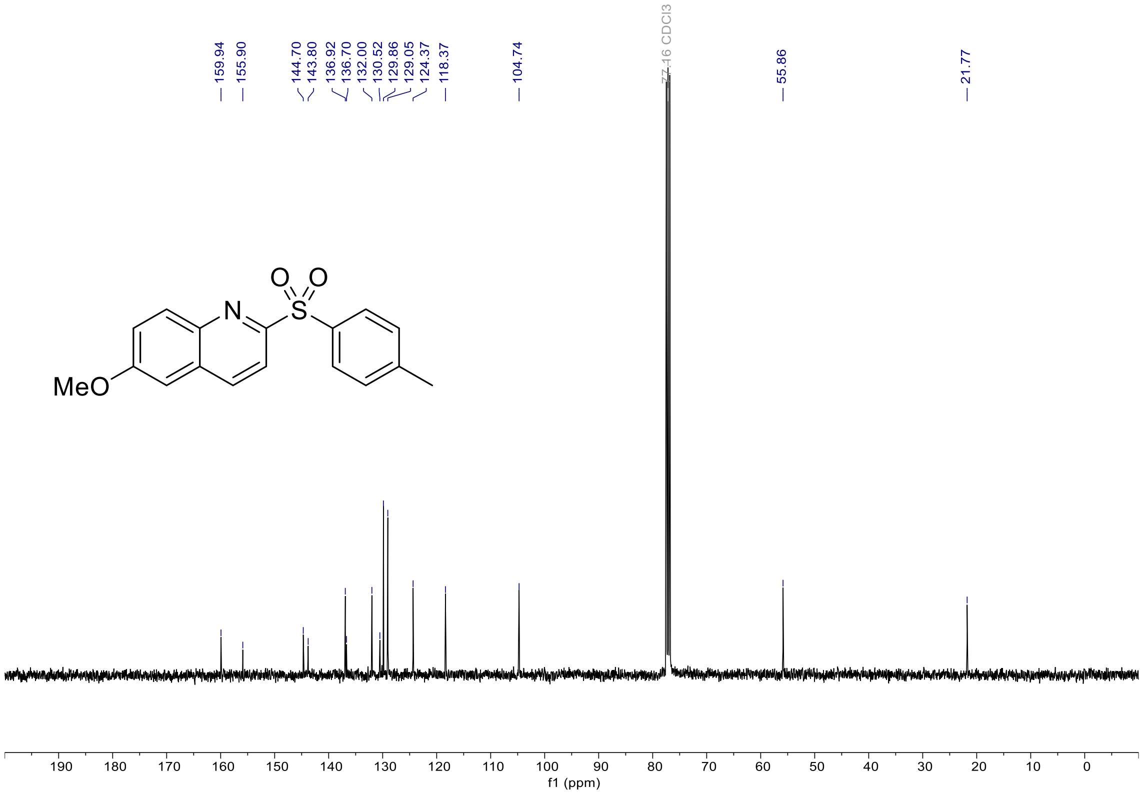

^{1}H NMR (400 MHz, $CDCl_3$) (**3w**)

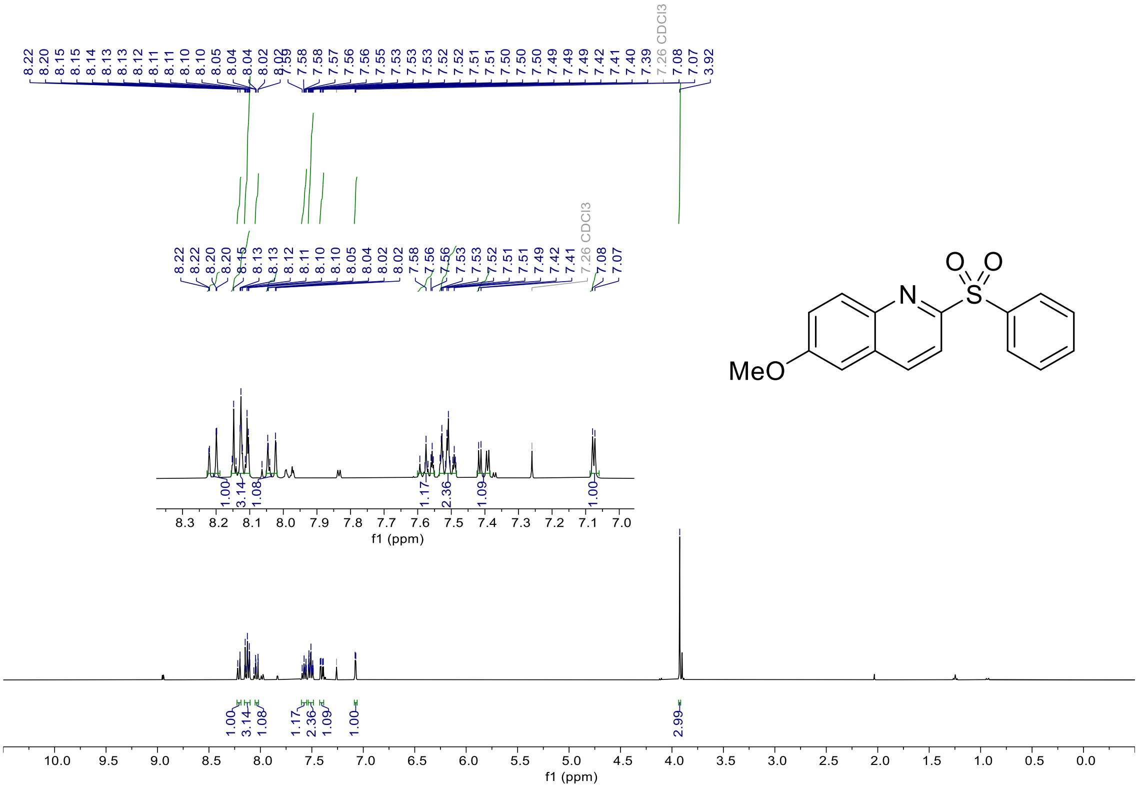

^{13}C NMR (101 MHz, $CDCl_3$) (**3w**)

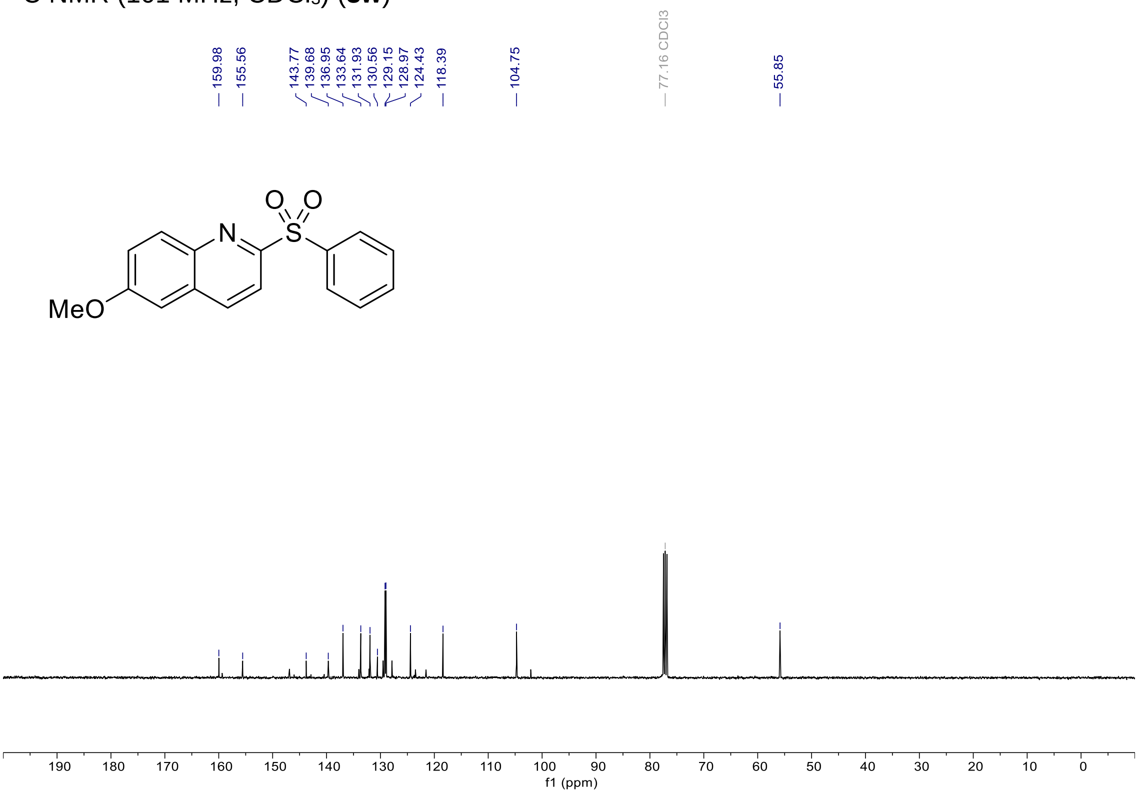

^{1}H NMR (400 MHz, $CDCl_3$) (**3x**)

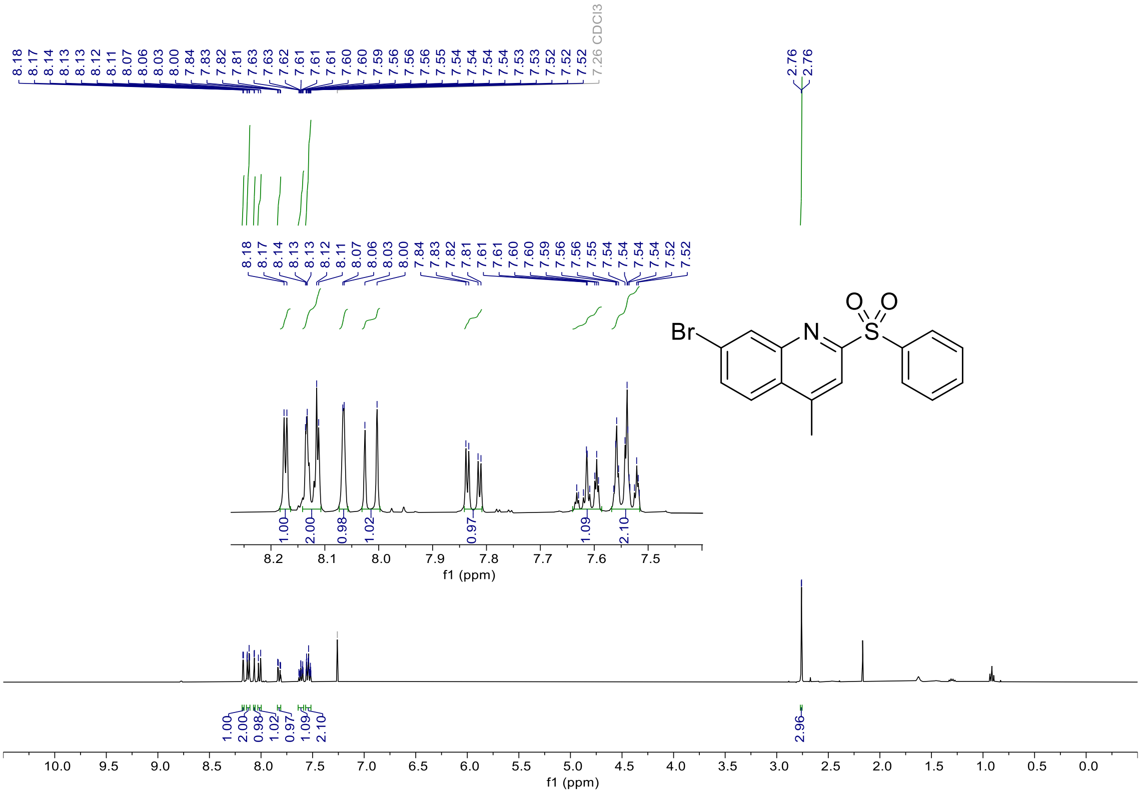

^{13}C NMR (101 MHz, $CDCl_3$) (**3x**)

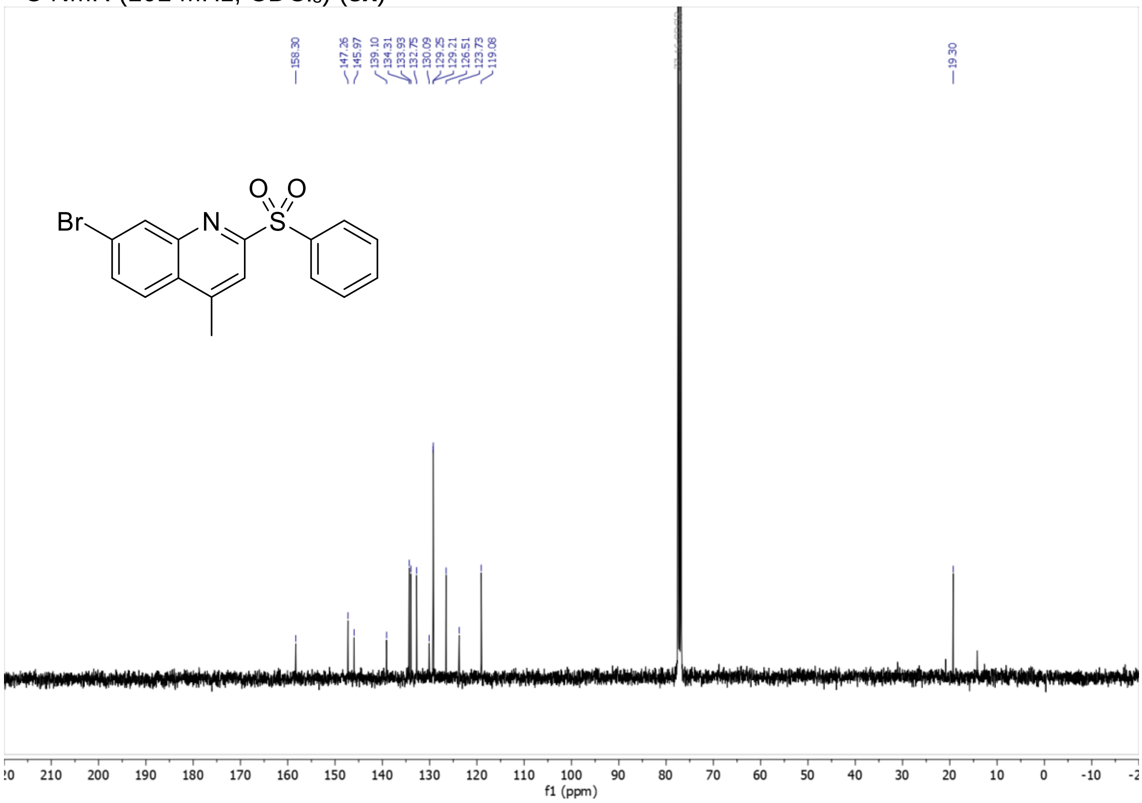

^{1}H NMR (400 MHz, $CDCl_3$) (**3y**)

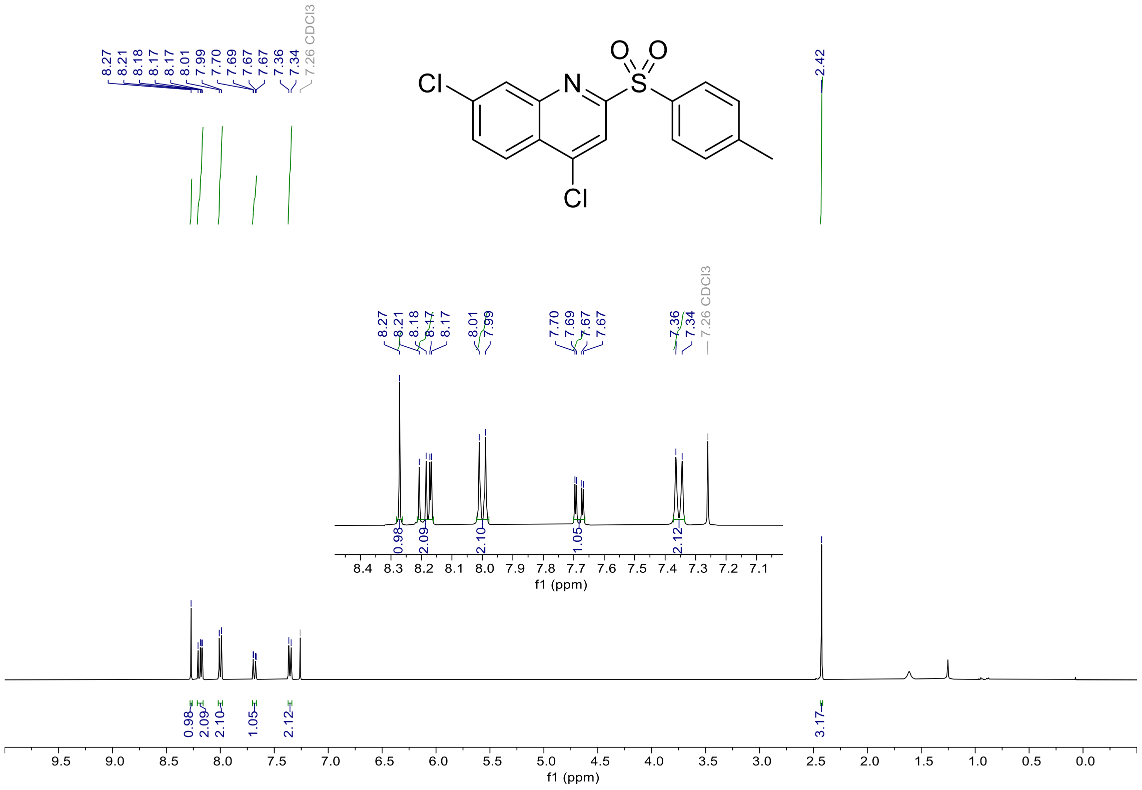

^{13}C NMR (101 MHz, $CDCl_3$) (**3y**)

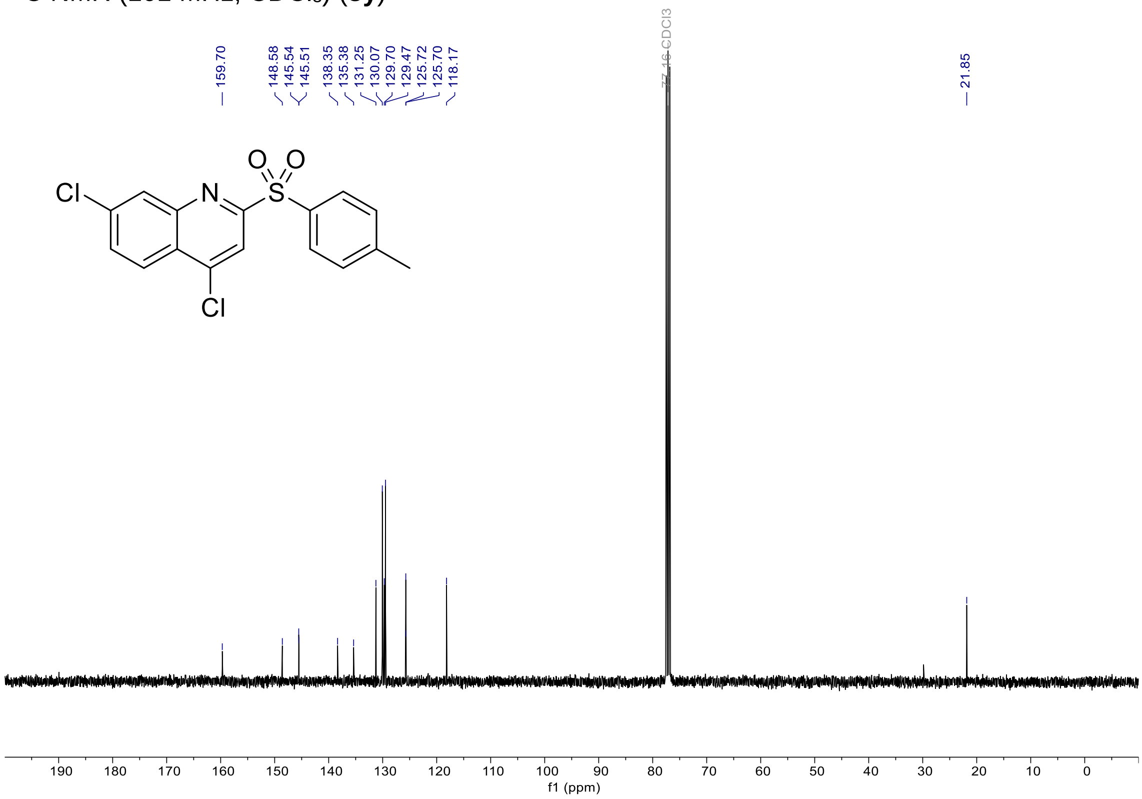

Supplement: Supplementary file 1 — Supplementary Material [file CSSC-18-e202501779-s001.pdf]
